# Association between human blood metabolome and the risk of gastrointestinal tumors

**DOI:** 10.1371/journal.pone.0304574

**Published:** 2024-05-30

**Authors:** Jiamin Lu, Yuqian Feng, Kaibo Guo, Leitao Sun, Shanming Ruan, Kai Zhang

**Affiliations:** 1 The First Affiliated Hospital of Zhejiang Chinese Medical University (Zhejiang Provincial Hospital of Chinese Medicine), Hangzhou, China; 2 The First School of Clinical Medicine, Zhejiang Chinese Medical University, Hangzhou, China; 3 Hangzhou TCM Hospital of Zhejiang Chinese Medical University (Hangzhou Hospital of Traditional Chinese Medicine), Hangzhou, China; 4 Department of Oncology, Affiliated Hangzhou First People’s Hospital, Zhejiang University School of Medicine, Hangzhou, China; 5 Department of Oncology, The Fourth School of Clinical Medicine, Zhejiang Chinese Medical University, Hangzhou, China; 6 Anji Traditional Chinese Medical Hospital, Huzhou, Zhejiang, China; Universite du Quebec a Montreal, CANADA

## Abstract

**Background:**

The prevalence of gastrointestinal tumors continues to be significant. To uncover promising therapeutic targets for these tumors, we rigorously executed a Mendelian randomization (MR) study to comprehensively screen the blood metabolomes for potential causal mediators of five frequently encountered gastrointestinal tumors (Liver Cancer, Colorectal Cancer, Esophageal Cancer, Gastric Cancer and Pancreatic Cancer).

**Methods:**

We selected a comprehensive set of 137 distinct blood metabolites derived from three large-scale genome-wide association studies (GWASs) involving a total of 147827 participants of European ancestry. The gastrointestinal tumors-related data were obtained from a GWAS conducted within the Finnish study. Through meticulous MR analyses, we thoroughly assessed the associations between blood metabolites and gastrointestinal tumors. Additionally, a phenome-wide MR (Phe-MR) analysis was employed to investigate the potential on-target side effects of metabolite interventions.

**Results:**

We have identified 1 blood metabolites, namely isovalerylcarnitine (OR_log10_: 1.01; 95%CI, 1.01–1.02; P = 1.81×10^−7^), as the potential causal mediators for liver cancer. However, no potential pathogenic mediators were detected for the other four tumors.

**Conclusions:**

The current systematic MR analysis elucidated the potential role of isovalerylcarnitine as a causal mediator in the development of liver cancer. Leveraging the power of Phe-MR study facilitated the identification of potential adverse effects associated with drug targets for liver cancer prevention. Considering the weighing of pros and cons, isovalerylcarnitine emerges as a promising candidate for targeted drug interventions in the realm of liver cancer prevention.

## Introduction

Due to advancements in treatment options, including the introduction of targeted therapies and immunotherapies, the 5-year relative survival rate for cancers has witnessed a remarkable improvement. It has escalated from 49% for diagnoses made in the mid 1970s to 68% for diagnoses made in the period of 2012–2018, However, we cannot disregard the fact that gastrointestinal tumors comprise approximately 18% of all cancer cases and account for 28% of all cancer-related fatalities, ranking No.2 in terms of incidence and No.1 in terms of mortality [1]. Given the substantial financial and human implications tied to the failures of clinical trials, it becomes a paramount strategic objective to enhance the prevention and screening of gastrointestinal tumors while identifying potential therapeutic targets.

The swift advancement of human metabolomics has ignited a revolutionary wave in personalized diagnosis and treatment [2]. This stems from the capacity of metabolomics to offer a direct functional assessment of an organism’s physiological state, unveiling the metabolic alterations associated with diseases [3, 4]. Furthermore, human metabolomics can aid in the identification of novel drug targets by pinpointing pathological metabolic changes that are causally linked to disease phenotypes or outcomes [5, 6]. With the continuous advancement of high-throughput sequencing (HTS) technology, numerous genome-wide association studies (GWASs) have made remarkable strides in identifying genetic determinants underlying the intricate human metabolome [7–9]. As a result, we now have the capability to explore novel and dependable drug targets for the treatment of gastrointestinal tumors, utilizing both genetic and metabolomic approaches through the application of Mendelian Randomization (MR). This method is one in which genetic variants are used to investigate the causal relationship of a biomarker on risk of disease. One notable advantage of MR is its ability to minimize the impact of confounding and reverse causation, which are commonly encountered in traditional observational studies [10, 11]. As a result, MR analysis generates more reliable evidence regarding causality, enhancing the reliability of the findings.

In a recent extensive MR analysis, high-density lipoprotein cholesterol (HDL-C) and acetate have emerged as potential causal mediators for breast cancer. Furthermore, a phenome-wide MR (Phe-MR) analysis was conducted to predict metabolite-mediated side effects on various disease traits [12]. These findings provided enhanced confidence in the identification of potential therapeutic targets for gastrointestinal tumors. Initially, a comprehensive two-sample MR analysis was performed to screen 137 circulating metabolites, aiming to identify potential causal mediators for 5 common gastrointestinal tumors, including liver cancer, colorectal cancer, esophageal cancer, gastric cancer, and pancreatic cancer. Subsequently, a Phe-MR analysis involving 118 disease traits was employed to forecast the side effects of metabolite intervention on specific targets.

## Methodology

### Study design

**Fig 1:**
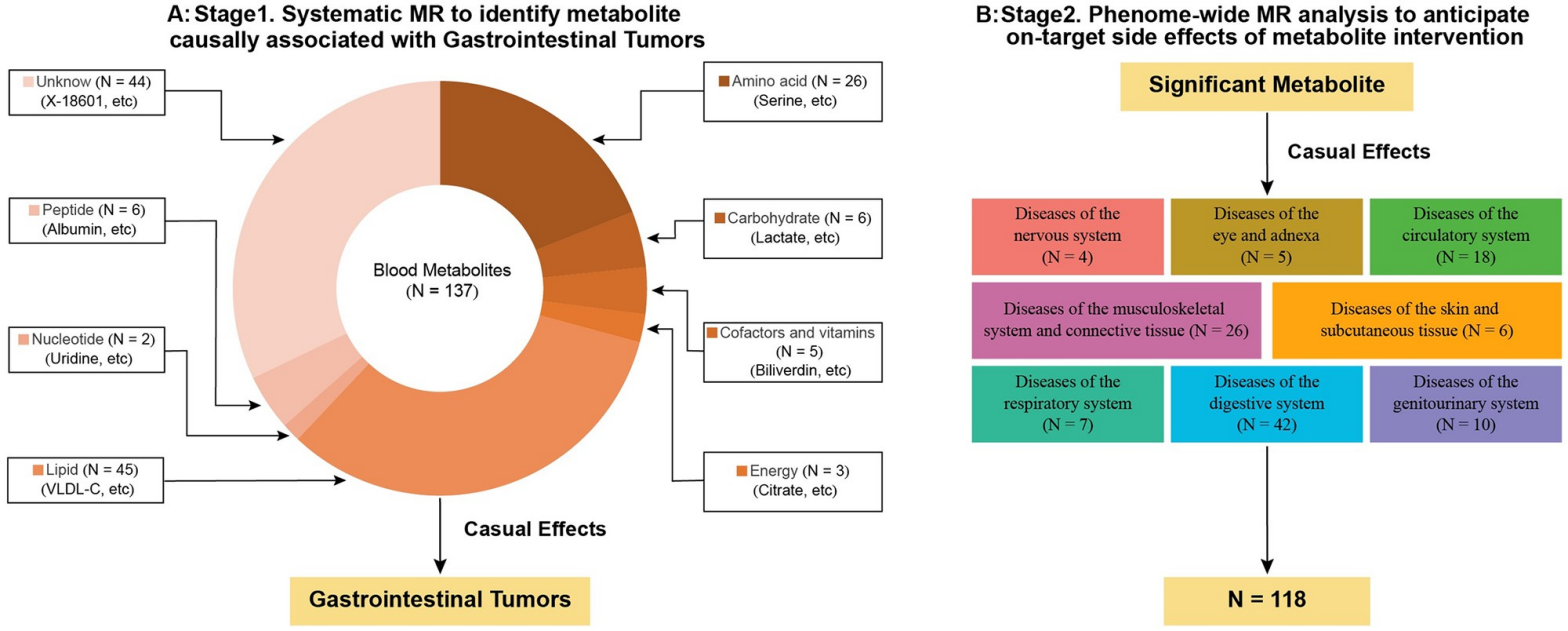
Conceptual framework of two-stage MR study. A): associations between 137 blood metabolites and the risk of gastrointestinal tumors. B): side effects associated with targeting identified metabolites in 118 non-cancer diseases. Each disease belongs to International Classification of Disease (ICD)-10 chapters. A Bonferroni correction was applied to account for the number of metabolites and diseases analyzed.

We performed a two-stage MR analysis of the blood metabolome to determine potential causal factors for gastrointestinal tumors, utilizing three publicly available European-ancestry GWASs Fig 1 [7–9, 13]. Our investigation employed publicly accessible GWAS summary statistics derived from the UK Biobank and FinnGen study. The research conducted by the UK Biobank is endorsed by the North Multicenter Research Ethics Committee, and the FinnGen study has obtained approval from the Ethics Committee of Helsinki University.

### Data source for blood metabolome and gastrointestinal tumors

Full summary statistics for over 800 metabolites are publicly available at 3 Metabolomics GWASs [7–9]. Shen et al [7] conducted a screening of 486 plasma metabolites using a cohort of 7824 participants. The genetic analysis incorporated approximately 3 million single nucleotide polymorphisms (SNPs). In a separate study, Kettunen et al [8] employed a meta-analysis approach, combining a total of 24925 European individuals. This extensive analysis covered 123 blood metabolism indicators and utilized a substantial dataset comprising 12 million SNPs. Furthermore, Nightingale’s biomarker profiling technology [9] was utilized to measure over 200 metabolic biomarkers from a staggering 115078 blood samples obtained from UK Biobank. This dataset encompassed 12 million SNPs. The blood metabolites GWAS dataset mentioned above is openly accessible through the IEU open GWAS database (https://gwas.mrcieu.ac.uk/). Following the exclusion of overlapping metabolites across the three metabolome GWASs, a total of 469 unique metabolites were retained for further analysis.

The genetic association data for 5 prevalent gastrointestinal tumors were obtained from the GWAS performed by the FinnGen study. This dataset is accessible through the IEU GWAS database (https://gwas.mrcieu.ac.uk/). The study included a population size of 218792 individuals, and a total of 16962023 SNPs were detected and analyzed.

### Genetic instrumental of blood metabolites

To meet the assumptions of MR, SNPs that were found to be associated with blood metabolites needed to fulfill certain criteria. Specifically, they should exhibit strong and independent associations (R2 < 0.1 within 500 kb) with the predicted exposures of 5 common gastrointestinal tumors from the published GWAS studies at a genome-wide significance level (P < 5× 10^−8^) [12, 14].

The instrument strength of each SNP was evaluated using the F-statistic, which was calculated using the formula: F = β^2^/SE^2^ [15]. SNPs with an F-statistic greater than 10 were retained, as an F-statistic of 10 or above indicates a low risk of weak instrument bias [16]. In situations where the SNPs identified were not present in the outcome datasets, suitable proxies were utilized instead. These proxies were selected based on a linkage disequilibrium (LD) threshold of R^2^ > 0.8, as determined using LDlink (https://ldlink.nci.nih.gov/) [17]. By employing appropriate proxies, the study ensured that the genetic variants used as instruments adequately represented the SNPs of interest.

To bolster the robustness and reliability of the MR analyses, we excluded metabolites that had fewer than three correlated SNPs across the genome. This selection criterion was employed to ensure that, in specific MR sensitivity analyses, the exposure variable was associated with a minimum of three SNPs [18]. By implementing this criterion, the study aimed to ensure sufficient genetic instruments for the MR analysis, thereby strengthening the validity of the findings.

After excluding weak instrumental variables (IVs) and blood metabolites with fewer than 3 associated SNPs, a total of 137 out of 469 blood metabolites were ultimately included in the MR analysis. S1 Table provides the essential information for these 137 blood metabolites. Additionally, detailed information on the SNPs predicting blood metabolites associated with gastrointestinal tumors can be found in S2 Table.

### Statistical analysis

We employed three primary MR methods to investigate the causal effects of 137 blood metabolite levels on the risk of gastrointestinal tumors: inverse-variance weighted (IVW), MR-Egger, and Weighted median. The IVW analysis, utilizing multiple genetic variants, calculated the average ratio estimates from each variant to yield an overall causal estimate referred to as the IVW estimate. To assess heterogeneity in the IVW estimates, we conducted the Cochran Q test and calculated the I2 index. Heterogeneity was considered significant if I^2^ > 50%, and SNPs exhibiting significant differences were further examined through MR-PRESSO analysis for exclusion [19]. The MR-Egger test was conducted as a slight modification to the IVW method. Instead of assuming the intercept term to be zero, the term was estimated during the analysis. A nonzero intercept indicates the possibility of horizontal pleiotropy, suggesting potential bias in the IVW estimate [20]. Weighted median estimator was a valuable tool as it can provide a reliable estimate of the causal effect, even in situations where up to 50% of the information utilized in the analysis originates from genetic variants that may be considered invalid IVs [21]. In addition, we employed Steiger filtering to ensure the directionality of the association between blood metabolites and gastrointestinal tumors. This filtering technique helped eliminate the potential interference of reverse causality, further strengthening the validity of our findings [22].

### Phe-MR analysis for on-target side effects of liver cancer-related metabolites

The Phe-MR analysis was conducted to evaluate the potential on-target side effects associated with hypothetical interventions aimed at reducing the burden of gastrointestinal tumors by targeting identified metabolites. The OpenGWAS database played a crucial role in this analysis, as it provided access to a wide range of data contributed by various organizations and consortia within the global genetics research community. This database includes the analysis of 2514 phenotypes from the UK Biobank, conducted by the Neale lab in round 2.

To ensure the relevance of the analysis, non-disease phenotypes were excluded, and diseases were categorized using the International Classification of Diseases, Tenth Revision (ICD-10) codes. This study excluded gender-specific disease traits, disease characteristics with an uncertain case count, and disease traits with fewer than 500 cases due to data availability and statistical power concerns. Ultimately, the comprehensive phenotype analysis encompassed 8 categories and 118 non-cancer diseases. Subsequently, the Phe-MR analysis was employed to further investigate the potential side effects of targeting the causal mediators associated with gastrointestinal tumors identified in the initial two-sample MR analysis during stage 1 (Fig 1B; S3 Table).

All MR estimates were reported as odds ratios (ORs) accompanied by 95% confidence intervals (CIs) to provide an accurate depiction of the observed outcomes. In the study conducted by Shen et al [7], metabolite concentrations underwent log_10_-transformation to facilitate direct comparison between case-control and MR estimates [23]. Conversely, in the studies conducted by Kettunen et al [8] and UK Biobank [9], the effects of blood metabolites on gastrointestinal tumors were presented as ORs (95% CIs) per 1 standard deviation (SD) change in genetically predicted cytokine levels.

To control for the risk of type I errors in the calculation of numerous correlations, we employed the Bonferroni correction. In stage 1, an observed P-value less than 7.29 × 10^−5^ (0.05/(137 × 5); calculated as 0.05 divided by the product of 137 metabolites and 5 types of cancers) indicated a significant association between the blood metabolite and cancer. Results with P-values between 7.29 × 10^−5^ and 0.05 were regarded as suggestive associations. In stage 2, a P-value below 4.24 × 10^−4^ (derived from 0.05 divided by the product of 118 disease phenotypes and 1 potential pathogenic mediator of liver cancer) suggested potential on-target side effects of blood metabolites. All statistical analyses were conducted using R (version 4.2.2) and involved the packages ComplexHeatmap, ggplot2, gridBase, gridExtra, RColorBrewer, RGraphics, and TwoSampleMR.

## Results

### Strength of genetic instruments for blood metabolites

A total of 137 unique blood metabolites are included in the present MR study (S1 Table), and the detailed information on genetic instruments for each blood metabolite is shown in S2 Table. The F-statistics for all selected instruments ranged from 23 to 16413 and exceeded the threshold of 10, indicating a low likelihood of weak instrument bias in our study (S3 Table).

### Stage 1 results: Identifying the significant blood metabolites as potential causal mediators of gastrointestinal tumors

**Fig 2:**
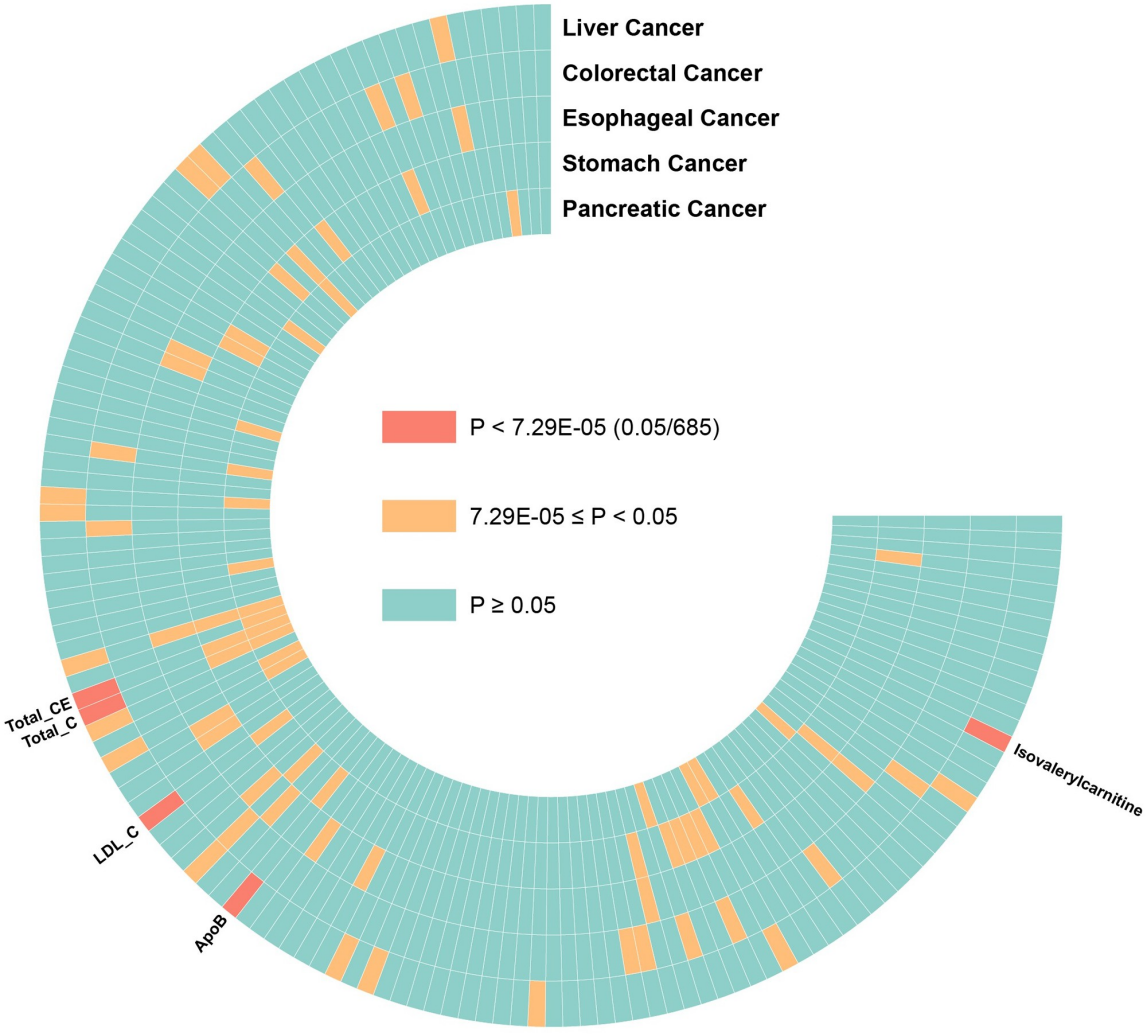
Ring heatmap displaying the associations between blood metabolites and the risk of gastrointestinal tumors. The red represents the Bonferroni-corrected significance threshold (P < 0.05/137/5 = 7.29 × 10^−5^), and the color orange is used to represent the range of P-values between 7.29 × 10^−5^ and 0.05.

**Fig 3:**
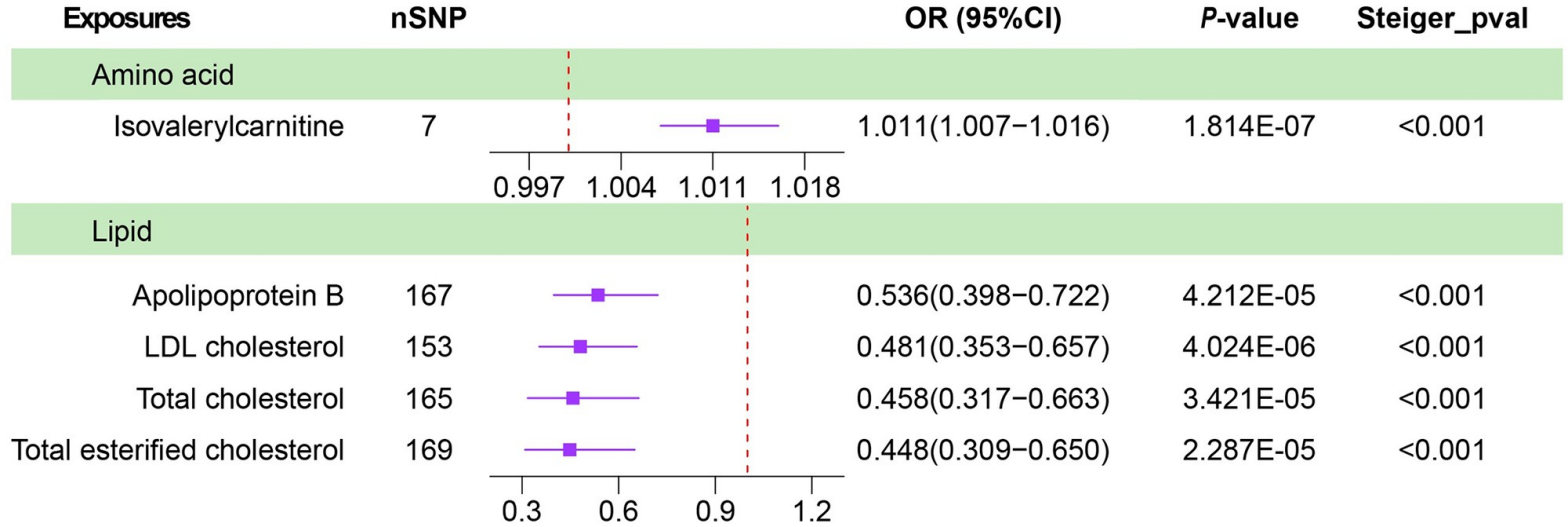
Genetically predicted associations of five blood metabolites significantly associated with risk of liver cancer (Method of IVW and Steiger filtering test).

Using IVW method to estimate the association between 137 blood metabolite and 5 gastrointestinal tumors, the details were shown in Fig 2, S3 and S4 Tables. Among these 137 unique blood metabolites, genetically determined high levels of isovalerylcarnitine (OR_log10_: 1.01; 95%CI, 1.01–1.02; P = 1.81×10^−7^) was significantly associated with an increased risk of liver cancer. Besides, higher levels of Apolipoproteins B (OR_SD_: 0.54; 95%CI, 0.40–0.72; P = 4.21×10^−5^), Low Density Lipoprotein Cholesterol (LDL-C) (OR_SD_: 0.48; 95%CI, 0.35–0.66; P = 4.02×10^−6^), Total Cholesterol (TC) (OR_SD_: 0.46; 95%CI, 0.32–0.66; P = 3.42×10^−5^), and Total Cholesterol Esters (TCE) (OR_SD_: 0.45; 95% CI, 0.31–0.65; P = 2.29×10^−5^) were significantly associated with an low risk of liver cancer (Fig 3). No significant associations were found for 137 blood metabolites with the other 4 gastrointestinal tumors (colorectal, esophageal, gastric and pancreatic). Besides, conducting sensitivity analyses was imperative to evaluate the robustness of our primary findings. MR-Egger test identified the existence of horizontal pleiotropy in genetic variation of Apolipoproteins B, LDL-C, TC, and TCE, although IVW suggested a strong association between these 4 blood metabolites and liver cancer (Table 1), we excluded them in Phe-MR analysis. In conclusion, we have identified a significant correlation between isovalerylcarnitine and a significantly elevated risk of liver cancer.

**Table 1:** Heterogeneity and pleiotropy analyses for blood metabolites having etiologic associations with liver cancer risks.

Metabolite	Inverse variance weighting			MR-Egger		MR-PRESSO
	Q	Q *P*-val	I^2^	intercept	intercept *P*-val	Global Test *P*-val
Isovalerylcarnitine	5.61	0.47	0	0	0.74	0.27
Total C	219.58	<0.01	25%	0.05	1.98E-03	0.14
Total CE	226.05	<0.01	26%	0.05	1.90E-03	0.96
LDL C	179.61	0.06	15%	0.04	1.68E-02	0.08
Apolipoprotein B	191.62	0.08	13%	0.04	1.68E-02	0

### Stage 2 results: Phe-MR analysis associations of identified blood metabolite on the risk of 118 diseases

Phe-MR analysis was conducted to systematically evaluate the impact of isovalerylcarnitine on the risks of 118 non-tumor-related diseases, with the aim of exploring potential associated side effects. However, none of the associations reached the Bonferroni-corrected significance threshold of P = 4.24 × 10^−4^ (0.05/118; 118 diseases) (S5 Table). This suggested that isovalerylcarnitine may not exhibit a significant association with the 118 common non-tumor-related diseases. The full set of raw data used in the analysis can be found in S6 Table.

## Discussion

The advancing maturity of High-Throughput Sequencing (HTS) and the rapid progress in metabolomics have furnished robust technical foundations and ensured reliable data for MR analyses [24]. This MR study aimed to investigate potential therapeutic targets for digestive tumors and assess the safety of these targets. Through a comprehensive analysis involving 137 blood metabolites, we investigated their associations with five commonly occurring gastrointestinal tumors: liver cancer, colorectal cancer, esophageal cancer, gastric cancer, and pancreatic cancer. In this study, we addressed the challenge of multiple comparisons arising from our extensive analysis, an issue that exacerbates the risk of Type I errors. We employed the Bonferroni correction method, a decision rooted in the imperative to maintain strict control over false positives, which could otherwise lead to erroneous conclusions of statistical significance [25]. The Bonferroni method, while conservative, was deemed the most suitable for our analysis as it aligns with our priority for ensuring a high degree of confidence in our findings. This rigorous correction involves adjusting our alpha level by dividing it by the number of hypotheses tested, thereby ensuring that only associations with substantial evidence are deemed significant [26]. While this approach heightens the stringency of our acceptance criteria, it does so with the benefit of minimizing misleading results. We acknowledge that this approach may diminish statistical power and the potential for Type II errors; however, these trade-offs are acceptable within our study’s framework, given that the cost of a false discovery would be considerable. Although the Bonferroni method is conservative, its wide acceptance and prevalent use in our field are well-documented, as evidenced by numerous studies [12, 14, 27]. By employing the Bonferroni correction, our research underscores the viability of isovalerylcarnitine as a compelling therapeutic target in the treatment of liver cancer. Our data suggest that lowering isovalerylcarnitine levels could potentially reduce the risk of developing liver cancer, without significant adverse effects on other diseases.

In recent times, there has been a growing interest among researchers in assessing the potential of carnitine isovalerate as a cancer biomarker. An inverse correlation has been established between elevated levels of circulating isovalerylcarnitine and the risk of developing lung cancer, as evidenced by a Mendelian randomization study [23]. Additionally, in a population-based case-control study examining women with endometrial cancer and control subjects, serum metabolic profiling revealed a significant negative correlation between carnitine isovalerate and the incidence of endometrial cancer [28].

Isovalerylcarnitine falls into the category of acylcarnitines, which are compounds involved in the intricate energy metabolism processes within the human body. Derived from the amino acid leucine, isovalerylcarnitine assumes a crucial role in the breakdown and transportation of fatty acids into the mitochondria, where they are efficiently utilized for energy production [29].

Several studies have shed light on the alterations in acylcarnitines associated with liver cancer. Jadegoud Yaligar et al. [30] observed notable acylcarnitine signaling in a transgenic mouse model of hepatocellular carcinoma (HCC), while the control group’s liver exhibited no such acylcarnitine presence. In a separate investigation by Yong Zhang et al. [31], concentrations of acylcarnitines in blood samples from individuals with HCC and cirrhosis were meticulously collected and analyzed. This observational study not only revealed the potential use of acylcarnitines and certain relevant ratios in constructing a differential diagnosis model but also identified short-chain acylcarnitines as a noteworthy risk factor for HCC.

A study conducted by Naoto Fujiwara et al. [32] revealed that HCC can adapt to a lipid-rich environment and promote carcinogenesis through the accumulation of acylcarnitines in patients with Fatty Liver (FL). Consequently, elevated levels of acylcarnitines may serve as valuable biomarkers for the early detection of HCC in FL patients. Additionally, a case-control study by Hiroaki Takaya et al. [33] indicated that, among individuals without FL, the levels of blood acylcarnitines in HCC patients were significantly lower compared to non-HCC patients, such as hexanoylcarnitine, octanoylcarnitine, decanoylcarnitine, etc. This suggested that acylcarnitines might play a protective role in non-FL patients with HCC. Significantly, no substantial disparity in the blood concentrations of isovalerylcarnitine, which also falls within the acylcarnitine category, was observed between the two cohorts of patients. Nonetheless, our MR analysis revealed the pivotal role of isovalerylcarnitine in the induction of hepatic carcinogenesis. Multiple factors could potentially account for this intriguing finding. Firstly, it is worth noting that the case-control study encompassed an Asian population, while our MR analysis incorporated a European cohort, and disparities in genetic predisposition to liver cancer may arise due to variances in ethnic groups [34]. Secondly, owing to the database’s limitations, the precise histological subtypes of the liver cancer patients remain undisclosed. Thereby, our analysis encompasses a cohort of generalized liver cancer patients, encompassing both HCC and intrahepatic cholangiocarcinoma (ICC).

With regards to LDL-C, TC, and TCE, the findings of our MR study suggested that elevated blood lipid levels may impede the progression of liver cancer. However, it is worth noting that the significant MR-Egger intercept test indicates the potential presence of horizontal pleiotropy, which could introduce bias to the IVW estimates. Consequently, we refrain from considering lipids as a viable therapeutic target for gastrointestinal tumors. Nevertheless, the intricate interplay between lipids and liver cancer remains undeniable.

As elucidated by Bichitra Paul et al. [35], lipids represented a complex and diverse group of molecules that wield pivotal roles in numerous physiological processes, exerting significant influence over the initiation, advancement, and sustenance of various cancers. Cholesterol, being the fundamental building block of lipids, orchestrates these critical metabolic processes. Within the liver, alterations in cholesterol levels are frequently observed as both a consequence and causal factor in the context of viral hepatitis, fatty liver disease, and primary liver cancer.

Cholesterol is predominantly synthesized within the body’s hepatic realm. The intricate process of lipid synthesis involves a cascading series of enzymatic reactions, comprising over 20 enzymes, collectively referred to as the HMG-CoA reductase pathway. This pathway encompasses numerous intermediate compounds and enzymes that facilitate the transformative conversion of precursor molecules into cholesterol. The regulation of this process is governed by feedback mechanisms, safeguarding the equilibrium of cholesterol homeostasis within the body [36]. As for adults, the typical reference ranges for TC levels hover around the realm of 125–200 mg/dL (3.2–5.2 mmol/L) [37]. The relationship between cholesterol and the realm of cancer is a subject of profound complexity and ongoing scrutiny. Some investigations posited the notion that elevated cholesterol levels may potentially correlate with an augmented susceptibility to certain malignancies, including, including breast [38], colon [39], and prostate cancer [40].

Obesity, as commonly understood, stands as an independent risk factor contributing to the incidence and mortality rates of primary liver cancer [41]. This correlation may stem from the fact that dietary cholesterol triggers oncogenic mutations through inflammation and perturbations in gene expression associated with metabolism, thereby hastening the progression of hepatocellular carcinoma HCC [42]. Conversely, hypercholesterolemia appeared to be associated with well-preserved hepatic function and reduced mortality [43]. Evidence from animal experiments had revealed that mice with heightened serum cholesterol levels developed fewer and smaller tumors upon injection of hepatoma cells. Such findings suggested a plausible connection between cholesterol and natural killer (NK) cells, wherein cholesterol accumulation within NK cells may activate their effector functions against hepatoma cells [44]. A recent nationwide population study showed that low serum cholesterol (LDL-C, TC, and triglycerides) in patients was significantly associated with an increased risk of developing HCC [45]. This was also supported by a multi-center clinical research where people with low LDL cholesterol levels (<100mg/dL) had a higher risk of cancers of gastrointestinal tumors [46]. Therefore, the association between serum cholesterol levels and the risk of hepatocellular carcinoma deserves more in-depth study, even in the presence of confounding factors.

Our study possessed notable strengths. Firstly, we employed three extensive GWASs of blood metabolomics as exposures to estimate their causal impacts on the susceptibility to gastrointestinal tumors within the framework of this systematic MR investigation. Secondly, we applied diverse MR analysis methods, each tailored to address horizontal pleiotropy or outliers through distinct approaches. Additionally, we conducted a series of sensitivity analyses to safeguard the robustness of our MR findings against variations in methodologies, models, or underlying assumptions. Thirdly, our implementation of Phe-MR analysis enabled us to probe into the causal effects of blood metabolites on a wide spectrum of diseases, thereby facilitating the identification of potential drug targets and aiding in the prediction of on-target side effects.

The recognition of several salient limitations is imperative. Firstly, our MR study was limited to the European population, which overlooks the substantial prevalence of gastrointestinal tumors in Asian [47] and African [48] populations. Investigating potential therapeutic targets for gastrointestinal tumors through blood metabolomics in Asian and African populations could prove highly valuable. Secondly, the blood metabolites employed as exposures were derived from three distinct studies, potentially introducing biases due to variations in sample collection methods and assay instruments. Thirdly, despite our analysis encompassing 469 blood metabolites and including 137 in the MR investigation, it remains indisputable that this selection constitutes only a small fraction of the vast array of blood metabolites. Furthermore, it is important to note that the Phe-MR analysis encompassed a limited number of 118 diseases, which also accounted for only a fraction of the entire spectrum of diseases. Fourth, although Bonferroni correction affirmed the significance of our findings, plasma metabolites potentially related to the risk of gastrointestinal tumors merited similar attention; however, the presence of as many as 75 potential associations precluded a comprehensive analysis, as detailed in S7 Table. Lastly, our study predominantly focused on the overall incidence of gastrointestinal tumors, lacking specific information pertaining to gastrointestinal tumor subtypes. Consequently, future endeavors investigating the relationship between gastrointestinal cancer subtypes and blood metabolomes hold clinical significance, as they would furnish additional insights crucial for targeted prevention and treatment strategies.

## Conclusions

The systematic MR analysis conducted in this study unveiled the potential role of isovalerylcarnitine as a causal mediator in the development of liver cancer. However, further investigation is required through large-scale randomized controlled trials to ascertain the potential negative associations between Apolipoproteins B, LDL-C, TC, TCE and liver cancer. The application of Phe-MR analysis aided in the identification of potential side effects associated with drug targets for the prevention of liver cancer. Considering the advantages and disadvantages, isovalerylcarnitine shows promise as a prospective drug target for the prevention of liver cancer.

## Supporting information

S1 Table Overview of the blood metabolites included in the MR study. (PDF)

S2 Table A) Details of blood metabolite predicting SNPs with liver cancer; B) Details of blood metabolite predicting SNPs with colorectal cancer; C) Details of blood metabolite predicting SNPs with esophageal cancer; D) Details of blood metabolite predicting SNPs with gastric cancer; E) Details of blood metabolite predicting SNPs with pancreatic cancer. (ZIP)

S3 Table A) MR results in liver cancer; B) MR results in colorectal cancer; C) MR results in esophageal cancer; B) MR results in gastric cancer; B) MR results in pancreatic cancer. (PDF)

S4 Table A) Sensitivity analyses in liver cancer; B) Sensitivity analyses in colorectal cancer; C) Sensitivity analyses in esophageal cancer; D) Sensitivity analyses in gastric cancer; E) Sensitivity analyses in pancreatic cancer. (PDF)

S5 Table Results of Phe-MR (isovalerylcarnitine and 118 diseases). (PDF)

S6 Table Online sourced raw data catalogue. (PDF)

S7 Table Potentially relevant plasma metabolites. (PDF)
