# Supplementary material for: Association between human blood metabolome and the risk of gastrointestinal tumors
Source: PLoS One. 2024 May 30;19(5):e0304574. doi: 10.1371/journal.pone.0304574 (PMC11139295; doi:10.1371/journal.pone.0304574)
Supplement: S1 Table — (PDF) [file pone.0304574.s001.pdf]

Supplementary Table 1. Overview of the blood metabolites included in the MR study.

Metabolite	Super-pathway	SNPs used in MR study	Sample size	Population
Valine	Amino acid	49	115048	European
Tyrosine	Amino acid	76	114911	European
Tryptophan betaine	Amino acid	4	7439	European
Tryptophan	Amino acid	18	7804	European
Serine	Amino acid	3	7796	European
Pyroglutamine	Amino acid	5	7800	European
Proline	Amino acid	4	7816	European
Phenylalanine	Amino acid	33	115025	European
N-acetylglycine	Amino acid	7	7135	European
Leucine	Amino acid	34	115074	European
Kynurenine	Amino acid	6	7816	European
Isovalerylcarnitine	Amino acid	7	7789	European
Isoleucine	Amino acid	18	115075	European
Isobutyrylcarnitine	Amino acid	8	7812	European
Histidine	Amino acid	45	114895	European
Glycine	Amino acid	221	114972	European
Glutaroyl carnitine	Amino acid	11	7701	European
Glutamine	Amino acid	98	114750	European
Creatinine	Amino acid	91	110058	European
Citrulline	Amino acid	4	7773	European
Betaine	Amino acid	5	7806	European
Asparagine	Amino acid	3	7761	European
Alpha-hydroxyisovalerate	Amino acid	3	7668	European

Alanine	Amino acid	52	115074	European
4-acetamidobutanoate	Amino acid	6	6930	European
3-methyl-2-oxovalerate	Amino acid	3	7779	European
Pyruvate	Carbohydrate	60	114748	European
Mannose	Carbohydrate	6	7793	European
Lactate	Carbohydrate	16	114802	European
Glucose	Carbohydrate	38	114867	European
Erythronate	Carbohydrate	3	7752	European
1,5-anhydroglucitol (1,5-AG)	Carbohydrate	6	7746	European
Biliverdin	Cofactors and vitamins	9	6686	European
Bilirubin (Z,Z)	Cofactors and vitamins	8	6812	European
Bilirubin (E,Z or Z,E)	Cofactors and vitamins	4	5295	European
Bilirubin (E,E)	Cofactors and vitamins	7	7748	European
Acetate	Cofactors and vitamins	20	115046	European
Succinylcarnitine	Energy	10	6948	European
Citrate	Energy	80	115064	European
Acetone	Energy	19	115075	European
VLDL cholesterol	Lipid	188	115078	European
Total triglycerides	Lipid	239	115078	European
Total free cholesterol	Lipid	170	115078	European
Total fatty acids	Lipid	195	114999	European
Total esterified cholesterol	Lipid	169	115078	European
Total cholines	Lipid	181	114999	European
Total cholesterol	Lipid	165	115078	European
Tetradecanedioate	Lipid	4	6046	European

Sphingomyelins	Lipid	189	114999	European
Saturated fatty acids	Lipid	158	114999	European
Propionylcarnitine	Lipid	5	7813	European
Polyunsaturated fatty acids	Lipid	222	114999	European
Phosphoglycerides	Lipid	176	114999	European
Phosphatidylcholines	Lipid	191	114999	European
Octanoylcarnitine	Lipid	7	7790	European
Octadecanedioate	Lipid	4	7300	European
Monounsaturated fatty acids	Lipid	212	114999	European
Linoleic acid	Lipid	179	114999	European
LDL cholesterol	Lipid	153	115078	European
Hexanoylcarnitine	Lipid	9	7786	European
Hexadecanedioate	Lipid	5	6887	European
HDL cholesterol	Lipid	280	115078	European
Glycoprotein acetyls	Lipid	171	115078	European
Epiandrosterone sulfate	Lipid	7	7769	European
Docosahexaenoic acid	Lipid	161	114999	European
Dihomo-linolenate (20:3n3 or n6)	Lipid	3	7805	European
Decanoylcarnitine	Lipid	5	7766	European
Cis-4-decenoyl carnitine	Lipid	5	7660	European
Carnitine	Lipid	21	7797	European
Butyrylcarnitine	Lipid	25	7796	European
Arachidonate (20:4n6)	Lipid	5	7816	European
Apolipoprotein B	Lipid	167	115078	European
Apolipoprotein A1	Lipid	239	115078	European

Androsterone sulfate	Lipid	8	7785	European
Acetoacetate	Lipid	9	115075	European
5alpha-androstan-3beta,17beta-diol disulfate	Lipid	6	7345	European
4-androsten-3beta,17beta-diol disulfate 1	Lipid	6	7804	European
3-Hydroxybutyrate	Lipid	25	113595	European
3-dehydrocarnitine	Lipid	6	7809	European
2-hydroxyisobutyrate	Lipid	4	6539	European
22:6, docosahexaenoic acid	Lipid	6	13499	European
1-arachidonoylglycerophosphoinositol	Lipid	5	7797	European
1-arachidonoylglycerophosphoethanolamine	Lipid	4	7798	European
1-arachidonoylglycerophosphocholine	Lipid	5	7507	European
10-undecenoate (11:1n1)	Lipid	4	7806	European
Uridine	Nucleotide	3	7800	European
Urate	Nucleotide	5	7819	European
N-acetylornithine	Peptide	10	7574	European
HWESASXX	Peptide	3	7700	European
Glycoproteins	Peptide	82	18734	European
Gamma-glutamyltyrosine	Peptide	5	7468	European
Bradykinin, des-arg(9)	Peptide	5	4570	European
Albumin	Peptide	48	115060	European
X-18601	Unknown	3	7663	European
X-14626	Unknown	3	6904	European
X-14205	Unknown	3	1789	European
X-13435	Unknown	3	6970	European
X-13431	Unknown	7	6591	European

X-13429	Unknown	4	6344	European
X-12850	Unknown	3	6251	European
X-12844	Unknown	4	7768	European
X-12798	Unknown	13	7552	European
X-12728	Unknown	8	537	European
X-12696	Unknown	5	7409	European
X-12644	Unknown	3	7795	European
X-12556	Unknown	4	7483	European
X-12510	Unknown	7	7566	European
X-12456	Unknown	3	4774	European
X-12244	Unknown	6	6608	European
X-12093	Unknown	5	2854	European
X-12092	Unknown	19	7500	European
X-12063	Unknown	15	7197	European
X-11905	Unknown	3	4761	European
X-11793	Unknown	10	7611	European
X-11792	Unknown	3	2442	European
X-11787	Unknown	8	7811	European
X-11593	Unknown	13	7788	European
X-11538	Unknown	8	7804	European
X-11530	Unknown	8	7409	European
X-11529	Unknown	11	6664	European
X-11491	Unknown	4	6584	European
X-11469	Unknown	5	7779	European
X-11445	Unknown	3	2570	European

X-11444	Unknown	5	7758	European
X-11442	Unknown	7	7142	European
X-11441	Unknown	6	7072	European
X-11440	Unknown	6	7686	European
X-11315	Unknown	3	7785	European
X-11261	Unknown	6	7771	European
X-11204	Unknown	3	7799	European
X-10510	Unknown	3	7792	European
X-09789	Unknown	3	7805	European
X-08988	Unknown	3	7776	European
X-08402	Unknown	6	7726	European
X-03094	Unknown	5	7804	European
X-03056	Unknown	8	7812	European
X-02269	Unknown	4	7701	European
