# Supplementary material for: Association between human blood metabolome and the risk of gastrointestinal tumors
Source: PLoS One. 2024 May 30;19(5):e0304574. doi: 10.1371/journal.pone.0304574 (PMC11139295; doi:10.1371/journal.pone.0304574)
Supplement: S3 Table — (PDF) [file pone.0304574.s003.pdf]

Supplementary Table 3A. MR results in Liver Cancer.

Category	Exposures	No.of SNPs	Inverse variance weighting			MR-Egger			Weighted Median		
			OR/Beta	95% CI	*P*-val	OR/Beta	95% CI	*P*-val	OR/Beta	95% CI	*P*-val
**Amino acid**											
	Tryptophan	18	1.007	(0.995,1.020)	0.254	1.085	(0.914,1.289)	0.365	1.007	(0.991,1.024)	0.384
	4-acetamidobutanoate	6	1.002	(0.995,1.009)	0.648	1.001	(0.984,1.019)	0.908	1.000	(0.992,1.008)	1.000
	Proline	4	0.998	(0.991,1.005)	0.586	0.997	(0.982,1.011)	0.685	0.997	(0.990,1.004)	0.422
	Citrulline	4	1.003	(0.989,1.018)	0.637	1.050	(0.791,1.394)	0.769	1.009	(0.992,1.026)	0.312
	Betaine	5	0.998	(0.991,1.005)	0.597	1.001	(0.967,1.037)	0.946	0.998	(0.989,1.006)	0.562
	Kynurenine	6	1.001	(0.994,1.007)	0.845	1.001	(0.989,1.014)	0.856	0.998	(0.989,1.007)	0.641
	3-methyl-2-oxovalerate	3	0.990	(0.976,1.004)	0.156	0.986	(0.895,1.086)	0.823	0.990	(0.975,1.005)	0.202
	N-acetylglycine	7	1.002	(0.999,1.004)	0.240	0.999	(0.993,1.004)	0.683	1.002	(0.999,1.005)	0.265
	Serine	3	0.999	(0.986,1.011)	0.829	0.988	(0.810,1.205)	0.923	0.997	(0.986,1.008)	0.607
	Pyroglutamine	5	0.998	(0.994,1.002)	0.401	0.999	(0.987,1.011)	0.889	1.000	(0.995,1.004)	0.840
	Isobutyrylcarnitine	8	1.000	(0.997,1.002)	0.767	1.004	(0.997,1.012)	0.323	1.001	(0.997,1.004)	0.744
	Alpha-hydroxyisovalerate	3	0.998	(0.992,1.003)	0.401	0.998	(0.980,1.017)	0.891	0.998	(0.992,1.004)	0.444
	Asparagine	3	1.000	(0.991,1.010)	0.967	0.998	(0.976,1.021)	0.905	1.000	(0.990,1.010)	0.958
	Isovalerylcarnitine	7	1.011	(1.007,1.016)	1.81E-07	1.007	(0.980,1.034)	0.652	1.012	(1.007,1.018)	0.000
	Glutaroyl carnitine	11	0.999	(0.995,1.003)	0.590	0.993	(0.978,1.007)	0.338	0.998	(0.993,1.003)	0.382
	Tryptophan betaine	4	0.998	(0.995,1.001)	0.152	1.004	(0.998,1.011)	0.330	0.999	(0.996,1.001)	0.332
	Alanine	52	1.245	(0.620,2.500)	0.538	3.356	(0.538,20.915)	0.201	0.797	(0.298,2.134)	0.652
	Creatinine	91	0.394	(0.195,0.796)	0.009	0.075	(0.010,0.575)	0.015	0.201	(0.077,0.526)	0.001
	Glutamine	98	1.095	(0.678,1.769)	0.710	1.535	(0.706,3.338)	0.282	1.293	(0.616,2.713)	0.498
	Glycine	221	0.854	(0.704,1.037)	0.111	1.131	(0.880,1.455)	0.338	0.992	(0.729,1.348)	0.957
	Histidine	45	0.681	(0.336,1.378)	0.285	2.508	(0.619,10.157)	0.204	0.820	(0.275,2.447)	0.721

	Isoleucine	18	0.953	(0.321,2.828)	0.931	0.842	(0.061,11.539)	0.899	0.563	(0.136,2.330)	0.428
	Leucine	34	1.286	(0.571,2.899)	0.543	0.762	(0.161,3.608)	0.734	0.604	(0.189,1.924)	0.393
	Phenylalanine	33	1.255	(0.565,2.788)	0.577	1.583	(0.312,8.020)	0.583	1.464	(0.437,4.900)	0.537
	Tyrosine	76	0.999	(0.623,1.604)	0.997	0.998	(0.451,2.206)	0.995	1.246	(0.615,2.525)	0.542
	Valine	49	1.220	(0.624,2.384)	0.561	0.730	(0.199,2.678)	0.638	0.721	(0.269,1.934)	0.516
**Carbohydrate**											
	Mannose	6	0.995	(0.988,1.002)	0.149	1.007	(0.991,1.023)	0.429	0.995	(0.989,1.001)	0.084
	1,5-anhydroglucitol (1,5-AG)	6	1.000	(0.995,1.004)	0.905	0.997	(0.982,1.012)	0.698	1.001	(0.995,1.006)	0.843
	Erythronate	3	0.997	(0.982,1.013)	0.736	1.028	(0.897,1.178)	0.760	0.994	(0.976,1.013)	0.518
	Glucose	38	1.212	(0.550,2.669)	0.633	1.988	(0.319,12.376)	0.466	1.376	(0.384,4.932)	0.625
	Lactate	16	0.863	(0.233,3.200)	0.826	1.649	(0.012,229.757)	0.846	0.726	(0.139,3.790)	0.704
	Pyruvate	60	1.809	(1.014,3.227)	0.045	1.245	(0.370,4.189)	0.725	1.161	(0.496,2.717)	0.731
**Cofactors and vitamins**											
	Biliverdin	9	1.001	(0.999,1.003)	0.205	0.998	(0.995,1.002)	0.457	1.000	(0.998,1.003)	0.796
	Bilirubin (Z,Z)	8	1.001	(0.999,1.002)	0.452	0.999	(0.997,1.002)	0.714	1.000	(0.999,1.002)	0.891
	Bilirubin (E,E)	7	1.001	(0.999,1.003)	0.409	0.999	(0.996,1.003)	0.735	1.000	(0.998,1.002)	0.802
	Bilirubin (E,Z or Z,E)	4	1.001	(0.998,1.004)	0.500	0.998	(0.990,1.006)	0.640	1.000	(0.997,1.003)	0.945
	Acetate	20	1.491	(0.391,5.688)	0.558	3.916	(0.102,150.637)	0.473	1.939	(0.320,11.753)	0.471
**Energy**											
	Succinylcarnitine	10	1.000	(0.995,1.006)	0.861	0.999	(0.987,1.010)	0.843	1.001	(0.994,1.008)	0.747
	Acetone	19	1.176	(0.354,3.911)	0.791	0.953	(0.066,13.771)	0.973	1.362	(0.256,7.259)	0.717
	Citrate	80	1.063	(0.671,1.686)	0.794	1.381	(0.585,3.262)	0.464	1.620	(0.810,3.238)	0.172
**Lipid**											
	Arachidonate (20:4n6)	5	0.997	(0.992,1.001)	0.188	0.997	(0.982,1.011)	0.680	0.997	(0.992,1.002)	0.230
	Carnitine	21	1.002	(0.994,1.01)	0.615	1.010	(0.993,1.027)	0.248	1.003	(0.991,1.014)	0.630

	2-hydroxyisobutyrate	4	1.002	(0.995,1.009)	0.531	1.002	(0.981,1.024)	0.871	1.003	(0.995,1.012)	0.445
	Androsterone sulfate	8	1.001	(1.000,1.002)	0.112	1.000	(0.999,1.002)	0.555	1.001	(1.000,1.002)	0.038
	Hexanoylcarnitine	9	1.001	(0.996,1.006)	0.640	0.997	(0.986,1.007)	0.547	1.001	(0.997,1.005)	0.644
	Butyrylcarnitine	25	0.999	(0.998,1.000)	0.192	0.997	(0.994,0.999)	0.029	0.998	(0.997,1.000)	0.036
	Propionylcarnitine	5	1.010	(1.001,1.019)	0.029	0.999	(0.977,1.021)	0.945	1.005	(0.994,1.017)	0.343
	10-undecenoate (11:1n1)	4	0.998	(0.993,1.003)	0.462	1.002	(0.991,1.013)	0.723	1.000	(0.995,1.005)	0.990
	3-dehydrocarnitine	6	1.008	(0.999,1.016)	0.069	1.027	(0.995,1.061)	0.175	1.003	(0.994,1.012)	0.575
	1-arachidonoylglycerophosphocholine	5	0.996	(0.992,1.000)	0.073	0.999	(0.990,1.007)	0.791	0.997	(0.992,1.001)	0.144
	Octanoylcarnitine	7	1.000	(0.996,1.003)	0.795	1.001	(0.991,1.012)	0.790	1.001	(0.998,1.005)	0.522
	Decanoylcarnitine	5	0.999	(0.996,1.003)	0.620	1.001	(0.991,1.010)	0.898	1.000	(0.995,1.004)	0.877
	Epiandrosterone sulfate	7	1.001	(0.999,1.003)	0.199	1.000	(0.998,1.003)	0.781	1.001	(1.000,1.003)	0.098
	1-arachidonoylglycerophosphoinositol	5	0.996	(0.99,1.002)	0.210	0.985	(0.965,1.005)	0.244	0.994	(0.988,1.001)	0.075
	1-arachidonoylglycerophosphoethanolamine	4	0.996	(0.991,1.002)	0.246	0.988	(0.970,1.006)	0.321	0.996	(0.989,1.003)	0.230
	Tetradecanedioate	4	0.998	(0.995,1.000)	0.099	0.997	(0.991,1.003)	0.429	0.998	(0.995,1.001)	0.136
	Hexadecanedioate	5	0.997	(0.994,0.999)	0.015	0.996	(0.989,1.004)	0.383	0.997	(0.994,1.000)	0.097
	Dihomo-linolenate (20:3n3 or n6)	3	1.006	(0.988,1.024)	0.546	0.985	(0.923,1.051)	0.728	1.000	(0.989,1.011)	0.979
	Octadecanedioate	4	0.992	(0.987,0.998)	0.006	0.995	(0.965,1.027)	0.795	0.992	(0.986,0.999)	0.026
	5alpha-androstan-3beta,17beta-diol disulfate	6	1.001	(0.999,1.002)	0.197	1.002	(0.999,1.004)	0.294	1.002	(1.000,1.003)	0.107
	4-androsten-3beta,17beta-diol disulfate 1	6	1.002	(1.000,1.003)	0.051	1.001	(0.998,1.004)	0.511	1.001	(0.999,1.003)	0.219
	Cis-4-decenoyl carnitine	5	0.998	(0.993,1.003)	0.472	1.003	(0.990,1.017)	0.668	0.999	(0.995,1.004)	0.801
	22:6, docosahexaenoic acid	6	0.256	(0.052,1.273)	0.096	0.001	(0.000,0.105)	0.046	0.551	(0.200,1.519)	0.249
	Acetoacetate	9	0.266	(0.052,1.356)	0.111	0.208	(0.001,31.287)	0.558	0.579	(0.071,4.714)	0.609
	Apolipoprotein A1	239	1.138	(0.828,1.564)	0.425	1.037	(0.573,1.877)	0.904	1.079	(0.638,1.822)	0.777
	Apolipoprotein B	167	0.536	(0.398,0.722)	4.21E-05	0.324	(0.196,0.536)	0.000	0.545	(0.343,0.866)	0.010
	3-Hydroxybutyrate	25	0.495	(0.154,1.590)	0.237	0.268	(0.012,6.189)	0.420	0.662	(0.135,3.235)	0.610

	Total cholines	181	0.908	(0.601,1.370)	0.645	1.100	(0.490,2.466)	0.818	1.263	(0.692,2.302)	0.447
	Docosahexaenoic acid	161	0.579	(0.410,0.816)	0.002	0.432	(0.251,0.744)	0.003	0.762	(0.467,1.244)	0.277
	Glycoprotein acetyls	171	0.754	(0.535,1.065)	0.109	0.800	(0.422,1.516)	0.495	0.779	(0.456,1.333)	0.362
	HDL cholesterol	280	0.964	(0.739,1.257)	0.786	0.921	(0.576,1.471)	0.730	0.928	(0.592,1.455)	0.745
	Linoleic acid	179	0.727	(0.480,1.101)	0.132	0.514	(0.218,1.212)	0.130	1.082	(0.622,1.884)	0.780
	LDL cholesterol	153	0.481	(0.353,0.657)	4.02E-06	0.293	(0.177,0.486)	0.000	0.391	(0.245,0.624)	0.000
	Monounsaturated fatty acids	212	0.762	(0.525,1.105)	0.152	1.058	(0.531,2.111)	0.873	0.854	(0.489,1.494)	0.581
	Phosphatidylcholines	191	0.862	(0.584,1.273)	0.455	0.986	(0.472,2.061)	0.970	1.146	(0.665,1.974)	0.624
	Phosphoglycerides	176	0.942	(0.626,1.417)	0.774	0.950	(0.433,2.087)	0.899	1.256	(0.691,2.281)	0.455
	Polyunsaturated fatty acids	222	0.585	(0.401,0.853)	0.005	0.455	(0.216,0.958)	0.039	0.908	(0.551,1.498)	0.706
	Saturated fatty acids	158	0.858	(0.539,1.365)	0.518	0.727	(0.289,1.827)	0.499	0.949	(0.501,1.800)	0.874
	Sphingomyelins	189	0.494	(0.347,0.703)	8.89E-05	0.305	(0.164,0.567)	0.000	0.578	(0.334,1.002)	0.051
	Total cholesterol	165	0.458	(0.317,0.663)	3.42E-05	0.198	(0.105,0.373)	0.000	0.348	(0.201,0.602)	0.000
	Total esterified cholesterol	169	0.448	(0.309,0.650)	2.29E-05	0.191	(0.100,0.362)	0.000	0.340	(0.196,0.589)	0.000
	Total fatty acids	195	0.787	(0.534,1.160)	0.227	0.723	(0.346,1.514)	0.391	0.865	(0.488,1.535)	0.621
	Total free cholesterol	170	0.508	(0.349,0.739)	4.00E-04	0.219	(0.115,0.416)	0.000	0.635	(0.366,1.100)	0.105
	Total triglycerides	239	0.928	(0.656,1.312)	0.671	1.122	(0.611,2.060)	0.710	0.869	(0.512,1.475)	0.604
	VLDL cholesterol	188	0.702	(0.479,1.028)	0.069	0.512	(0.239,1.096)	0.087	0.960	(0.580,1.589)	0.874
**Nucleotide**											
	Uridine	3	1.017	(1.000,1.034)	0.051	1.064	(0.981,1.155)	0.376	1.015	(0.994,1.036)	0.168
	Urate	5	0.996	(0.988,1.004)	0.342	0.995	(0.976,1.014)	0.630	0.995	(0.987,1.004)	0.315
**Peptide**											
	Gamma-glutamyltyrosine	5	0.995	(0.982,1.008)	0.428	0.931	(0.833,1.041)	0.300	0.996	(0.980,1.012)	0.637
	N-acetylornithine	10	1.000	(0.999,1.002)	0.530	1.002	(1.000,1.005)	0.107	1.000	(0.999,1.002)	0.654
	HWESASXX	3	1.001	(0.997,1.006)	0.597	1.005	(0.983,1.028)	0.723	1.001	(0.996,1.007)	0.612

	Bradykinin, des-arg(9)	5	0.999	(0.998,1.000)	0.240	0.999	(0.995,1.002)	0.488	0.999	(0.998,1.001)	0.234
	Glycoproteins	82	0.833	(0.711,0.977)	0.024	1.143	(0.857,1.525)	0.367	0.920	(0.709,1.193)	0.528
	Albumin	48	2.926	(1.466,5.839)	0.002	6.945	(2.033,23.725)	0.003	4.762	(1.625,13.955)	0.004
**Unknown**											
	X-03094	5	1.000	(0.990,1.010)	0.948	1.018	(0.978,1.059)	0.448	0.999	(0.988,1.010)	0.879
	X-18601	3	1.000	(0.992,1.009)	0.921	1.024	(0.965,1.086)	0.581	1.004	(0.998,1.010)	0.158
	X-08402	6	0.999	(0.993,1.006)	0.835	0.994	(0.982,1.005)	0.333	0.998	(0.992,1.004)	0.486
	X-08988	3	1.003	(0.993,1.012)	0.597	0.989	(0.971,1.007)	0.441	1.002	(0.994,1.011)	0.629
	X-10510	3	1.001	(0.988,1.013)	0.937	0.986	(0.965,1.007)	0.419	0.999	(0.991,1.008)	0.894
	X-11204	3	1.013	(0.996,1.030)	0.123	0.752	(0.368,1.540)	0.579	1.008	(0.989,1.028)	0.420
	X-02269	4	1.002	(0.997,1.007)	0.472	1.011	(0.974,1.050)	0.621	1.003	(0.999,1.007)	0.140
	X-11261	6	1.002	(0.998,1.006)	0.338	0.999	(0.985,1.013)	0.883	1.001	(0.996,1.006)	0.704
	X-11315	3	1.005	(0.996,1.015)	0.251	1.025	(0.988,1.064)	0.417	1.006	(0.995,1.018)	0.284
	X-03056	8	1.000	(0.995,1.004)	0.969	0.999	(0.987,1.011)	0.910	0.999	(0.993,1.005)	0.731
	X-09789	3	1.001	(0.997,1.005)	0.626	1.008	(0.985,1.032)	0.608	1.002	(0.997,1.007)	0.526
	X-11440	6	1.002	(1.000,1.004)	0.076	1.001	(0.997,1.004)	0.694	1.001	(0.999,1.003)	0.289
	X-11441	6	1.001	(0.999,1.003)	0.443	0.999	(0.994,1.004)	0.646	1.000	(0.998,1.003)	0.872
	X-11442	7	1.001	(0.999,1.003)	0.390	0.999	(0.994,1.003)	0.526	1.000	(0.998,1.003)	0.862
	X-11444	5	1.006	(0.999,1.013)	0.080	0.996	(0.971,1.023)	0.800	1.003	(0.996,1.009)	0.467
	X-11445	3	0.997	(0.993,1.002)	0.220	0.987	(0.921,1.056)	0.766	0.996	(0.993,1.000)	0.059
	X-11469	5	1.003	(0.998,1.007)	0.205	0.993	(0.955,1.033)	0.755	1.004	(1.000,1.008)	0.069
	X-11491	4	0.997	(0.994,1.000)	0.090	0.999	(0.986,1.012)	0.876	0.997	(0.994,1.001)	0.130
	X-11529	11	0.999	(0.999,1.000)	0.222	1.000	(0.998,1.001)	0.898	0.999	(0.998,1.000)	0.157
	X-11530	8	1.001	(0.999,1.003)	0.466	0.999	(0.995,1.003)	0.713	1.000	(0.998,1.002)	0.872
	X-11538	8	0.997	(0.995,0.999)	0.005	0.998	(0.994,1.003)	0.495	0.998	(0.995,1.000)	0.078

	X-11593--O-methylascorbate	13	0.996	(0.992,1.000)	0.027	0.996	(0.989,1.004)	0.371	0.996	(0.991,1.001)	0.119
	X-11787	8	1.006	(0.999,1.014)	0.104	1.009	(0.993,1.025)	0.306	1.007	(0.998,1.016)	0.142
	X-11792	3	0.999	(0.997,1.001)	0.466	0.997	(0.990,1.005)	0.633	0.999	(0.997,1.001)	0.407
	X-11793--oxidized bilirubin	10	1.002	(0.999,1.004)	0.165	0.999	(0.994,1.003)	0.629	1.000	(0.997,1.003)	0.845
	X-11905	3	0.997	(0.994,1.001)	0.112	0.998	(0.990,1.007)	0.792	0.997	(0.994,1.001)	0.124
	X-12063	15	1.000	(0.998,1.001)	0.590	1.001	(0.999,1.004)	0.386	1.000	(0.998,1.002)	0.955
	X-12092	19	1.000	(0.999,1.001)	0.672	1.000	(0.999,1.002)	0.485	1.000	(0.999,1.001)	0.837
	X-12093	5	1.000	(0.998,1.002)	0.948	1.001	(0.995,1.006)	0.853	1.000	(0.998,1.002)	0.938
	X-12244--N-acetylcarnosine	6	1.002	(0.996,1.009)	0.467	1.009	(0.967,1.053)	0.699	1.001	(0.994,1.009)	0.736
	X-12456	3	0.998	(0.995,1.002)	0.297	0.992	(0.979,1.004)	0.420	0.998	(0.994,1.001)	0.211
	X-12510--2-aminooctanoic acid	7	1.000	(0.997,1.002)	0.845	1.002	(0.998,1.006)	0.452	1.000	(0.997,1.003)	0.947
	X-12556	4	1.005	(0.997,1.013)	0.205	1.031	(0.959,1.108)	0.498	1.005	(0.996,1.014)	0.316
	X-12644	3	0.996	(0.981,1.012)	0.647	0.985	(0.841,1.154)	0.882	0.997	(0.984,1.011)	0.713
	X-12696	5	1.000	(0.995,1.006)	0.871	0.995	(0.973,1.017)	0.664	1.001	(0.994,1.007)	0.834
	X-12728	8	1.000	(1.000,1.000)	0.713	1.000	(0.999,1.001)	0.690	1.000	(1.000,1.000)	0.758
	X-12798	13	1.000	(0.997,1.002)	0.693	1.000	(0.996,1.003)	0.924	1.000	(0.997,1.003)	0.972
	X-12844	4	1.008	(1.000,1.015)	0.037	1.018	(0.972,1.068)	0.528	1.008	(1.000,1.017)	0.062
	X-12850	3	1.002	(0.999,1.006)	0.165	1.005	(0.997,1.013)	0.415	1.003	(0.999,1.006)	0.174
	X-13429	4	0.999	(0.997,1.000)	0.109	0.997	(0.993,1.002)	0.368	0.998	(0.996,1.000)	0.084
	X-13431--nonanoylcarnitine	7	0.998	(0.996,1.001)	0.140	1.000	(0.995,1.005)	0.909	0.999	(0.996,1.001)	0.341
	X-13435	3	0.998	(0.992,1.005)	0.620	1.005	(0.980,1.031)	0.760	0.997	(0.99,1.004)	0.456
	X-14205--alpha-glutamyltyrosine	3	1.000	(0.996,1.005)	0.936	0.996	(0.984,1.008)	0.627	1.000	(0.995,1.005)	0.982
	X-14626	3	0.996	(0.991,1.001)	0.120	0.992	(0.981,1.004)	0.405	0.995	(0.990,1.001)	0.078

Supplementary Table 3B. MR results in Colorectal Cancer.

Category	Exposures	No.of SNPs	Inverse variance weighting			MR-Egger			Weighted Median		
			OR/Beta	95% CI	*P*-val	OR/Beta	95% CI	*P*-val	OR/Beta	95% CI	*P*-val
**Amino acid**											
	Tryptophan	18	0.967	(0.928,1.007)	0.106	1.144	(0.656,1.996)	0.642	0.984	(0.928,1.042)	0.578
	4-acetamidobutanoate	6	1.021	(0.994,1.05)	0.131	1.063	(0.999,1.132)	0.127	1.029	(1.001,1.058)	0.044
	Proline	4	1.008	(0.986,1.03)	0.486	0.993	(0.944,1.043)	0.797	1.007	(0.981,1.033)	0.592
	Citrulline	4	0.996	(0.95,1.044)	0.868	1.665	(0.744,3.728)	0.341	0.987	(0.934,1.043)	0.643
	Betaine	5	1.007	(0.985,1.03)	0.54	1.047	(0.935,1.172)	0.488	0.991	(0.964,1.019)	0.513
	Kynurenine	6	0.982	(0.954,1.012)	0.241	0.99	(0.932,1.052)	0.764	0.98	(0.949,1.011)	0.204
	3-methyl-2-oxovalerate	3	0.973	(0.906,1.046)	0.457	1.375	(1.004,1.881)	0.297	0.983	(0.929,1.04)	0.55
	N-acetylglycine	7	1.003	(0.994,1.012)	0.516	1.006	(0.984,1.028)	0.631	1.003	(0.993,1.012)	0.573
	Serine	3	1.016	(0.981,1.051)	0.378	0.989	(0.572,1.709)	0.974	1.015	(0.98,1.052)	0.397
	Pyroglutamine	5	1.005	(0.992,1.019)	0.437	1.011	(0.972,1.052)	0.624	1.007	(0.991,1.024)	0.38
	Isobutyrylcarnitine	8	1.004	(0.995,1.014)	0.388	1.005	(0.98,1.03)	0.725	1.008	(0.997,1.02)	0.16
	Alpha-hydroxyisovalerate	3	0.99	(0.968,1.013)	0.398	1.038	(0.978,1.101)	0.434	0.99	(0.97,1.009)	0.299
	Asparagine	3	1.016	(0.985,1.049)	0.316	1.042	(0.95,1.144)	0.543	1.023	(0.99,1.057)	0.173
	Isovalerylcarnitine	7	0.998	(0.985,1.012)	0.827	1.01	(0.931,1.096)	0.821	0.998	(0.981,1.015)	0.778
	Glutaroyl carnitine	11	0.996	(0.983,1.008)	0.474	0.977	(0.932,1.024)	0.361	0.99	(0.974,1.006)	0.234
	Tryptophan betaine	4	0.999	(0.992,1.006)	0.799	0.993	(0.971,1.015)	0.586	1	(0.992,1.009)	0.933
	Alanine	52	0.887	(0.696,1.132)	0.335	0.591	(0.314,1.11)	0.108	0.888	(0.63,1.253)	0.499
	Creatinine	91	0.799	(0.63,1.012)	0.062	0.959	(0.478,1.923)	0.906	0.748	(0.536,1.045)	0.088
	Glutamine	98	1.182	(1.02,1.37)	0.026	1.104	(0.868,1.404)	0.422	1.115	(0.885,1.404)	0.357
	Glycine	221	1.022	(0.963,1.085)	0.466	1.027	(0.949,1.111)	0.513	1.003	(0.907,1.109)	0.954
	Histidine	45	1.011	(0.77,1.326)	0.939	1.069	(0.619,1.846)	0.812	1.004	(0.71,1.419)	0.982

	Isoleucine	18	1.109	(0.68,1.809)	0.678	2.615	(0.857,7.975)	0.111	0.895	(0.506,1.582)	0.703
	Leucine	34	1.025	(0.768,1.369)	0.866	1.23	(0.706,2.143)	0.471	1.162	(0.773,1.746)	0.47
	Phenylalanine	33	1.056	(0.812,1.374)	0.683	1.805	(1.059,3.075)	0.038	1.294	(0.884,1.895)	0.185
	Tyrosine	76	1.072	(0.929,1.238)	0.338	0.931	(0.733,1.182)	0.56	1.053	(0.842,1.317)	0.652
	Valine	49	0.986	(0.756,1.287)	0.919	1.122	(0.669,1.883)	0.665	1.097	(0.767,1.568)	0.613
**Carbohydrate**											
	Mannose	6	1.028	(1.01,1.046)	0.002	1.009	(0.963,1.057)	0.719	1.027	(1.008,1.046)	0.006
	1,5-anhydroglucitol (1,5-AG)	6	0.987	(0.973,1.001)	0.065	0.962	(0.916,1.011)	0.199	0.981	(0.964,0.999)	0.036
	Erythronate	3	1.007	(0.952,1.065)	0.815	0.786	(0.522,1.182)	0.453	1.021	(0.963,1.083)	0.478
	Glucose	38	0.933	(0.72,1.21)	0.603	0.674	(0.37,1.228)	0.206	0.804	(0.527,1.226)	0.31
	Lactate	16	0.831	(0.493,1.401)	0.488	1.58	(0.213,11.734)	0.661	0.762	(0.393,1.478)	0.421
	Pyruvate	60	1.06	(0.821,1.37)	0.654	1.048	(0.61,1.799)	0.867	1.235	(0.878,1.737)	0.225
**Cofactors and vitamins**											
	Biliverdin	9	0.996	(0.989,1.002)	0.196	0.992	(0.979,1.005)	0.259	0.995	(0.987,1.003)	0.195
	Bilirubin (Z,Z)	8	0.995	(0.991,1)	0.041	0.999	(0.99,1.008)	0.835	0.996	(0.991,1.001)	0.157
	Bilirubin (E,E)	7	0.995	(0.989,1.001)	0.131	0.995	(0.981,1.008)	0.455	0.995	(0.988,1.002)	0.159
	Bilirubin (E,Z or Z,E)	4	0.996	(0.983,1.008)	0.503	0.995	(0.959,1.033)	0.81	0.994	(0.983,1.004)	0.214
	Acetate	20	2.16	(1.253,3.723)	0.006	1.298	(0.293,5.756)	0.736	1.553	(0.848,2.845)	0.154
**Energy**											
	Succinylcarnitine	10	0.999	(0.981,1.017)	0.908	1.03	(0.992,1.069)	0.166	1.004	(0.982,1.026)	0.711
	Acetone	19	1.502	(0.948,2.379)	0.083	1.917	(0.677,5.428)	0.237	1.615	(0.884,2.951)	0.119
	Citrate	80	1.209	(1.02,1.433)	0.029	1.18	(0.858,1.623)	0.313	1.209	(0.95,1.539)	0.123
**Lipid**											
	Arachidonate (20:4n6)	5	1.026	(1.011,1.041)	0.001	1.023	(0.975,1.073)	0.426	1.026	(1.01,1.043)	0.002
	Carnitine	21	0.98	(0.952,1.009)	0.172	0.977	(0.92,1.037)	0.449	0.982	(0.945,1.02)	0.339

	2-hydroxyisobutyrate	4	0.983	(0.96,1.005)	0.131	1.006	(0.942,1.074)	0.878	0.983	(0.956,1.01)	0.209
	Androsterone sulfate	8	0.999	(0.996,1.002)	0.638	1	(0.997,1.004)	0.826	1	(0.996,1.003)	0.819
	Hexanoylcarnitine	9	1	(0.99,1.01)	0.96	1.006	(0.985,1.028)	0.572	1.002	(0.989,1.014)	0.813
	Butyrylcarnitine	25	1	(0.995,1.004)	0.875	1.005	(0.996,1.014)	0.271	0.998	(0.993,1.003)	0.377
	Propionylcarnitine	5	0.988	(0.953,1.023)	0.494	0.991	(0.893,1.099)	0.874	0.977	(0.941,1.014)	0.225
	10-undecenoate (11:1n1)	4	1	(0.98,1.019)	0.968	0.968	(0.934,1.004)	0.221	1.001	(0.983,1.019)	0.931
	3-dehydrocarnitine	6	0.991	(0.969,1.013)	0.426	0.958	(0.873,1.051)	0.418	0.995	(0.969,1.022)	0.725
	1-arachidonoylglycerophosphocholine	5	1.01	(0.992,1.029)	0.29	1.038	(1.01,1.067)	0.076	1.013	(0.998,1.028)	0.087
	Octanoylcarnitine	7	1.001	(0.993,1.01)	0.764	1.006	(0.98,1.031)	0.684	1.002	(0.991,1.013)	0.717
	Decanoylcarnitine	5	1.002	(0.991,1.014)	0.701	1.006	(0.975,1.038)	0.728	1.002	(0.99,1.015)	0.693
	Epiandrosterone sulfate	7	0.998	(0.994,1.002)	0.296	0.999	(0.993,1.005)	0.808	0.997	(0.991,1.002)	0.211
	1-arachidonoylglycerophosphoinositol	5	1.012	(0.983,1.042)	0.432	0.995	(0.886,1.117)	0.935	1.011	(0.989,1.033)	0.342
	1-arachidonoylglycerophosphoethanolamine	4	1.019	(0.999,1.039)	0.057	1.054	(0.993,1.119)	0.226	1.016	(0.994,1.039)	0.162
	Tetradecanedioate	4	1.004	(0.994,1.014)	0.418	1.012	(0.984,1.041)	0.495	1.005	(0.995,1.014)	0.316
	Hexadecanedioate	5	1.005	(0.996,1.014)	0.294	1.012	(0.983,1.041)	0.485	1.006	(0.996,1.016)	0.272
	Dihomo-linolenate (20:3n3 or n6)	3	0.977	(0.947,1.007)	0.124	0.968	(0.866,1.082)	0.666	0.973	(0.939,1.008)	0.131
	Octadecanedioate	4	1.009	(0.991,1.027)	0.31	1.006	(0.919,1.103)	0.904	1.008	(0.987,1.029)	0.476
	5alpha-androstan-3beta,17beta-diol disulfate	6	1	(0.995,1.005)	0.939	1	(0.992,1.008)	0.984	0.998	(0.992,1.004)	0.569
	4-androsten-3beta,17beta-diol disulfate 1	6	1	(0.994,1.005)	0.958	0.999	(0.989,1.01)	0.915	1.001	(0.995,1.007)	0.822
	Cis-4-decenoyl carnitine	5	1.002	(0.989,1.014)	0.791	1.006	(0.975,1.039)	0.72	1.002	(0.988,1.017)	0.784
	22:6, docosahexaenoic acid	6	1.119	(0.825,1.517)	0.471	1.618	(0.398,6.58)	0.538	1.024	(0.726,1.445)	0.892
	Acetoacetate	9	1.247	(0.684,2.274)	0.472	2.487	(0.377,16.393)	0.375	1.303	(0.614,2.764)	0.49
	Apolipoprotein A1	239	0.976	(0.876,1.088)	0.667	0.953	(0.778,1.168)	0.645	0.983	(0.823,1.173)	0.849
	Apolipoprotein B	167	1.03	(0.94,1.128)	0.529	0.946	(0.81,1.105)	0.484	1.02	(0.892,1.167)	0.775
	3-Hydroxybutyrate	25	1.331	(0.944,1.878)	0.103	3.815	(1.548,9.403)	0.008	1.181	(0.728,1.914)	0.501

	Total cholines	181	0.982	(0.867,1.113)	0.778	1.089	(0.852,1.39)	0.497	0.996	(0.83,1.196)	0.968
	Docosahexaenoic acid	161	1.164	(1.05,1.289)	0.004	1.296	(1.102,1.524)	0.002	1.298	(1.115,1.512)	0.001
	Glycoprotein acetyls	171	0.936	(0.838,1.045)	0.24	0.93	(0.757,1.142)	0.487	0.879	(0.744,1.038)	0.129
	HDL cholesterol	280	1	(0.913,1.096)	0.992	0.927	(0.79,1.089)	0.358	0.952	(0.818,1.109)	0.531
	Linoleic acid	179	0.93	(0.824,1.049)	0.236	0.882	(0.687,1.131)	0.322	0.868	(0.728,1.034)	0.113
	LDL cholesterol	153	1.029	(0.931,1.136)	0.58	0.981	(0.833,1.156)	0.818	1.018	(0.881,1.176)	0.812
	Monounsaturated fatty acids	212	0.9	(0.805,1.006)	0.063	0.84	(0.683,1.033)	0.1	0.89	(0.737,1.074)	0.223
	Phosphatidylcholines	191	0.947	(0.846,1.061)	0.347	1.02	(0.824,1.263)	0.857	0.992	(0.838,1.175)	0.928
	Phosphoglycerides	176	0.957	(0.844,1.086)	0.494	0.976	(0.765,1.244)	0.845	0.991	(0.823,1.193)	0.926
	Polyunsaturated fatty acids	222	1.025	(0.92,1.143)	0.651	1.061	(0.856,1.314)	0.591	0.989	(0.842,1.162)	0.893
	Saturated fatty acids	158	0.922	(0.814,1.045)	0.205	0.847	(0.661,1.085)	0.19	0.965	(0.793,1.175)	0.725
	Sphingomyelins	189	1.084	(0.972,1.209)	0.148	0.976	(0.806,1.182)	0.803	0.996	(0.831,1.194)	0.968
	Total cholesterol	165	1.033	(0.925,1.152)	0.567	1.026	(0.846,1.245)	0.792	1.021	(0.87,1.2)	0.795
	Total esterified cholesterol	169	1.025	(0.916,1.146)	0.669	1.011	(0.83,1.231)	0.917	1.016	(0.858,1.202)	0.855
	Total fatty acids	195	0.941	(0.84,1.053)	0.288	0.847	(0.684,1.049)	0.13	0.899	(0.752,1.075)	0.244
	Total free cholesterol	170	1.029	(0.926,1.145)	0.592	1.033	(0.858,1.245)	0.731	1.028	(0.877,1.204)	0.736
	Total triglycerides	240	0.925	(0.834,1.027)	0.144	0.928	(0.773,1.114)	0.423	0.905	(0.762,1.075)	0.255
	VLDL cholesterol	188	1.04	(0.941,1.151)	0.441	0.955	(0.781,1.168)	0.657	1.031	(0.881,1.205)	0.706
**Nucleotide**											
	Uridine	3	0.99	(0.937,1.045)	0.706	1.142	(0.877,1.487)	0.504	0.987	(0.929,1.05)	0.687
	Urate	5	0.982	(0.957,1.008)	0.179	1.007	(0.952,1.066)	0.812	0.989	(0.96,1.019)	0.467
**Peptide**											
	Gamma-glutamyltyrosine	5	0.985	(0.944,1.027)	0.467	1.124	(0.784,1.611)	0.57	0.993	(0.94,1.049)	0.793
	N-acetylornithine	10	0.998	(0.993,1.004)	0.608	1.002	(0.99,1.014)	0.753	0.999	(0.993,1.004)	0.629
	HWESASXX	3	0.997	(0.978,1.017)	0.796	1.019	(0.928,1.119)	0.758	0.999	(0.982,1.016)	0.922

	Bradykinin, des-arg(9)	5	0.994	(0.99,0.998)	0.003	0.985	(0.974,0.996)	0.081	0.993	(0.988,0.997)	0.002
	Glycoproteins	82	1.033	(0.98,1.089)	0.225	1.042	(0.947,1.146)	0.401	1.01	(0.92,1.108)	0.841
	Albumin	48	0.79	(0.598,1.044)	0.097	0.674	(0.412,1.103)	0.124	0.712	(0.478,1.06)	0.094
**Unknown**											
	X-03094	5	0.974	(0.948,1.002)	0.065	0.99	(0.876,1.119)	0.882	0.982	(0.949,1.017)	0.315
	X-18601	3	0.996	(0.981,1.01)	0.553	0.96	(0.879,1.049)	0.531	0.996	(0.979,1.013)	0.631
	X-08402	6	1.017	(1.001,1.033)	0.035	1.011	(0.983,1.039)	0.495	1.016	(0.999,1.034)	0.072
	X-08988	3	1.003	(0.976,1.03)	0.841	1.026	(0.967,1.088)	0.552	1.007	(0.979,1.035)	0.641
	X-10510	3	1.022	(0.997,1.049)	0.087	1.027	(0.971,1.087)	0.522	1.024	(0.997,1.053)	0.083
	X-11204	3	0.989	(0.938,1.043)	0.684	0.755	(0.074,7.687)	0.852	0.993	(0.933,1.058)	0.836
	X-02269	4	0.996	(0.986,1.007)	0.497	1.024	(0.96,1.092)	0.542	0.998	(0.987,1.01)	0.775
	X-11261	6	1.004	(0.99,1.017)	0.61	1.003	(0.955,1.053)	0.92	1.008	(0.992,1.024)	0.352
	X-11315	3	0.969	(0.92,1.02)	0.234	0.843	(0.75,0.948)	0.215	0.988	(0.952,1.025)	0.521
	X-03056	8	1.001	(0.987,1.016)	0.887	1.001	(0.962,1.041)	0.972	0.998	(0.98,1.017)	0.863
	X-09789	3	1.007	(0.99,1.024)	0.427	0.986	(0.877,1.109)	0.854	1.001	(0.984,1.019)	0.88
	X-11440	6	1.001	(0.995,1.008)	0.694	0.997	(0.987,1.008)	0.635	1.001	(0.994,1.008)	0.841
	X-11441	6	0.995	(0.988,1.002)	0.167	0.993	(0.978,1.009)	0.432	0.995	(0.987,1.002)	0.169
	X-11442	7	0.995	(0.988,1.002)	0.135	0.996	(0.983,1.01)	0.613	0.995	(0.987,1.003)	0.182
	X-11444	5	1.006	(0.989,1.023)	0.468	1.005	(0.948,1.066)	0.877	1.004	(0.983,1.025)	0.717
	X-11445	3	1.001	(0.991,1.01)	0.86	0.999	(0.892,1.12)	0.992	1.001	(0.99,1.011)	0.908
	X-11469	5	0.998	(0.988,1.008)	0.695	1.007	(0.927,1.095)	0.874	0.999	(0.987,1.011)	0.818
	X-11491	4	1.004	(0.995,1.014)	0.38	0.997	(0.957,1.038)	0.896	1.002	(0.991,1.013)	0.746
	X-11529	11	1.001	(0.998,1.004)	0.695	1.002	(0.997,1.007)	0.53	1	(0.997,1.004)	0.788
	X-11530	8	0.994	(0.988,1.001)	0.084	0.998	(0.985,1.011)	0.765	0.995	(0.987,1.002)	0.169
	X-11538	8	1.004	(0.997,1.01)	0.281	1.003	(0.99,1.016)	0.682	1.005	(0.997,1.013)	0.237

	X-11593--O-methylascorbate	13	1.012	(0.999,1.025)	0.07	1.017	(0.992,1.043)	0.221	1.014	(0.997,1.03)	0.1
	X-11787	8	0.995	(0.965,1.025)	0.734	0.964	(0.906,1.025)	0.284	0.98	(0.95,1.012)	0.22
	X-11792	3	0.992	(0.984,0.999)	0.022	0.974	(0.95,0.998)	0.279	0.99	(0.982,0.997)	0.006
	X-11793--oxidized bilirubin	10	0.995	(0.987,1.003)	0.195	0.991	(0.976,1.006)	0.291	0.994	(0.985,1.003)	0.187
	X-11905	3	1.003	(0.992,1.015)	0.548	0.99	(0.962,1.018)	0.6	1.002	(0.99,1.014)	0.73
	X-12063	15	0.997	(0.993,1.001)	0.178	0.997	(0.989,1.005)	0.434	0.997	(0.991,1.003)	0.293
	X-12092	19	0.998	(0.995,1)	0.066	0.998	(0.994,1.001)	0.243	0.997	(0.993,1)	0.057
	X-12093	5	0.996	(0.99,1.003)	0.261	0.995	(0.977,1.013)	0.611	0.997	(0.989,1.004)	0.384
	X-12244--N-acetylcarnosine	6	0.992	(0.972,1.013)	0.457	1.055	(0.919,1.212)	0.488	0.989	(0.965,1.015)	0.414
	X-12456	3	1.001	(0.99,1.013)	0.818	1.003	(0.963,1.045)	0.911	1.001	(0.989,1.013)	0.837
	X-12510--2-aminooctanoic acid	7	0.992	(0.984,1.001)	0.072	0.998	(0.982,1.013)	0.762	0.991	(0.982,1)	0.051
	X-12556	4	1.037	(1.01,1.063)	0.006	1.059	(0.813,1.38)	0.712	1.037	(1.005,1.071)	0.024
	X-12644	3	0.996	(0.967,1.026)	0.792	0.971	(0.783,1.204)	0.832	1	(0.966,1.035)	0.985
	X-12696	5	0.981	(0.963,0.999)	0.039	0.956	(0.891,1.026)	0.299	0.975	(0.954,0.996)	0.019
	X-12728	8	1	(0.999,1.002)	0.666	1.005	(1.001,1.009)	0.062	1.001	(0.999,1.002)	0.361
	X-12798	13	1.002	(0.995,1.009)	0.575	0.999	(0.988,1.01)	0.829	0.999	(0.99,1.008)	0.864
	X-12844	4	0.997	(0.974,1.02)	0.781	1.058	(0.909,1.232)	0.542	0.996	(0.969,1.024)	0.784
	X-12850	3	1	(0.99,1.011)	0.932	0.999	(0.974,1.025)	0.967	1	(0.989,1.011)	0.99
	X-13429	4	1.003	(0.997,1.009)	0.36	1.002	(0.988,1.016)	0.804	1.001	(0.995,1.008)	0.658
	X-13431--nonanoylcarnitine	7	0.998	(0.99,1.006)	0.541	1.002	(0.981,1.023)	0.86	0.997	(0.989,1.004)	0.382
	X-13435	3	1.01	(0.976,1.045)	0.577	1.019	(0.855,1.214)	0.869	1.01	(0.985,1.035)	0.438
	X-14205--alpha-glutamyltyrosine	3	1.004	(0.99,1.018)	0.569	1.007	(0.972,1.043)	0.771	1.005	(0.989,1.022)	0.509
	X-14626	3	1.004	(0.987,1.02)	0.67	1.004	(0.968,1.042)	0.864	1.003	(0.987,1.021)	0.691

Supplementary Table 3C. MR results in Esophageal Cancer.

Category	Exposures	No.of SNPs	Inverse variance weighting			MR-Egger			Weighted Median		
			OR/Beta	95% CI	*P*-val	OR/Beta	95% CI	*P*-val	OR/Beta	95% CI	*P*-val
**Amino acid**											
	Tryptophan	18	1.007	(0.993,1.021)	0.329	1.159	(0.97,1.386)	0.125	1.004	(0.988,1.021)	0.603
	4-acetamidobutanoate	6	1	(0.994,1.006)	0.918	0.994	(0.979,1.009)	0.466	0.997	(0.989,1.004)	0.363
	Proline	4	1.001	(0.995,1.007)	0.788	0.998	(0.985,1.01)	0.764	1	(0.994,1.006)	0.974
	Citrulline	4	1.006	(0.994,1.019)	0.33	0.922	(0.741,1.147)	0.543	1.003	(0.989,1.018)	0.655
	Betaine	5	1.001	(0.995,1.007)	0.701	1.006	(0.976,1.038)	0.713	1.002	(0.995,1.009)	0.653
	Kynurenine	6	0.998	(0.992,1.004)	0.588	1	(0.987,1.012)	0.975	1.001	(0.993,1.009)	0.763
	3-methyl-2-oxovalerate	3	1.006	(0.989,1.022)	0.504	1.082	(0.994,1.178)	0.318	1.003	(0.988,1.018)	0.715
	N-acetylglycine	7	1	(0.998,1.003)	0.766	0.997	(0.992,1.003)	0.358	1	(0.998,1.003)	0.947
	Serine	3	0.998	(0.99,1.006)	0.633	0.999	(0.914,1.091)	0.981	0.998	(0.989,1.007)	0.644
	Pyroglutamine	5	0.999	(0.996,1.003)	0.772	0.998	(0.987,1.009)	0.749	0.999	(0.995,1.003)	0.642
	Isobutyrylcarnitine	8	0.998	(0.996,1.001)	0.139	0.995	(0.988,1.002)	0.194	0.998	(0.995,1.001)	0.178
	Alpha-hydroxyisovalerate	3	1.002	(0.997,1.007)	0.423	0.995	(0.98,1.011)	0.674	1.001	(0.996,1.006)	0.733
	Asparagine	3	1.004	(0.996,1.012)	0.366	1.008	(0.988,1.028)	0.581	1.004	(0.995,1.013)	0.422
	Isovalerylcarnitine	7	1.003	(1,1.007)	0.088	0.997	(0.975,1.02)	0.824	1.002	(0.998,1.007)	0.327
	Glutaroyl carnitine	11	1.003	(1,1.007)	0.059	1.005	(0.992,1.018)	0.488	1.003	(0.998,1.007)	0.217
	Tryptophan betaine	4	1.001	(0.999,1.003)	0.401	0.998	(0.993,1.004)	0.665	1	(0.998,1.003)	0.658
	Alanine	52	1.822	(0.818,4.059)	0.142	1.891	(0.233,15.361)	0.554	1.855	(0.56,6.147)	0.312
	Creatinine	91	0.725	(0.342,1.535)	0.4	0.516	(0.057,4.635)	0.556	0.61	(0.2,1.858)	0.384
	Glutamine	98	0.765	(0.485,1.209)	0.252	0.701	(0.333,1.474)	0.351	0.752	(0.336,1.684)	0.488
	Glycine	221	0.935	(0.753,1.16)	0.54	1.003	(0.753,1.335)	0.985	0.945	(0.658,1.359)	0.761
	Histidine	45	3.271	(1.429,7.485)	0.005	2.334	(0.443,12.291)	0.323	2.057	(0.599,7.066)	0.252

	Isoleucine	18	3.287	(0.884,12.217)	0.076	7.419	(0.3,183.704)	0.239	5.066	(0.908,28.249)	0.064
	Leucine	34	1.761	(0.667,4.65)	0.253	5.632	(0.914,34.707)	0.072	2.969	(0.72,12.244)	0.132
	Phenylalanine	33	0.422	(0.169,1.053)	0.065	0.144	(0.022,0.923)	0.049	0.42	(0.107,1.643)	0.213
	Tyrosine	76	1.203	(0.732,1.978)	0.466	2.195	(0.959,5.022)	0.067	1.576	(0.713,3.482)	0.261
	Valine	49	1.736	(0.806,3.736)	0.159	4.395	(0.999,19.345)	0.056	2.078	(0.637,6.785)	0.226
**Carbohydrate**											
	Mannose	6	1.001	(0.997,1.005)	0.673	1	(0.989,1.012)	0.944	1	(0.995,1.005)	0.956
	1,5-anhydroglucitol (1,5-AG)	6	0.999	(0.995,1.003)	0.547	1.004	(0.991,1.017)	0.596	1	(0.995,1.004)	0.915
	Erythronate	3	1.01	(0.994,1.026)	0.209	1.082	(0.962,1.216)	0.416	1.006	(0.99,1.021)	0.48
	Glucose	38	1.821	(0.7,4.743)	0.219	2.907	(0.311,27.203)	0.356	2.171	(0.489,9.638)	0.308
	Lactate	16	1.335	(0.195,9.134)	0.768	2.191	(0.001,3846.92)	0.84	1.229	(0.126,11.961)	0.859
	Pyruvate	60	1.298	(0.651,2.588)	0.459	2.122	(0.498,9.051)	0.313	1.69	(0.593,4.812)	0.326
**Cofactors and vitamins**											
	Biliverdin	9	0.999	(0.997,1.001)	0.295	1	(0.996,1.003)	0.809	0.999	(0.997,1.001)	0.601
	Bilirubin (Z,Z)	8	0.999	(0.998,1)	0.171	1	(0.998,1.003)	0.727	1	(0.998,1.001)	0.527
	Bilirubin (E,E)	7	0.999	(0.997,1)	0.155	1.001	(0.998,1.004)	0.715	0.999	(0.998,1.001)	0.538
	Bilirubin (E,Z or Z,E)	4	0.999	(0.997,1.002)	0.612	0.999	(0.993,1.006)	0.873	0.999	(0.996,1.002)	0.684
	Acetate	20	1.665	(0.392,7.08)	0.49	0.299	(0.006,15.076)	0.554	1.181	(0.168,8.282)	0.867
**Energy**											
	Succinylcarnitine	10	0.996	(0.992,1.001)	0.153	0.995	(0.985,1.006)	0.407	0.996	(0.99,1.002)	0.176
	Acetone	19	6.225	(1.496,25.895)	0.012	3.804	(0.149,97.168)	0.43	7.342	(1.006,53.598)	0.049
	Citrate	80	0.972	(0.541,1.749)	0.925	2.686	(0.923,7.817)	0.074	1.069	(0.434,2.633)	0.886
**Lipid**											
	Arachidonate (20:4n6)	5	0.998	(0.994,1.002)	0.339	0.996	(0.983,1.009)	0.566	0.998	(0.993,1.002)	0.321
	Carnitine	21	0.998	(0.99,1.005)	0.505	0.994	(0.98,1.009)	0.443	0.996	(0.987,1.006)	0.444

	2-hydroxyisobutyrate	4	1.003	(0.997,1.01)	0.27	1.004	(0.984,1.025)	0.716	1.003	(0.996,1.011)	0.346
	Androsterone sulfate	8	0.999	(0.999,1)	0.092	0.999	(0.998,1)	0.164	1	(0.999,1.001)	0.441
	Hexanoylcarnitine	9	1.002	(0.999,1.004)	0.21	1.004	(0.998,1.01)	0.252	1.002	(0.999,1.006)	0.233
	Butyrylcarnitine	25	0.999	(0.998,1)	0.064	0.999	(0.997,1.001)	0.286	0.999	(0.998,1)	0.219
	Propionylcarnitine	5	0.995	(0.987,1.002)	0.178	0.984	(0.966,1.003)	0.204	0.995	(0.986,1.004)	0.257
	10-undecenoate (11:1n1)	4	1	(0.996,1.005)	0.853	1.001	(0.991,1.011)	0.863	1.001	(0.996,1.006)	0.792
	3-dehydrocarnitine	6	0.998	(0.992,1.004)	0.517	1.005	(0.98,1.031)	0.704	0.999	(0.992,1.007)	0.878
	1-arachidonoylglycerophosphocholine	5	0.998	(0.994,1.001)	0.236	0.996	(0.989,1.003)	0.36	0.997	(0.993,1.001)	0.216
	Octanoylcarnitine	7	1.001	(0.999,1.004)	0.342	1.002	(0.995,1.008)	0.672	1.002	(0.999,1.005)	0.232
	Decanoylcarnitine	5	1.002	(0.999,1.005)	0.158	1.001	(0.993,1.01)	0.787	1.002	(0.999,1.006)	0.17
	Epiandrosterone sulfate	7	1	(0.998,1.001)	0.777	1	(0.997,1.002)	0.732	1	(0.998,1.001)	0.618
	1-arachidonoylglycerophosphoinositol	5	0.999	(0.995,1.004)	0.791	1.004	(0.988,1.02)	0.667	1	(0.994,1.006)	0.982
	1-arachidonoylglycerophosphoethanolamine	4	0.999	(0.994,1.004)	0.635	0.991	(0.975,1.007)	0.374	1	(0.994,1.006)	0.883
	Tetradecanedioate	4	1	(0.998,1.003)	0.712	1.002	(0.997,1.008)	0.541	1	(0.998,1.003)	0.753
	Hexadecanedioate	5	1.001	(0.999,1.004)	0.289	1.002	(0.995,1.009)	0.547	1.001	(0.998,1.003)	0.62
	Dihomo-linolenate (20:3n3 or n6)	3	1	(0.986,1.015)	0.951	0.974	(0.947,1.002)	0.316	0.997	(0.987,1.008)	0.603
	Octadecanedioate	4	1.004	(0.999,1.009)	0.137	1.014	(0.989,1.04)	0.396	1.003	(0.997,1.008)	0.395
	5alpha-androstan-3beta,17beta-diol disulfate	6	0.998	(0.997,1)	0.029	0.998	(0.996,1.001)	0.291	0.998	(0.997,1)	0.07
	4-androsten-3beta,17beta-diol disulfate 1	6	0.999	(0.997,1)	0.154	0.998	(0.996,1.001)	0.337	0.999	(0.997,1.001)	0.167
	Cis-4-decenoyl carnitine	5	1.003	(0.999,1.006)	0.132	1.001	(0.992,1.01)	0.866	1.003	(0.999,1.007)	0.121
	22:6, docosahexaenoic acid	6	0.63	(0.273,1.452)	0.278	0.612	(0.018,21.265)	0.8	0.546	(0.201,1.485)	0.236
	Acetoacetate	9	10.369	(1.223,87.881)	0.032	17.909	(0.02,19378.81)	0.445	7.548	(0.621,91.777)	0.113
	Apolipoprotein A1	239	1.359	(0.962,1.92)	0.082	1.531	(0.803,2.917)	0.197	1.508	(0.839,2.711)	0.169
	Apolipoprotein B	167	1.358	(0.988,1.867)	0.059	2.027	(1.18,3.482)	0.011	2.018	(1.192,3.418)	0.009
	3-Hydroxybutyrate	25	1.261	(0.338,4.702)	0.73	1.023	(0.03,34.906)	0.99	0.493	(0.087,2.8)	0.425

	Total cholines	181	1.55	(1.06,2.265)	0.024	1.151	(0.547,2.419)	0.712	1.457	(0.728,2.915)	0.287
	Docosahexaenoic acid	161	0.863	(0.609,1.222)	0.406	0.647	(0.373,1.124)	0.125	0.641	(0.368,1.117)	0.116
	Glycoprotein acetyls	171	0.687	(0.474,0.995)	0.047	0.51	(0.257,1.013)	0.056	0.795	(0.447,1.413)	0.434
	HDL cholesterol	280	1.358	(0.984,1.875)	0.063	1.212	(0.687,2.139)	0.508	1.31	(0.761,2.257)	0.33
	Linoleic acid	179	1.441	(0.98,2.119)	0.063	1.354	(0.61,3.007)	0.458	1.778	(0.964,3.28)	0.065
	LDL cholesterol	153	1.261	(0.909,1.749)	0.165	1.463	(0.854,2.505)	0.168	1.538	(0.927,2.553)	0.096
	Monounsaturated fatty acids	212	1.169	(0.814,1.679)	0.397	1.061	(0.541,2.082)	0.863	1.14	(0.628,2.071)	0.667
	Phosphatidylcholines	191	1.592	(1.114,2.274)	0.011	1.268	(0.646,2.487)	0.491	1.465	(0.771,2.785)	0.244
	Phosphoglycerides	176	1.562	(1.074,2.271)	0.02	1.11	(0.54,2.281)	0.776	1.434	(0.724,2.841)	0.301
	Polyunsaturated fatty acids	222	1.237	(0.873,1.752)	0.232	0.818	(0.413,1.62)	0.565	1.218	(0.716,2.072)	0.466
	Saturated fatty acids	158	1.281	(0.834,1.968)	0.258	1.433	(0.612,3.359)	0.409	1.091	(0.543,2.195)	0.806
	Sphingomyelins	189	1.185	(0.824,1.706)	0.36	1.773	(0.937,3.356)	0.08	1.74	(0.969,3.126)	0.064
	Total cholesterol	165	1.355	(0.941,1.95)	0.102	1.754	(0.926,3.321)	0.087	2.139	(1.169,3.915)	0.014
	Total esterified cholesterol	169	1.313	(0.911,1.891)	0.144	1.728	(0.908,3.29)	0.098	2.12	(1.156,3.891)	0.015
	Total fatty acids	195	1.095	(0.745,1.609)	0.644	1.187	(0.57,2.471)	0.647	1.122	(0.591,2.131)	0.724
	Total free cholesterol	170	1.435	(1.004,2.05)	0.047	1.861	(0.997,3.475)	0.053	2.227	(1.245,3.985)	0.007
	Total triglycerides	240	1.054	(0.757,1.468)	0.756	1.072	(0.6,1.915)	0.815	1.071	(0.605,1.897)	0.813
	VLDL cholesterol	188	1.187	(0.836,1.686)	0.338	0.993	(0.493,2.001)	0.985	1.427	(0.818,2.488)	0.21
**Nucleotide**											
	Uridine	3	0.996	(0.977,1.015)	0.694	0.933	(0.869,1.002)	0.309	1.002	(0.984,1.02)	0.829
	Urate	5	0.992	(0.984,1.001)	0.072	1.004	(0.988,1.019)	0.676	0.997	(0.989,1.005)	0.397
**Peptide**											
	Gamma-glutamyltyrosine	5	0.993	(0.98,1.007)	0.312	1.083	(0.983,1.195)	0.206	0.999	(0.983,1.015)	0.881
	N-acetylornithine	10	1.001	(1,1.002)	0.104	1.002	(1,1.005)	0.077	1.001	(1,1.003)	0.06
	HWESASXX	3	0.997	(0.993,1.001)	0.092	0.997	(0.983,1.011)	0.737	0.997	(0.993,1.002)	0.234

	Bradykinin, des-arg(9)	5	1	(0.999,1.001)	0.692	1	(0.997,1.003)	0.97	1	(0.999,1.001)	0.839
	Glycoproteins	82	0.811	(0.647,1.018)	0.071	1.002	(0.665,1.508)	0.993	0.924	(0.664,1.286)	0.638
	Albumin	48	1.81	(0.822,3.982)	0.14	2.044	(0.509,8.207)	0.319	2.159	(0.565,8.254)	0.261
**Unknown**											
	X-03094	5	1.002	(0.994,1.009)	0.642	1.021	(0.992,1.05)	0.254	1.003	(0.994,1.012)	0.56
	X-18601	3	1	(0.996,1.003)	0.806	1.001	(0.969,1.035)	0.945	1	(0.995,1.005)	0.996
	X-08402	6	0.999	(0.993,1.005)	0.649	1.003	(0.993,1.014)	0.558	1	(0.995,1.005)	0.933
	X-08988	3	0.997	(0.989,1.004)	0.397	1.003	(0.983,1.023)	0.815	0.998	(0.991,1.005)	0.625
	X-10510	3	0.998	(0.992,1.005)	0.659	1.001	(0.986,1.016)	0.944	0.998	(0.991,1.005)	0.649
	X-11204	3	0.995	(0.98,1.009)	0.464	0.882	(0.47,1.657)	0.764	0.996	(0.979,1.014)	0.649
	X-02269	4	1.001	(0.998,1.004)	0.452	0.999	(0.982,1.016)	0.908	1.002	(0.999,1.005)	0.268
	X-11261	6	0.997	(0.994,1.001)	0.114	0.995	(0.984,1.005)	0.361	0.997	(0.993,1.001)	0.121
	X-11315	3	1	(0.992,1.008)	0.962	0.987	(0.956,1.019)	0.562	1.002	(0.992,1.011)	0.713
	X-03056	8	0.995	(0.991,0.999)	0.018	0.998	(0.987,1.008)	0.67	0.996	(0.992,1.001)	0.144
	X-09789	3	0.996	(0.992,1)	0.041	1	(0.98,1.021)	0.988	0.996	(0.992,1)	0.055
	X-11440	6	0.999	(0.997,1)	0.101	0.998	(0.995,1.002)	0.372	0.999	(0.997,1)	0.127
	X-11441	6	0.999	(0.997,1.001)	0.252	1	(0.996,1.005)	0.865	0.999	(0.997,1.002)	0.649
	X-11442	7	0.999	(0.997,1.001)	0.357	1	(0.996,1.003)	0.831	1	(0.997,1.002)	0.692
	X-11444	5	1.004	(0.999,1.008)	0.097	0.989	(0.973,1.005)	0.257	1.003	(0.997,1.009)	0.346
	X-11445	3	0.999	(0.996,1.002)	0.366	0.986	(0.943,1.03)	0.639	0.997	(0.994,1.001)	0.111
	X-11469	5	1.001	(0.999,1.004)	0.351	1.006	(0.983,1.029)	0.663	1.002	(0.999,1.005)	0.263
	X-11491	4	1.001	(0.999,1.004)	0.396	1.001	(0.99,1.012)	0.826	1.001	(0.998,1.004)	0.578
	X-11529	11	1	(0.999,1.001)	0.83	1.001	(1,1.002)	0.233	1	(0.999,1.001)	0.708
	X-11530	8	0.999	(0.997,1.001)	0.168	1	(0.997,1.004)	0.932	0.999	(0.997,1.002)	0.586
	X-11538	8	1.001	(0.999,1.003)	0.415	1.003	(0.999,1.006)	0.215	1.001	(0.998,1.003)	0.595

	X-11593--O-methylascorbate	13	0.998	(0.994,1.001)	0.223	0.996	(0.989,1.003)	0.298	0.998	(0.994,1.002)	0.324
	X-11787	8	1.006	(1,1.013)	0.07	1.008	(0.994,1.022)	0.323	1.006	(0.998,1.014)	0.122
	X-11792	3	0.999	(0.998,1.001)	0.495	1.002	(0.996,1.009)	0.629	1	(0.998,1.002)	0.816
	X-11793--oxidized bilirubin	10	0.999	(0.997,1.001)	0.286	1	(0.996,1.005)	0.893	0.999	(0.997,1.002)	0.671
	X-11905	3	1	(0.997,1.003)	0.811	1.005	(0.997,1.013)	0.432	1.001	(0.998,1.004)	0.568
	X-12063	15	1.001	(1,1.002)	0.159	1	(0.997,1.002)	0.893	1.001	(0.999,1.002)	0.383
	X-12092	19	1	(0.999,1.001)	0.85	1	(0.999,1.001)	0.45	1	(0.999,1.001)	0.904
	X-12093	5	0.999	(0.998,1.001)	0.414	0.997	(0.992,1.002)	0.335	0.999	(0.997,1.001)	0.505
	X-12244--N-acetylcarnosine	6	0.999	(0.993,1.004)	0.633	0.971	(0.935,1.008)	0.202	1.001	(0.993,1.008)	0.877
	X-12456	3	1	(0.996,1.004)	0.906	1.009	(0.997,1.02)	0.384	1.001	(0.998,1.004)	0.589
	X-12510--2-aminooctanoic acid	7	1.002	(0.999,1.004)	0.129	1.002	(0.998,1.007)	0.347	1.002	(1,1.005)	0.088
	X-12556	4	0.999	(0.991,1.008)	0.89	0.994	(0.906,1.091)	0.915	1	(0.991,1.009)	0.984
	X-12644	3	1.001	(0.993,1.009)	0.891	0.99	(0.934,1.05)	0.801	1.003	(0.994,1.013)	0.492
	X-12696	5	1	(0.995,1.005)	0.986	0.997	(0.978,1.016)	0.778	1	(0.994,1.006)	0.986
	X-12728	8	1	(1,1)	0.205	1	(0.999,1.001)	0.861	1	(1,1.001)	0.309
	X-12798	13	1	(0.998,1.002)	0.953	1	(0.996,1.004)	0.991	1	(0.998,1.002)	0.997
	X-12844	4	1.008	(1.002,1.014)	0.014	1.019	(0.974,1.065)	0.502	1.009	(1.001,1.016)	0.02
	X-12850	3	1	(0.996,1.004)	0.905	1.004	(0.992,1.015)	0.64	1.002	(0.998,1.005)	0.378
	X-13429	4	1	(0.998,1.002)	0.87	1.003	(0.999,1.007)	0.263	1	(0.999,1.002)	0.762
	X-13431--nonanoylcarnitine	7	1.001	(0.999,1.003)	0.222	1	(0.996,1.005)	0.933	1.001	(0.999,1.003)	0.395
	X-13435	3	0.998	(0.992,1.003)	0.384	0.987	(0.968,1.007)	0.417	0.998	(0.991,1.004)	0.487
	X-14205--alpha-glutamyltyrosine	3	1.001	(0.997,1.005)	0.523	0.998	(0.987,1.009)	0.754	1	(0.995,1.005)	0.986
	X-14626	3	1.002	(0.997,1.006)	0.51	1.004	(0.994,1.014)	0.604	1.002	(0.997,1.007)	0.434

Supplementary Table 3D. MR results in Gastric Cancer.

Category	Exposures	No.of SNPs	Inverse variance weighting			MR-Egger			Weighted Median		
			OR/Beta	95% CI	*P*-val	OR/Beta	95% CI	*P*-val	OR/Beta	95% CI	*P*-val
**Amino acid**											
	Tryptophan	18	0.996	(0.977,1.015)	0.677	1.162	(0.902,1.496)	0.263	0.996	(0.973,1.021)	0.772
	4-acetamidobutanoate	6	1.002	(0.992,1.012)	0.665	1.004	(0.979,1.03)	0.786	1.002	(0.991,1.014)	0.718
	Proline	4	1.002	(0.992,1.012)	0.655	0.997	(0.977,1.018)	0.805	1	(0.99,1.011)	0.985
	Citrulline	4	0.978	(0.958,0.999)	0.038	1.004	(0.699,1.441)	0.986	0.974	(0.95,0.999)	0.039
	Betaine	5	0.996	(0.986,1.006)	0.431	1.038	(0.986,1.092)	0.25	0.992	(0.98,1.005)	0.252
	Kynurenine	6	1.008	(0.998,1.017)	0.104	1.005	(0.987,1.022)	0.62	1.007	(0.996,1.018)	0.238
	3-methyl-2-oxovalerate	3	0.998	(0.978,1.019)	0.87	1.03	(0.895,1.185)	0.753	1.003	(0.98,1.026)	0.817
	N-acetylglycine	7	1.001	(0.997,1.005)	0.579	1.005	(0.997,1.013)	0.286	1.002	(0.997,1.006)	0.479
	Serine	3	1.004	(0.986,1.023)	0.641	1.059	(0.808,1.388)	0.748	1.009	(0.994,1.025)	0.246
	Pyroglutamine	5	1.001	(0.995,1.007)	0.788	0.996	(0.979,1.014)	0.705	1	(0.992,1.007)	0.895
	Isobutyrylcarnitine	8	0.997	(0.993,1.001)	0.155	1.005	(0.994,1.016)	0.444	0.998	(0.993,1.003)	0.514
	Alpha-hydroxyisovalerate	3	1.006	(0.997,1.015)	0.194	0.998	(0.958,1.039)	0.942	1.004	(0.995,1.013)	0.369
	Asparagine	3	0.998	(0.977,1.019)	0.845	0.979	(0.922,1.04)	0.62	0.993	(0.978,1.008)	0.364
	Isovalerylcarnitine	7	1.002	(0.996,1.008)	0.569	1.008	(0.972,1.046)	0.681	1.003	(0.995,1.011)	0.502
	Glutaroyl carnitine	11	0.995	(0.99,1.001)	0.095	1	(0.978,1.023)	0.981	0.997	(0.99,1.005)	0.489
	Tryptophan betaine	4	1.001	(0.998,1.004)	0.557	0.994	(0.984,1.004)	0.346	1	(0.997,1.004)	0.896
	Alanine	52	1.174	(0.722,1.909)	0.519	1.075	(0.301,3.842)	0.912	0.894	(0.427,1.873)	0.767
	Creatinine	91	1.232	(0.776,1.956)	0.376	1.099	(0.282,4.283)	0.892	1.073	(0.54,2.133)	0.84
	Glutamine	98	1.011	(0.763,1.341)	0.937	0.78	(0.494,1.231)	0.288	1.034	(0.634,1.688)	0.892
	Glycine	221	1.045	(0.922,1.186)	0.489	1.058	(0.895,1.25)	0.51	1.055	(0.857,1.298)	0.617
	Histidine	45	0.496	(0.303,0.811)	0.005	0.505	(0.19,1.346)	0.179	0.62	(0.292,1.319)	0.215

	Isoleucine	18	1.112	(0.521,2.375)	0.783	1.08	(0.175,6.673)	0.935	1.37	(0.455,4.126)	0.575
	Leucine	34	1.012	(0.574,1.782)	0.968	0.794	(0.269,2.344)	0.679	1.341	(0.542,3.314)	0.525
	Phenylalanine	33	0.736	(0.422,1.283)	0.279	0.549	(0.178,1.696)	0.305	0.628	(0.268,1.472)	0.285
	Tyrosine	76	0.928	(0.685,1.255)	0.626	0.706	(0.426,1.168)	0.18	0.735	(0.458,1.178)	0.2
	Valine	49	1.004	(0.63,1.601)	0.986	1.08	(0.438,2.663)	0.868	1.289	(0.597,2.783)	0.518
**Carbohydrate**											
	Mannose	6	0.997	(0.99,1.004)	0.42	1.004	(0.985,1.023)	0.701	0.999	(0.991,1.008)	0.85
	1,5-anhydroglucitol (1,5-AG)	6	0.998	(0.992,1.005)	0.581	0.992	(0.97,1.014)	0.497	0.996	(0.988,1.003)	0.251
	Erythronate	3	1.027	(1.004,1.051)	0.021	0.994	(0.828,1.193)	0.958	1.028	(1.002,1.056)	0.037
	Glucose	38	1.009	(0.583,1.747)	0.974	0.352	(0.099,1.253)	0.116	0.688	(0.299,1.581)	0.378
	Lactate	16	0.665	(0.266,1.663)	0.383	0.92	(0.026,32.32)	0.964	1.01	(0.269,3.789)	0.988
	Pyruvate	60	1.077	(0.704,1.646)	0.733	1.191	(0.485,2.921)	0.704	1.06	(0.568,1.979)	0.855
**Cofactors and vitamins**											
	Biliverdin	9	0.996	(0.993,0.999)	0.009	0.995	(0.99,1.001)	0.161	0.996	(0.993,0.999)	0.015
	Bilirubin (Z,Z)	8	0.997	(0.995,0.999)	0.003	0.997	(0.993,1.001)	0.227	0.997	(0.995,0.999)	0.009
	Bilirubin (E,E)	7	0.996	(0.994,0.999)	0.005	0.996	(0.991,1.002)	0.233	0.996	(0.993,0.999)	0.008
	Bilirubin (E,Z or Z,E)	4	0.994	(0.989,0.998)	0.005	0.993	(0.982,1.004)	0.329	0.994	(0.989,0.998)	0.009
	Acetate	20	1.333	(0.553,3.215)	0.522	2.109	(0.195,22.839)	0.547	1.184	(0.365,3.845)	0.778
**Energy**											
	Succinylcarnitine	10	0.998	(0.99,1.006)	0.578	0.99	(0.974,1.007)	0.286	0.994	(0.984,1.004)	0.265
	Acetone	19	3.034	(1.314,7.003)	0.009	7.602	(1.187,48.664)	0.047	3.886	(1.13,13.364)	0.031
	Citrate	80	1.112	(0.803,1.539)	0.522	0.621	(0.34,1.132)	0.124	0.891	(0.54,1.469)	0.651
**Lipid**											
	Arachidonate (20:4n6)	5	0.996	(0.989,1.003)	0.22	0.997	(0.971,1.023)	0.811	0.996	(0.988,1.003)	0.274
	Carnitine	21	1.003	(0.991,1.015)	0.633	0.99	(0.967,1.015)	0.45	0.998	(0.981,1.014)	0.771

	2-hydroxyisobutyrate	4	1.001	(0.991,1.011)	0.886	0.982	(0.954,1.011)	0.35	0.996	(0.984,1.009)	0.578
	Androsterone sulfate	8	0.999	(0.998,1.001)	0.414	1.001	(0.999,1.002)	0.408	1	(0.999,1.001)	0.88
	Hexanoylcarnitine	9	1.001	(0.997,1.006)	0.529	1.003	(0.993,1.012)	0.621	1.001	(0.995,1.007)	0.77
	Butyrylcarnitine	25	1.001	(0.999,1.002)	0.515	1	(0.996,1.003)	0.868	1	(0.998,1.002)	0.88
	Propionylcarnitine	5	0.997	(0.984,1.009)	0.588	0.996	(0.965,1.028)	0.807	0.997	(0.983,1.012)	0.683
	10-undecenoate (11:1n1)	4	1.003	(0.996,1.01)	0.461	1.004	(0.988,1.02)	0.671	1.003	(0.995,1.011)	0.494
	3-dehydrocarnitine	6	1.001	(0.991,1.011)	0.817	0.998	(0.957,1.041)	0.938	0.997	(0.985,1.01)	0.665
	1-arachidonoylglycerophosphocholine	5	0.995	(0.988,1.002)	0.128	0.991	(0.975,1.007)	0.352	0.994	(0.987,1.001)	0.087
	Octanoylcarnitine	7	1.002	(0.998,1.006)	0.447	0.996	(0.984,1.007)	0.473	1	(0.995,1.005)	0.918
	Decanoylcarnitine	5	1.001	(0.996,1.006)	0.83	0.997	(0.983,1.011)	0.718	1	(0.994,1.006)	0.934
	Epiandrosterone sulfate	7	0.999	(0.997,1.001)	0.322	1.001	(0.998,1.004)	0.443	0.999	(0.997,1.002)	0.516
	1-arachidonoylglycerophosphoinositol	5	0.997	(0.989,1.005)	0.437	1.013	(0.986,1.04)	0.42	1.001	(0.992,1.01)	0.861
	1-arachidonoylglycerophosphoethanolamine	4	0.994	(0.982,1.006)	0.304	1.01	(0.973,1.049)	0.653	0.993	(0.984,1.003)	0.169
	Tetradecanedioate	4	0.999	(0.995,1.002)	0.49	1.003	(0.993,1.012)	0.634	0.999	(0.995,1.003)	0.502
	Hexadecanedioate	5	0.999	(0.995,1.003)	0.635	1.003	(0.992,1.013)	0.647	0.999	(0.995,1.004)	0.758
	Dihomo-linolenate (20:3n3 or n6)	3	1.008	(0.995,1.022)	0.222	1.01	(0.969,1.052)	0.726	1.009	(0.994,1.024)	0.264
	Octadecanedioate	4	0.997	(0.988,1.005)	0.47	0.988	(0.938,1.04)	0.682	0.998	(0.989,1.008)	0.699
	5alpha-androstan-3beta,17beta-diol disulfate	6	1	(0.998,1.002)	0.854	1.001	(0.997,1.004)	0.743	1	(0.997,1.003)	0.957
	4-androsten-3beta,17beta-diol disulfate 1	6	1	(0.998,1.002)	0.95	1.001	(0.996,1.005)	0.843	1	(0.997,1.003)	0.958
	Cis-4-decenoyl carnitine	5	1	(0.994,1.005)	0.899	1.002	(0.988,1.017)	0.775	1	(0.993,1.006)	0.891
	22:6, docosahexaenoic acid	6	0.885	(0.506,1.548)	0.669	0.132	(0.015,1.123)	0.137	0.651	(0.339,1.249)	0.197
	Acetoacetate	9	2.31	(0.741,7.196)	0.149	16.844	(0.519,546.989)	0.156	5.367	(1.176,24.504)	0.03
	Apolipoprotein A1	239	1.096	(0.883,1.36)	0.407	1.117	(0.746,1.672)	0.593	1.08	(0.753,1.549)	0.677
	Apolipoprotein B	167	1.365	(1.124,1.657)	0.002	1.574	(1.132,2.19)	0.008	1.344	(0.993,1.818)	0.056
	3-Hydroxybutyrate	25	1.342	(0.65,2.772)	0.426	1.835	(0.274,12.274)	0.537	1.558	(0.568,4.275)	0.39

	Total cholines	181	0.971	(0.766,1.232)	0.811	0.813	(0.51,1.294)	0.383	0.918	(0.622,1.353)	0.664
	Docosahexaenoic acid	161	0.776	(0.641,0.938)	0.009	0.672	(0.497,0.909)	0.011	0.736	(0.523,1.035)	0.078
	Glycoprotein acetyls	171	1.053	(0.841,1.319)	0.65	1.48	(0.975,2.245)	0.067	1.025	(0.717,1.465)	0.894
	HDL cholesterol	280	1.125	(0.92,1.376)	0.253	1.147	(0.804,1.636)	0.449	1.225	(0.854,1.757)	0.27
	Linoleic acid	179	1.263	(0.983,1.621)	0.068	0.827	(0.496,1.381)	0.47	1.114	(0.782,1.587)	0.551
	LDL cholesterol	153	1.447	(1.185,1.766)	0	1.542	(1.11,2.142)	0.011	1.391	(1.018,1.9)	0.038
	Monounsaturated fatty acids	212	0.891	(0.718,1.107)	0.297	1.106	(0.74,1.652)	0.624	1.043	(0.733,1.485)	0.816
	Phosphatidylcholines	191	1.015	(0.811,1.27)	0.896	0.8	(0.524,1.22)	0.3	0.977	(0.667,1.433)	0.906
	Phosphoglycerides	176	0.994	(0.791,1.249)	0.959	0.748	(0.483,1.159)	0.196	0.958	(0.649,1.414)	0.828
	Polyunsaturated fatty acids	222	1.029	(0.835,1.268)	0.789	0.82	(0.544,1.236)	0.344	0.976	(0.698,1.365)	0.887
	Saturated fatty acids	158	0.902	(0.699,1.163)	0.426	0.839	(0.508,1.387)	0.495	1.049	(0.681,1.617)	0.829
	Sphingomyelins	189	1.222	(0.959,1.558)	0.105	1.342	(0.874,2.06)	0.18	1.281	(0.884,1.857)	0.191
	Total cholesterol	165	1.385	(1.096,1.749)	0.006	1.297	(0.859,1.957)	0.218	1.267	(0.882,1.819)	0.2
	Total esterified cholesterol	169	1.4	(1.099,1.784)	0.006	1.481	(0.965,2.274)	0.074	1.305	(0.903,1.886)	0.156
	Total fatty acids	195	0.854	(0.683,1.068)	0.167	0.907	(0.594,1.385)	0.651	0.976	(0.686,1.388)	0.892
	Total free cholesterol	170	1.288	(1.03,1.611)	0.027	1.315	(0.887,1.949)	0.175	1.231	(0.873,1.736)	0.236
	Total triglycerides	239	0.883	(0.722,1.079)	0.223	0.912	(0.642,1.296)	0.608	1.034	(0.75,1.426)	0.837
	VLDL cholesterol	188	1.064	(0.839,1.349)	0.607	0.969	(0.603,1.557)	0.896	1.088	(0.776,1.523)	0.626
**Nucleotide**											
	Uridine	3	1.019	(0.994,1.044)	0.14	1.063	(0.944,1.196)	0.499	1.018	(0.989,1.048)	0.215
	Urate	5	1.004	(0.992,1.015)	0.529	1.008	(0.983,1.034)	0.573	1.003	(0.989,1.016)	0.685
**Peptide**											
	Gamma-glutamyltyrosine	5	0.995	(0.977,1.014)	0.608	0.973	(0.827,1.144)	0.762	0.998	(0.974,1.021)	0.844
	N-acetylornithine	10	1	(0.998,1.002)	0.976	0.999	(0.996,1.003)	0.722	0.999	(0.997,1.002)	0.512
	HWESASXX	3	1	(0.993,1.006)	0.933	1.01	(0.986,1.035)	0.558	1.001	(0.994,1.009)	0.757

	Bradykinin, des-arg(9)	5	1	(0.998,1.002)	0.965	1	(0.995,1.005)	0.968	1	(0.998,1.002)	0.889
	Glycoproteins	82	1.077	(0.963,1.203)	0.194	1.059	(0.866,1.296)	0.576	1.064	(0.869,1.303)	0.549
	Albumin	48	1.047	(0.649,1.691)	0.849	1.167	(0.5,2.725)	0.723	1.161	(0.528,2.556)	0.71
**Unknown**											
	X-03094	5	1.001	(0.989,1.014)	0.845	1.024	(0.973,1.077)	0.436	1.002	(0.986,1.018)	0.83
	X-18601	3	1.001	(0.994,1.007)	0.843	1.01	(0.971,1.051)	0.711	1.001	(0.994,1.008)	0.798
	X-08402	6	0.999	(0.99,1.008)	0.842	0.994	(0.977,1.01)	0.491	0.999	(0.991,1.006)	0.724
	X-08988	3	1.005	(0.993,1.017)	0.414	1.003	(0.977,1.03)	0.845	1.005	(0.992,1.018)	0.422
	X-10510	3	0.997	(0.985,1.008)	0.559	0.985	(0.96,1.01)	0.448	0.995	(0.983,1.008)	0.448
	X-11204	3	1.006	(0.983,1.031)	0.605	1.201	(0.359,4.017)	0.816	1.005	(0.975,1.037)	0.729
	X-02269	4	1.002	(0.997,1.006)	0.409	0.991	(0.963,1.02)	0.595	1.002	(0.997,1.008)	0.387
	X-11261	6	1	(0.995,1.005)	0.956	0.995	(0.978,1.012)	0.604	1	(0.994,1.006)	0.951
	X-11315	3	1.014	(0.994,1.034)	0.16	0.98	(0.901,1.065)	0.714	1.005	(0.987,1.023)	0.613
	X-03056	8	1.002	(0.995,1.008)	0.604	1	(0.983,1.018)	0.965	1.001	(0.993,1.009)	0.854
	X-09789	3	0.999	(0.993,1.006)	0.859	1.001	(0.968,1.035)	0.95	0.999	(0.992,1.006)	0.781
	X-11440	6	0.999	(0.996,1.002)	0.558	1	(0.995,1.005)	0.992	0.999	(0.996,1.002)	0.534
	X-11441	6	0.996	(0.992,0.999)	0.005	0.996	(0.989,1.003)	0.34	0.995	(0.992,0.999)	0.008
	X-11442	7	0.996	(0.993,0.999)	0.007	0.996	(0.99,1.002)	0.297	0.995	(0.992,0.999)	0.01
	X-11444	5	1	(0.992,1.007)	0.906	1.012	(0.986,1.039)	0.428	1	(0.991,1.009)	0.965
	X-11445	3	1	(0.996,1.004)	0.909	1.005	(0.955,1.058)	0.87	1	(0.995,1.005)	1
	X-11469	5	1.002	(0.998,1.007)	0.372	0.995	(0.958,1.033)	0.801	1.002	(0.997,1.008)	0.462
	X-11491	4	1	(0.995,1.004)	0.934	1.006	(0.988,1.025)	0.585	1.001	(0.996,1.006)	0.636
	X-11529	11	1	(0.999,1.002)	0.446	1.001	(0.999,1.003)	0.303	1.001	(0.999,1.002)	0.519
	X-11530	8	0.996	(0.993,0.999)	0.01	0.995	(0.99,1.001)	0.171	0.995	(0.992,0.999)	0.007
	X-11538	8	0.999	(0.996,1.002)	0.526	1.002	(0.996,1.007)	0.62	0.999	(0.996,1.003)	0.712

	X-11593--O-methylascorbate	13	0.993	(0.988,0.999)	0.023	0.995	(0.983,1.006)	0.371	0.995	(0.987,1.002)	0.137
	X-11787	8	1.007	(0.996,1.018)	0.245	1.003	(0.981,1.026)	0.788	1.005	(0.992,1.019)	0.447
	X-11792	3	1	(0.997,1.003)	0.906	1	(0.989,1.011)	0.964	1	(0.997,1.003)	0.992
	X-11793--oxidized bilirubin	10	0.995	(0.992,0.999)	0.01	0.994	(0.987,1.001)	0.13	0.995	(0.99,0.999)	0.008
	X-11905	3	0.998	(0.992,1.005)	0.627	1.005	(0.985,1.025)	0.718	0.998	(0.993,1.004)	0.505
	X-12063	15	1	(0.998,1.002)	0.79	1.003	(0.999,1.006)	0.167	1.001	(0.998,1.003)	0.653
	X-12092	19	1	(0.999,1.001)	0.522	1	(0.998,1.002)	0.906	0.999	(0.998,1.001)	0.391
	X-12093	5	1	(0.997,1.003)	0.93	0.998	(0.989,1.006)	0.613	0.999	(0.996,1.003)	0.608
	X-12244--N-acetylcarnosine	6	0.991	(0.977,1.004)	0.183	1.055	(0.97,1.147)	0.28	0.994	(0.982,1.007)	0.376
	X-12456	3	0.999	(0.992,1.005)	0.661	1.008	(0.98,1.036)	0.682	1	(0.994,1.005)	0.954
	X-12510--2-aminooctanoic acid	7	1	(0.996,1.004)	0.935	0.996	(0.989,1.003)	0.345	0.999	(0.995,1.003)	0.553
	X-12556	4	1.012	(1.001,1.024)	0.039	0.992	(0.877,1.121)	0.906	1.007	(0.992,1.021)	0.363
	X-12644	3	0.997	(0.984,1.01)	0.671	1.035	(0.939,1.14)	0.615	1	(0.985,1.015)	0.976
	X-12696	5	0.993	(0.985,1.001)	0.109	1.013	(0.981,1.045)	0.489	0.994	(0.985,1.004)	0.246
	X-12728	8	1	(1,1.001)	0.881	1	(0.998,1.002)	0.918	1	(0.999,1.001)	0.855
	X-12798	13	1	(0.997,1.004)	0.796	1	(0.995,1.005)	0.877	1.001	(0.997,1.005)	0.678
	X-12844	4	1.001	(0.985,1.017)	0.915	1.013	(0.888,1.156)	0.869	1.001	(0.988,1.014)	0.905
	X-12850	3	0.999	(0.994,1.004)	0.731	0.997	(0.985,1.008)	0.662	0.998	(0.993,1.004)	0.572
	X-13429	4	1	(0.997,1.002)	0.853	0.999	(0.992,1.006)	0.77	1	(0.997,1.003)	0.99
	X-13431--nonanoylcarnitine	7	0.999	(0.996,1.002)	0.464	1	(0.993,1.007)	0.994	0.999	(0.996,1.003)	0.631
	X-13435	3	1.002	(0.991,1.014)	0.689	1.011	(0.956,1.069)	0.766	0.998	(0.987,1.009)	0.663
	X-14205--alpha-glutamyltyrosine	3	1.001	(0.994,1.009)	0.754	0.992	(0.977,1.007)	0.474	1.003	(0.996,1.011)	0.404
	X-14626	3	0.997	(0.986,1.008)	0.604	1.013	(0.996,1.03)	0.367	0.997	(0.989,1.005)	0.48

Supplementary Table 3E. MR results in Pancreatic Cancer.

Category	Exposures	No.of SNPs	Inverse variance weighting			MR-Egger			Weighted Median		
			OR/Beta	95% CI	*P*-val	OR/Beta	95% CI	*P*-val	OR/Beta	95% CI	*P*-val
**Amino acid**											
	Tryptophan	18	0.99	(0.972,1.008)	0.274	0.94	(0.736,1.2)	0.625	0.988	(0.964,1.012)	0.328
	4-acetamidobutanoate	6	1.005	(0.994,1.015)	0.411	0.996	(0.968,1.025)	0.81	1.004	(0.992,1.016)	0.544
	Proline	4	1.006	(0.996,1.015)	0.255	0.999	(0.978,1.019)	0.9	1.003	(0.993,1.014)	0.531
	Citrulline	4	1.006	(0.985,1.026)	0.601	0.955	(0.67,1.361)	0.822	1.007	(0.983,1.031)	0.591
	Betaine	5	0.998	(0.988,1.008)	0.661	0.989	(0.942,1.04)	0.701	0.997	(0.985,1.008)	0.593
	Kynurenine	6	0.992	(0.983,1.001)	0.099	0.994	(0.977,1.01)	0.486	0.989	(0.977,1.001)	0.063
	3-methyl-2-oxovalerate	3	1	(0.981,1.02)	0.975	0.99	(0.863,1.136)	0.91	0.998	(0.976,1.021)	0.866
	N-acetylglycine	7	1	(0.996,1.004)	0.985	1.006	(0.998,1.013)	0.218	1.001	(0.997,1.006)	0.486
	Serine	3	1.005	(0.993,1.018)	0.406	1.04	(0.901,1.201)	0.686	1.009	(0.995,1.024)	0.217
	Pyroglutamine	5	1.001	(0.994,1.009)	0.686	0.988	(0.971,1.006)	0.273	1.002	(0.994,1.009)	0.67
	Isobutyrylcarnitine	8	1.003	(0.999,1.007)	0.142	1.005	(0.994,1.016)	0.446	1.003	(0.998,1.009)	0.219
	Alpha-hydroxyisovalerate	3	0.999	(0.991,1.006)	0.727	0.987	(0.961,1.012)	0.494	0.997	(0.988,1.005)	0.444
	Asparagine	3	1.001	(0.983,1.018)	0.955	0.974	(0.943,1.006)	0.354	1	(0.987,1.014)	0.986
	Isovalerylcarnitine	7	0.998	(0.991,1.005)	0.598	0.979	(0.937,1.024)	0.403	0.998	(0.991,1.006)	0.672
	Glutaroyl carnitine	11	0.997	(0.991,1.002)	0.219	0.993	(0.973,1.014)	0.519	0.994	(0.987,1.001)	0.08
	Tryptophan betaine	4	0.999	(0.996,1.002)	0.573	0.994	(0.984,1.003)	0.318	0.999	(0.995,1.002)	0.509
	Alanine	52	1.122	(0.682,1.848)	0.65	2.917	(0.789,10.781)	0.115	0.974	(0.467,2.031)	0.945
	Creatinine	91	1.288	(0.808,2.053)	0.288	1.867	(0.477,7.31)	0.373	1.232	(0.583,2.602)	0.584
	Glutamine	98	1.225	(0.921,1.63)	0.163	1.526	(0.961,2.424)	0.077	1.278	(0.766,2.133)	0.347
	Glycine	221	0.948	(0.833,1.078)	0.413	0.992	(0.836,1.177)	0.925	1.023	(0.822,1.273)	0.836
	Histidine	45	1.045	(0.631,1.73)	0.865	1	(0.367,2.725)	1	1.179	(0.546,2.546)	0.674

	Isoleucine	18	2.515	(1.155,5.476)	0.02	2.91	(0.448,18.902)	0.28	1.669	(0.559,4.98)	0.359
	Leucine	34	1.578	(0.883,2.822)	0.124	2.73	(0.899,8.292)	0.086	1.321	(0.54,3.23)	0.542
	Phenylalanine	33	1.162	(0.584,2.312)	0.669	1.629	(0.397,6.696)	0.503	1.473	(0.631,3.439)	0.371
	Tyrosine	76	0.881	(0.646,1.2)	0.42	0.92	(0.549,1.541)	0.752	0.981	(0.599,1.606)	0.94
	Valine	49	0.97	(0.601,1.565)	0.9	1.153	(0.456,2.916)	0.765	1.094	(0.511,2.342)	0.816
**Carbohydrate**											
	Mannose	6	1.001	(0.993,1.008)	0.858	1.004	(0.986,1.023)	0.688	1.001	(0.993,1.01)	0.748
	1,5-anhydroglucitol (1,5-AG)	6	0.999	(0.991,1.006)	0.764	0.979	(0.958,1)	0.125	0.995	(0.988,1.003)	0.222
	Erythronate	3	1.011	(0.971,1.054)	0.585	1.013	(0.636,1.612)	0.966	1.011	(0.983,1.038)	0.449
	Glucose	38	0.63	(0.344,1.154)	0.135	0.268	(0.067,1.067)	0.07	0.627	(0.237,1.66)	0.348
	Lactate	16	0.366	(0.143,0.934)	0.036	3.097	(0.091,105.516)	0.54	0.311	(0.088,1.106)	0.071
	Pyruvate	60	0.594	(0.368,0.957)	0.032	0.228	(0.086,0.602)	0.004	0.481	(0.247,0.935)	0.031
**Cofactors and vitamins**											
	Biliverdin	9	1	(0.997,1.003)	0.973	1	(0.995,1.006)	0.955	1	(0.997,1.003)	0.921
	Bilirubin (Z,Z)	8	1	(0.998,1.002)	0.87	1.001	(0.997,1.004)	0.765	1	(0.998,1.003)	0.769
	Bilirubin (E,E)	7	1	(0.998,1.003)	0.895	1	(0.995,1.005)	0.911	1	(0.998,1.003)	0.76
	Bilirubin (E,Z or Z,E)	4	1.001	(0.997,1.006)	0.615	0.999	(0.988,1.009)	0.823	1.001	(0.997,1.006)	0.641
	Acetate	20	3.098	(1.189,8.071)	0.021	2.531	(0.182,35.261)	0.498	2.82	(0.748,10.636)	0.126
**Energy**											
	Succinylcarnitine	10	0.995	(0.988,1.003)	0.242	0.993	(0.977,1.01)	0.446	0.995	(0.985,1.005)	0.298
	Acetone	19	0.604	(0.256,1.423)	0.249	0.664	(0.099,4.462)	0.679	0.698	(0.209,2.338)	0.56
	Citrate	80	0.977	(0.702,1.358)	0.889	1.108	(0.599,2.049)	0.746	0.937	(0.561,1.564)	0.803
**Lipid**											
	Arachidonate (20:4n6)	5	0.996	(0.989,1.003)	0.277	0.996	(0.97,1.022)	0.769	0.996	(0.989,1.004)	0.316
	Carnitine	21	1	(0.989,1.012)	0.962	1.016	(0.992,1.04)	0.214	1.004	(0.988,1.021)	0.613

	2-hydroxyisobutyrate	4	1	(0.99,1.01)	0.99	1.021	(0.992,1.05)	0.291	1.001	(0.989,1.013)	0.885
	Androsterone sulfate	8	1	(0.999,1.002)	0.685	0.999	(0.997,1.001)	0.551	1	(0.999,1.001)	0.988
	Hexanoylcarnitine	9	0.998	(0.994,1.002)	0.385	1.001	(0.992,1.011)	0.799	0.999	(0.994,1.005)	0.781
	Butyrylcarnitine	25	1.001	(0.999,1.002)	0.369	1.001	(0.998,1.004)	0.53	1.001	(0.999,1.003)	0.419
	Propionylcarnitine	5	1.008	(0.995,1.02)	0.233	1.011	(0.976,1.047)	0.598	1.002	(0.987,1.018)	0.77
	10-undecenoate (11:1n1)	4	0.996	(0.988,1.003)	0.238	1.004	(0.988,1.02)	0.673	0.998	(0.991,1.006)	0.664
	3-dehydrocarnitine	6	1	(0.99,1.009)	0.923	0.97	(0.931,1.01)	0.212	1.003	(0.99,1.016)	0.654
	1-arachidonoylglycerophosphocholine	5	0.999	(0.994,1.005)	0.842	0.995	(0.983,1.007)	0.504	0.999	(0.993,1.006)	0.773
	Octanoylcarnitine	7	0.998	(0.994,1.002)	0.357	1.004	(0.993,1.016)	0.48	0.999	(0.994,1.004)	0.694
	Decanoylcarnitine	5	0.997	(0.992,1.002)	0.239	1.004	(0.99,1.018)	0.598	0.997	(0.991,1.003)	0.395
	Epiandrosterone sulfate	7	1	(0.997,1.003)	0.967	0.998	(0.994,1.002)	0.389	0.999	(0.997,1.001)	0.443
	1-arachidonoylglycerophosphoinositol	5	0.994	(0.987,1.002)	0.15	0.99	(0.965,1.016)	0.509	0.994	(0.985,1.002)	0.161
	1-arachidonoylglycerophosphoethanolamine	4	0.996	(0.988,1.005)	0.406	0.987	(0.961,1.013)	0.42	0.995	(0.986,1.005)	0.301
	Tetradecanedioate	4	0.998	(0.995,1.002)	0.402	0.997	(0.988,1.006)	0.606	0.998	(0.994,1.002)	0.25
	Hexadecanedioate	5	0.998	(0.994,1.002)	0.295	0.997	(0.986,1.007)	0.566	0.997	(0.993,1.002)	0.223
	Dihomo-linolenate (20:3n3 or n6)	3	1.009	(0.996,1.023)	0.192	1.034	(0.993,1.076)	0.354	1.012	(0.996,1.028)	0.13
	Octadecanedioate	4	0.997	(0.989,1.005)	0.443	1.002	(0.962,1.043)	0.932	0.997	(0.988,1.006)	0.519
	5alpha-androstan-3beta,17beta-diol disulfate	6	1	(0.998,1.003)	0.744	0.999	(0.996,1.003)	0.76	1.001	(0.998,1.004)	0.53
	4-androsten-3beta,17beta-diol disulfate 1	6	1.001	(0.999,1.003)	0.363	1.001	(0.996,1.005)	0.809	1.001	(0.998,1.004)	0.534
	Cis-4-decenoyl carnitine	5	0.998	(0.992,1.003)	0.45	1.009	(0.994,1.023)	0.321	0.999	(0.993,1.005)	0.781
	22:6, docosahexaenoic acid	6	0.677	(0.403,1.138)	0.141	1.399	(0.157,12.448)	0.778	0.636	(0.335,1.207)	0.166
	Acetoacetate	9	0.469	(0.147,1.501)	0.202	0.321	(0.009,11.487)	0.553	0.671	(0.16,2.813)	0.585
	Apolipoprotein A1	239	0.957	(0.775,1.183)	0.686	0.885	(0.597,1.313)	0.545	0.954	(0.675,1.349)	0.791
	Apolipoprotein B	167	0.853	(0.695,1.046)	0.127	1.024	(0.723,1.451)	0.893	0.876	(0.636,1.206)	0.417
	3-Hydroxybutyrate	25	0.715	(0.34,1.502)	0.376	0.623	(0.088,4.402)	0.639	0.644	(0.24,1.724)	0.381

	Total cholines	181	0.844	(0.666,1.069)	0.159	0.715	(0.45,1.136)	0.158	0.798	(0.533,1.193)	0.271
	Docosahexaenoic acid	161	0.872	(0.717,1.06)	0.168	0.882	(0.647,1.202)	0.428	0.831	(0.585,1.179)	0.299
	Glycoprotein acetyls	171	0.825	(0.651,1.047)	0.113	0.464	(0.302,0.712)	0.001	0.711	(0.498,1.014)	0.06
	HDL cholesterol	280	0.961	(0.796,1.162)	0.683	0.931	(0.667,1.299)	0.673	0.963	(0.697,1.33)	0.82
	Linoleic acid	179	0.794	(0.626,1.008)	0.058	0.697	(0.426,1.139)	0.152	0.837	(0.571,1.227)	0.362
	LDL cholesterol	153	0.837	(0.676,1.036)	0.102	0.897	(0.63,1.278)	0.548	0.833	(0.597,1.163)	0.284
	Monounsaturated fatty acids	212	0.863	(0.691,1.077)	0.193	0.814	(0.539,1.229)	0.328	0.867	(0.597,1.259)	0.455
	Phosphatidylcholines	191	0.944	(0.756,1.179)	0.61	0.756	(0.497,1.151)	0.194	0.856	(0.587,1.25)	0.421
	Phosphoglycerides	176	0.833	(0.659,1.051)	0.124	0.717	(0.458,1.123)	0.148	0.756	(0.521,1.097)	0.141
	Polyunsaturated fatty acids	222	0.786	(0.637,0.969)	0.024	0.647	(0.428,0.978)	0.04	0.793	(0.573,1.098)	0.163
	Saturated fatty acids	158	0.734	(0.565,0.952)	0.02	0.71	(0.424,1.189)	0.195	0.804	(0.533,1.213)	0.299
	Sphingomyelins	189	0.804	(0.645,1.001)	0.051	0.957	(0.649,1.411)	0.824	0.801	(0.558,1.149)	0.228
	Total cholesterol	165	0.741	(0.59,0.93)	0.01	0.94	(0.629,1.405)	0.763	0.777	(0.539,1.12)	0.176
	Total esterified cholesterol	169	0.756	(0.602,0.95)	0.016	0.948	(0.632,1.422)	0.797	0.774	(0.526,1.138)	0.192
	Total fatty acids	195	0.772	(0.614,0.97)	0.026	0.678	(0.439,1.047)	0.081	0.838	(0.579,1.214)	0.35
	Total free cholesterol	170	0.778	(0.622,0.972)	0.027	0.966	(0.653,1.431)	0.865	0.825	(0.575,1.185)	0.298
	Total triglycerides	240	0.821	(0.668,1.009)	0.06	0.826	(0.576,1.185)	0.3	0.864	(0.605,1.236)	0.425
	VLDL cholesterol	188	0.823	(0.659,1.027)	0.085	0.728	(0.467,1.134)	0.161	0.853	(0.605,1.204)	0.367
**Nucleotide**											
	Uridine	3	0.971	(0.93,1.013)	0.177	1.052	(0.821,1.348)	0.758	0.985	(0.956,1.015)	0.338
	Urate	5	1.016	(1.005,1.028)	0.006	1.01	(0.985,1.035)	0.504	1.014	(1.001,1.027)	0.03
**Peptide**											
	Gamma-glutamyltyrosine	5	0.999	(0.976,1.022)	0.914	1.049	(0.838,1.313)	0.707	1.008	(0.981,1.036)	0.562
	N-acetylornithine	10	1.001	(0.999,1.003)	0.405	1.001	(0.997,1.004)	0.775	1.001	(0.999,1.004)	0.354
	HWESASXX	3	1.004	(0.997,1.01)	0.258	1.015	(0.991,1.04)	0.443	1.002	(0.994,1.009)	0.673

	Bradykinin, des-arg(9)	5	1	(0.998,1.001)	0.695	1	(0.995,1.005)	0.941	1	(0.998,1.002)	0.721
	Glycoproteins	82	0.965	(0.861,1.082)	0.541	1.061	(0.863,1.304)	0.578	1.026	(0.854,1.232)	0.786
	Albumin	48	0.579	(0.354,0.947)	0.029	0.914	(0.383,2.178)	0.839	0.791	(0.386,1.622)	0.522
**Unknown**											
	X-03094	5	1.004	(0.992,1.016)	0.497	0.998	(0.953,1.045)	0.938	1.002	(0.988,1.016)	0.778
	X-18601	3	1	(0.994,1.007)	0.945	1.016	(0.972,1.062)	0.61	1.003	(0.996,1.011)	0.403
	X-08402	6	0.992	(0.985,0.999)	0.026	0.988	(0.976,1)	0.128	0.991	(0.983,0.999)	0.022
	X-08988	3	1.006	(0.994,1.018)	0.332	1.008	(0.982,1.034)	0.662	1.006	(0.994,1.018)	0.322
	X-10510	3	0.99	(0.979,1.002)	0.093	0.975	(0.951,0.999)	0.291	0.99	(0.978,1.002)	0.105
	X-11204	3	0.99	(0.968,1.014)	0.425	1.269	(0.458,3.512)	0.726	0.987	(0.96,1.015)	0.372
	X-02269	4	1.006	(1.001,1.01)	0.014	1.003	(0.975,1.031)	0.865	1.006	(1.001,1.011)	0.031
	X-11261	6	1.004	(0.999,1.009)	0.117	1.01	(0.993,1.027)	0.327	1.004	(0.998,1.011)	0.21
	X-11315	3	1.01	(0.997,1.023)	0.136	1.001	(0.949,1.055)	0.987	1.009	(0.994,1.024)	0.237
	X-03056	8	1.003	(0.996,1.009)	0.388	1	(0.983,1.018)	0.98	1.001	(0.992,1.009)	0.897
	X-09789	3	1.004	(0.998,1.011)	0.166	1.01	(0.964,1.057)	0.75	1.007	(1,1.014)	0.065
	X-11440	6	1	(0.997,1.003)	0.882	1.002	(0.997,1.006)	0.501	1.001	(0.997,1.004)	0.745
	X-11441	6	1	(0.997,1.003)	0.984	1	(0.993,1.007)	0.954	1	(0.997,1.004)	0.768
	X-11442	7	1	(0.997,1.003)	0.965	1	(0.994,1.006)	0.961	1.001	(0.997,1.004)	0.765
	X-11444	5	1.006	(0.998,1.014)	0.165	1.018	(0.988,1.049)	0.327	1.007	(0.997,1.016)	0.176
	X-11445	3	0.999	(0.994,1.003)	0.51	0.992	(0.944,1.043)	0.81	0.998	(0.993,1.003)	0.404
	X-11469	5	1.006	(1.002,1.011)	0.004	1.005	(0.969,1.043)	0.8	1.007	(1.001,1.012)	0.016
	X-11491	4	0.997	(0.992,1.003)	0.333	0.991	(0.965,1.017)	0.554	0.999	(0.994,1.004)	0.613
	X-11529	11	1	(0.998,1.001)	0.62	1	(0.997,1.003)	0.963	1	(0.998,1.001)	0.724
	X-11530	8	1	(0.997,1.003)	0.93	1	(0.994,1.005)	0.922	1.001	(0.997,1.004)	0.757
	X-11538	8	0.999	(0.996,1.002)	0.381	0.996	(0.991,1.002)	0.257	0.998	(0.994,1.001)	0.224

	X-11593--O-methylascorbate	13	1.007	(1.002,1.013)	0.011	1.002	(0.991,1.013)	0.778	1.006	(0.999,1.013)	0.099
	X-11787	8	1.004	(0.993,1.014)	0.516	0.996	(0.973,1.018)	0.709	1.002	(0.989,1.016)	0.757
	X-11792	3	1	(0.997,1.002)	0.734	1	(0.989,1.01)	0.944	1	(0.997,1.002)	0.739
	X-11793--oxidized bilirubin	10	0.999	(0.996,1.002)	0.58	1.001	(0.994,1.007)	0.822	1	(0.996,1.004)	0.961
	X-11905	3	1	(0.994,1.005)	0.962	0.991	(0.979,1.003)	0.377	0.999	(0.994,1.004)	0.737
	X-12063	15	0.999	(0.998,1.001)	0.581	0.997	(0.993,1)	0.099	0.998	(0.996,1.001)	0.209
	X-12092	19	1.001	(1,1.002)	0.074	1.001	(0.999,1.003)	0.372	1.001	(0.999,1.002)	0.41
	X-12093	5	1	(0.998,1.003)	0.743	1.005	(0.997,1.013)	0.338	1.001	(0.998,1.005)	0.361
	X-12244--N-acetylcarnosine	6	1.004	(0.989,1.019)	0.589	1.029	(0.92,1.151)	0.645	1.01	(0.998,1.023)	0.102
	X-12456	3	1	(0.995,1.004)	0.867	0.992	(0.974,1.01)	0.526	1	(0.994,1.005)	0.883
	X-12510--2-aminooctanoic acid	7	1	(0.996,1.004)	0.966	0.996	(0.99,1.003)	0.309	1	(0.996,1.004)	0.996
	X-12556	4	0.997	(0.983,1.011)	0.678	1.103	(0.995,1.222)	0.202	0.999	(0.986,1.013)	0.889
	X-12644	3	0.997	(0.981,1.014)	0.753	0.916	(0.833,1.007)	0.32	0.991	(0.975,1.006)	0.234
	X-12696	5	0.997	(0.989,1.005)	0.531	0.983	(0.953,1.014)	0.352	0.994	(0.984,1.004)	0.225
	X-12728	8	1	(0.999,1)	0.715	1	(0.998,1.002)	0.95	1	(1,1.001)	0.843
	X-12798	13	1	(0.997,1.003)	0.903	1.001	(0.996,1.006)	0.728	1	(0.996,1.004)	0.867
	X-12844	4	0.993	(0.983,1.003)	0.162	0.972	(0.909,1.039)	0.495	0.99	(0.979,1.002)	0.105
	X-12850	3	1.002	(0.998,1.007)	0.36	1.001	(0.99,1.012)	0.93	1.002	(0.997,1.007)	0.457
	X-13429	4	0.999	(0.997,1.002)	0.587	0.996	(0.99,1.002)	0.319	0.999	(0.996,1.002)	0.529
	X-13431--nonanoylcarnitine	7	0.997	(0.994,1)	0.041	0.995	(0.988,1.002)	0.244	0.997	(0.993,1)	0.075
	X-13435	3	0.997	(0.983,1.01)	0.64	1.016	(0.959,1.076)	0.691	0.995	(0.984,1.005)	0.32
	X-14205--alpha-glutamyltyrosine	3	0.999	(0.993,1.005)	0.839	0.998	(0.984,1.013)	0.863	0.999	(0.992,1.006)	0.767
	X-14626	3	0.996	(0.989,1.003)	0.252	0.994	(0.978,1.01)	0.587	0.996	(0.988,1.003)	0.272
