# Supplementary material for: Association between human blood metabolome and the risk of gastrointestinal tumors
Source: PLoS One. 2024 May 30;19(5):e0304574. doi: 10.1371/journal.pone.0304574 (PMC11139295; doi:10.1371/journal.pone.0304574)
Supplement: S4 Table — (PDF) [file pone.0304574.s004.pdf]

Supplementary Table 4A. Sensitivity analyses in liver cancer

Category	Exposures	No.of SNPs	Inverse variance weighting			MR-Egger		MR-PRESSO
			Q	Q *P*-val	I^2	intercept	intercept *P*-val	Global Test *P*-val
**Amino acid**								
	Tryptophan	18	9.41	0.927	0	0	0.408	0.759
	4-acetamidobutanoate	6	3.477	0.627	0	0	0.95	0.443
	Proline	4	1.396	0.707	0	0	0.832	0.501
	Citrulline	4	2.707	0.439	0	0	0.785	0.928
	Betaine	5	0.96	0.916	0	0	0.867	0.194
	Kynurenine	6	4.449	0.487	0	0	0.919	0.285
	3-methyl-2-oxovalerate	3	0.39	0.823	0	0	0.948	
	N-acetylglycine	7	5.658	0.463	0	0	0.319	0.656
	Serine	3	3.912	0.141	0.489	0	0.931	
	Pyroglutamine	5	2.084	0.72	0	0	0.896	0.893
	Isobutyrylcarnitine	8	5.147	0.642	0	0	0.247	0.551
	Alpha-hydroxyisovalerate	3	0.596	0.742	0	0	0.949	
	Asparagine	3	0.426	0.808	0	0	0.884	
	Isovalerylcarnitine	7	5.606	0.469	0	0	0.736	0.267
	Glutaroyl carnitine	11	7.42	0.685	0	0	0.39	0.82
	Tryptophan betaine	4	5.755	0.124	0.479	-0.001	0.186	0.801
	Alanine	52	42.959	0.781	0	-0.047	0.256	0.947
	Creatinine	91	104.591	0.139	0.14	0.055	0.093	0.047
	Glutamine	98	141.455	0.002	0.314	-0.025	0.282	<0.001
	Glycine	221	253.978	0.058	0.134	-0.041	0.001	<0.001
	Histidine	45	36.542	0.78	0	-0.073	0.04	0.42

	Isoleucine	18	5.958	0.993	0	0.007	0.92	<0.001
	Leucine	34	20.959	0.948	0	0.029	0.445	0.068
	Phenylalanine	33	20.299	0.946	0	-0.015	0.75	0.935
	Tyrosine	76	89.719	0.118	0.164	0	0.996	0.973
	Valine	49	33.891	0.938	0	0.03	0.371	0.279
**Carbohydrate**								
	Mannose	6	9.608	0.087	0.48	0	0.174	0.293
	1,5-anhydroglucitol (1,5-AG)	6	2.423	0.788	0	0	0.71	0.738
	Erythronate	3	1.391	0.499	0	0	0.737	
	Glucose	38	25.056	0.932	0	-0.024	0.56	<0.001
	Lactate	16	5.336	0.989	0	-0.023	0.794	0.006
	Pyruvate	60	52.907	0.698	0	0.021	0.495	0.844
**Cofactors and vitamins**								
	Biliverdin	9	5.871	0.662	0	0	0.142	0.511
	Bilirubin (Z,Z)	8	4.493	0.722	0	0	0.406	0.723
	Bilirubin (E,E)	7	2.394	0.88	0	0	0.396	0.939
	Bilirubin (E,Z or Z,E)	4	3.005	0.391	0.002	0	0.463	0.748
	Acetate	20	21.569	0.306	0.119	-0.04	0.583	0.148
**Energy**								
	Succinylcarnitine	10	3.808	0.924	0	0	0.753	0.825
	Acetone	19	8.257	0.975	0	0.01	0.865	0.993
	Citrate	80	75.839	0.58	0	-0.017	0.482	0.002
**Lipid**								
	Arachidonate (20:4n6)	5	2.009	0.734	0	0	0.959	0.459
	Carnitine	21	16.324	0.696	0	0	0.291	0.44

	2-hydroxyisobutyrate	4	2.349	0.503	0	0	0.986	0.604
	Androsterone sulfate	8	9.287	0.233	0.246	0	0.412	0.841
	Hexanoylcarnitine	9	19.609	0.012	0.592	0	0.37	0.811
	Butyrylcarnitine	25	41.74	0.014	0.425	0	0.069	0.609
	Propionylcarnitine	5	3.859	0.425	0	0	0.38	0.158
	10-undecenoate (11:1n1)	4	1.298	0.73	0	0	0.502	0.773
	3-dehydrocarnitine	6	6.888	0.229	0.274	-0.001	0.287	0.657
	1-arachidonoylglycerophosphocholine	5	1.166	0.884	0	0	0.552	0.599
	Octanoylcarnitine	7	8.238	0.221	0.272	0	0.714	0.672
	Decanoylcarnitine	5	2.746	0.601	0	0	0.756	0.972
	Epiandrosterone sulfate	7	8.174	0.226	0.266	0	0.466	0.423
	1-arachidonoylglycerophosphoinositol	5	5.337	0.254	0.25	0	0.345	0.878
	1-arachidonoylglycerophosphoethanolamine	4	1.642	0.65	0	0	0.428	0.854
	Tetradecanedioate	4	0.369	0.947	0	0	0.75	0.817
	Hexadecanedioate	5	3.191	0.526	0	0	0.868	0.612
	Dihomo-linolenate (20:3n3 or n6)	3	7.27	0.026	0.725	0	0.629	
	Octadecanedioate	4	2.412	0.491	0	0	0.858	0.542
	5alpha-androstan-3beta,17beta-diol disulfate	6	4.844	0.435	0	0	0.584	0.674
	4-androsten-3beta,17beta-diol disulfate 1	6	4.078	0.538	0	0	0.759	0.458
	Cis-4-decenoyl carnitine	5	6.463	0.167	0.381	0	0.482	0.681
	22:6, docosahexaenoic acid	6	23.817	0	0.79	0.723	0.076	0.597
	Acetoacetate	9	4.509	0.809	0	0.012	0.921	0.943
	Apolipoprotein A1	239	275.024	0.05	0.135	0.005	0.717	<0.001
	Apolipoprotein B	167	191.618	0.084	0.134	0.038	0.017	0.003
	3-Hydroxybutyrate	25	30.229	0.177	0.206	0.028	0.683	<0.001

	Total cholines	181	278.038	0	0.353	-0.011	0.589	<0.001
	Docosahexaenoic acid	161	254.049	0	0.37	0.026	0.176	<0.001
	Glycoprotein acetyls	171	193.953	0.101	0.124	-0.004	0.831	<0.001
	HDL cholesterol	280	281.52	0.446	0.009	0.003	0.816	0.598
	Linoleic acid	179	275.534	0	0.354	0.02	0.367	<0.001
	LDL cholesterol	153	179.614	0.062	0.154	0.039	0.017	0.078
	Monounsaturated fatty acids	212	301.654	0	0.301	-0.02	0.27	0.127
	Phosphatidylcholines	191	297.769	0	0.362	-0.008	0.675	0.003
	Phosphoglycerides	176	272.694	0	0.358	-0.001	0.98	0.085
	Polyunsaturated fatty acids	222	364.396	0	0.394	0.015	0.443	0.043
	Saturated fatty acids	158	253.181	0	0.38	0.009	0.684	0.785
	Sphingomyelins	189	247.091	0.002	0.239	0.03	0.066	0.72
	Total cholesterol	165	219.582	0.002	0.253	0.053	0.002	0.136
	Total esterified cholesterol	169	226.046	0.002	0.257	0.054	0.002	0.963
	Total fatty acids	195	283.399	0	0.315	0.005	0.792	0.722
	Total free cholesterol	170	243.057	0	0.305	0.055	0.002	0.985
	Total triglycerides	239	343.325	0	0.307	-0.012	0.454	<0.001
	VLDL cholesterol	188	291.188	0	0.358	0.019	0.349	0.837
**Nucleotide**								
	Uridine	3	1.31	0.52	0	-0.001	0.465	
	Urate	5	3.794	0.435	0	0	0.882	0.247
**Peptide**								
	Gamma-glutamyltyrosine	5	2.653	0.617	0	0.001	0.328	0.485
	N-acetylornithine	10	6.372	0.702	0	0	0.12	0.341
	HWESASXX	3	2.058	0.357	0.028	0	0.774	

	Bradykinin, des-arg(9)	5	1.44	0.837	0	0	0.715	0.521
	Glycoproteins	82	77.913	0.577	0	-0.062	0.012	0.002
	Albumin	48	36.726	0.86	0	-0.048	0.102	<0.001
**Unknown**								
	X-03094	5	5.682	0.224	0.296	0	0.427	0.35
	X-18601	3	7.657	0.022	0.739	-0.001	0.583	
	X-08402	6	9.491	0.091	0.473	0	0.302	0.588
	X-08988	3	2.813	0.245	0.289	0	0.344	
	X-10510	3	4.98	0.083	0.598	0	0.374	
	X-11204	3	0.747	0.688	0	0.003	0.565	
	X-02269	4	8.109	0.044	0.63	0	0.674	0.55
	X-11261	6	5.757	0.331	0.131	0	0.675	0.587
	X-11315	3	1.48	0.477	0	-0.001	0.484	
	X-03056	8	4.383	0.735	0	0	0.915	0.677
	X-09789	3	0.417	0.812	0	0	0.644	
	X-11440	6	3.25	0.661	0	0	0.494	0.985
	X-11441	6	3.69	0.595	0	0	0.4	0.398
	X-11442	7	3.916	0.688	0	0	0.252	0.021
	X-11444	5	7.204	0.125	0.445	0	0.492	0.688
	X-11445	3	4.103	0.129	0.513	0.001	0.808	
	X-11469	5	7.618	0.107	0.475	0	0.662	0.6
	X-11491	4	0.195	0.978	0	0	0.835	0.281
	X-11529	11	6.514	0.77	0	0	0.476	0.034
	X-11530	8	2.425	0.933	0	0	0.413	0.703
	X-11538	8	5.998	0.54	0	0	0.461	0.104

	X-11593--O-methylascorbate	13	10.737	0.552	0	0	0.841	0.699
	X-11787	8	6.286	0.507	0	0	0.709	0.853
	X-11792	3	0.69	0.708	0	0	0.712	
	X-11793--oxidized bilirubin	10	7.407	0.595	0	0	0.194	0.944
	X-11905	3	0.181	0.913	0	0	0.8	
	X-12063	15	16.519	0.283	0.152	0	0.175	0.388
	X-12092	19	13.4	0.767	0	0	0.569	0.789
	X-12093	5	1.886	0.757	0	0	0.827	0.614
	X-12244--N-acetylcarnosine	6	2.465	0.782	0	0	0.77	0.658
	X-12456	3	1.182	0.554	0	0	0.487	
	X-12510--2-aminooctanoic acid	7	2.886	0.823	0	0	0.314	0.827
	X-12556	4	0.943	0.815	0	-0.001	0.563	0.638
	X-12644	3	5.777	0.056	0.654	0	0.908	
	X-12696	5	2.518	0.641	0	0	0.626	0.757
	X-12728	8	1.53	0.981	0	0	0.619	0.223
	X-12798	13	10.984	0.53	0	0	0.851	0.246
	X-12844	4	0.434	0.933	0	0	0.697	0.927
	X-12850	3	0.772	0.68	0	0	0.567	
	X-13429	4	1.795	0.616	0	0	0.657	0.617
	X-13431--nonanoylcarnitine	7	3.243	0.778	0	0	0.621	0.295
	X-13435	3	1.686	0.43	0	0	0.686	
	X-14205--alpha-glutamyltyrosine	3	2.364	0.307	0.154	0	0.583	
	X-14626	3	0.676	0.713	0	0	0.596	

Supplementary Table 4B. Sensitivity analyses in colorectal cancer

Category	Exposures	No.of SNPs	Inverse variance weighting			MR-Egger		MR-PRESSO
			Q	Q *P*-val	I^2	intercept	intercept *P*-val	Global Test *P*-val
**Amino acid**								
	Tryptophan	18	14.841	0.607	0	-0.001	0.56	0.683
	4-acetamidobutanoate	6	7.277	0.201	0.313	-0.001	0.24	0.224
	Proline	4	2.876	0.411	0	0	0.567	0.488
	Citrulline	4	2.526	0.471	0	-0.005	0.337	0.644
	Betaine	5	3.338	0.503	0	-0.001	0.544	0.887
	Kynurenine	6	9.874	0.079	0.494	0	0.776	0.827
	3-methyl-2-oxovalerate	3	5.07	0.079	0.606	-0.004	0.274	
	N-acetylglycine	7	7.622	0.267	0.213	0	0.801	0.265
	Serine	3	2.827	0.243	0.292	0.001	0.939	
	Pyroglutamine	5	2.627	0.622	0	0	0.785	0.425
	Isobutyrylcarnitine	8	5.454	0.605	0	0	0.963	0.999
	Alpha-hydroxyisovalerate	3	3.457	0.178	0.422	-0.001	0.35	
	Asparagine	3	2.153	0.341	0.071	0	0.662	
	Isovalerylcarnitine	7	3.02	0.806	0	0	0.791	0.485
	Glutaroyl carnitine	11	3.538	0.966	0	0.001	0.443	0.73
	Tryptophan betaine	4	1.984	0.576	0	0.001	0.613	0.706
	Alanine	52	56.736	0.27	0.101	0.019	0.177	0.263
	Creatinine	91	108.383	0.091	0.17	-0.006	0.585	0.036
	Glutamine	98	121.787	0.045	0.204	0.005	0.481	0.004
	Glycine	221	209.44	0.684	0	-0.001	0.873	0.018
	Histidine	45	59.314	0.061	0.258	-0.003	0.817	0.135

	Isoleucine	18	31.558	0.017	0.461	-0.05	0.117	0.612
	Leucine	34	38.323	0.241	0.139	-0.01	0.456	0.692
	Phenylalanine	33	28.405	0.649	0	-0.035	0.031	0.5
	Tyrosine	76	67.191	0.728	0	0.011	0.151	0.162
	Valine	49	70.09	0.02	0.315	-0.008	0.571	0.023
**Carbohydrate**								
	Mannose	6	5.638	0.343	0.113	0.001	0.46	0.906
	1,5-anhydroglucitol (1,5-AG)	6	2.461	0.782	0	0.001	0.351	0.076
	Erythronate	3	2.434	0.296	0.178	0.003	0.442	
	Glucose	38	36.503	0.492	0	0.016	0.246	0.009
	Lactate	16	21.736	0.115	0.31	-0.023	0.525	0.051
	Pyruvate	60	105.42	0	0.44	0.001	0.961	0.02
**Cofactors and vitamins**								
	Biliverdin	9	8.219	0.412	0.027	0	0.51	0.533
	Bilirubin (Z,Z)	8	4.041	0.775	0	0	0.375	0.452
	Bilirubin (E,E)	7	6.922	0.328	0.133	0	0.922	0.438
	Bilirubin (E,Z or Z,E)	4	4.884	0.181	0.386	0	0.966	0.777
	Acetate	20	32.532	0.027	0.416	0.021	0.48	0.083
**Energy**								
	Succinylcarnitine	10	9.516	0.391	0.054	-0.001	0.111	0.531
	Acetone	19	24.337	0.144	0.26	-0.012	0.613	0.013
	Citrate	80	98.634	0.067	0.199	0.002	0.86	0.247
**Lipid**								
	Arachidonate (20:4n6)	5	3.021	0.554	0	0	0.898	0.142
	Carnitine	21	23.055	0.286	0.133	0	0.894	0.833

	2-hydroxyisobutyrate	4	0.698	0.874	0	-0.001	0.532	0.63
	Androsterone sulfate	8	3.228	0.863	0	0	0.397	0.507
	Hexanoylcarnitine	9	2.838	0.944	0	0	0.508	0.51
	Butyrylcarnitine	25	38.446	0.031	0.376	0	0.189	0.633
	Propionylcarnitine	5	6.423	0.17	0.377	0	0.95	0.92
	10-undecenoate (11:1n1)	4	4.631	0.201	0.352	0.001	0.193	0.628
	3-dehydrocarnitine	6	1.259	0.939	0	0.001	0.504	0.441
	1-arachidonoylglycerophosphocholine	5	7.47	0.113	0.464	-0.001	0.11	0.881
	Octanoylcarnitine	7	1.446	0.963	0	0	0.741	0.588
	Decanoylcarnitine	5	0.152	0.997	0	0	0.812	0.115
	Epiandrosterone sulfate	7	4.242	0.644	0	0	0.545	0.406
	1-arachidonoylglycerophosphoinositol	5	11.773	0.019	0.66	0	0.783	0.648
	1-arachidonoylglycerophosphoethanolamine	4	2.51	0.474	0	-0.001	0.36	0.278
	Tetradecanedioate	4	4.465	0.215	0.328	-0.001	0.613	0.774
	Hexadecanedioate	5	4.828	0.305	0.171	0	0.658	0.808
	Dihomo-linolenate (20:3n3 or n6)	3	1.506	0.471	0	0	0.893	
	Octadecanedioate	4	0.25	0.969	0	0	0.956	0.79
	5alpha-androstan-3beta,17beta-diol disulfate	6	1.457	0.918	0	0	0.978	0.306
	4-androsten-3beta,17beta-diol disulfate 1	6	2.204	0.82	0	0	0.924	0.393
	Cis-4-decenoyl carnitine	5	0.375	0.984	0	0	0.773	0.52
	22:6, docosahexaenoic acid	6	8.046	0.154	0.379	-0.046	0.624	0.78
	Acetoacetate	9	9.959	0.268	0.197	-0.034	0.472	0.023
	Apolipoprotein A1	239	295.072	0.007	0.193	0.001	0.786	0.087
	Apolipoprotein B	167	165.195	0.503	0	0.006	0.186	<0.001
	3-Hydroxybutyrate	25	22.022	0.578	0	-0.048	0.021	0.122

	Total cholines	181	235.638	0.003	0.236	-0.006	0.339	0.242
	Docosahexaenoic acid	161	206.45	0.008	0.225	-0.01	0.096	0.189
	Glycoprotein acetyls	171	184.107	0.217	0.077	0	0.94	0.003
	HDL cholesterol	280	305.72	0.13	0.087	0.005	0.261	0.07
	Linoleic acid	179	212.459	0.04	0.162	0.003	0.632	0.018
	LDL cholesterol	153	170.917	0.14	0.111	0.004	0.475	0.215
	Monounsaturated fatty acids	212	247.346	0.044	0.147	0.004	0.439	0.74
	Phosphatidylcholines	191	231.265	0.022	0.178	-0.005	0.425	0.055
	Phosphoglycerides	176	239.713	0.001	0.27	-0.001	0.853	0.167
	Polyunsaturated fatty acids	222	276.879	0.006	0.202	-0.002	0.72	0.492
	Saturated fatty acids	158	169.324	0.237	0.073	0.005	0.434	0.065
	Sphingomyelins	189	219.677	0.057	0.144	0.007	0.193	0.225
	Total cholesterol	165	180.624	0.177	0.092	0	0.941	0.597
	Total esterified cholesterol	169	190.292	0.115	0.117	0.001	0.867	0.663
	Total fatty acids	195	220.232	0.095	0.119	0.006	0.26	<0.001
	Total free cholesterol	170	181.81	0.237	0.07	0	0.962	0.117
	Total triglycerides	240	284.996	0.022	0.161	0	0.972	0.007
	VLDL cholesterol	188	181.876	0.592	0	0.005	0.338	0.235
**Nucleotide**								
	Uridine	3	1.295	0.523	0	-0.002	0.473	
	Urate	5	2.704	0.608	0	-0.001	0.397	0.986
**Peptide**								
	Gamma-glutamyltyrosine	5	3.37	0.498	0	-0.001	0.521	0.9
	N-acetylornithine	10	17.773	0.038	0.494	0	0.516	0.805
	HWESASXX	3	3.782	0.151	0.471	-0.001	0.719	

	Bradykinin, des-arg(9)	5	2.801	0.592	0	0.001	0.2	0.964
	Glycoproteins	82	70.988	0.779	0	-0.002	0.837	0.166
	Albumin	48	70.872	0.014	0.337	0.009	0.448	0.052
**Unknown**								
	X-03094	5	4.168	0.384	0.04	0	0.811	0.575
	X-18601	3	0.706	0.702	0	0.002	0.562	
	X-08402	6	0.637	0.986	0	0	0.627	0.983
	X-08988	3	1.311	0.519	0	-0.001	0.552	
	X-10510	3	0.202	0.904	0	0	0.887	
	X-11204	3	0.193	0.908	0	0.003	0.857	
	X-02269	4	1.04	0.792	0	-0.001	0.487	0.523
	X-11261	6	6.676	0.246	0.251	0	0.972	0.825
	X-11315	3	6.113	0.047	0.673	0.005	0.252	
	X-03056	8	5.181	0.638	0	0	0.987	0.754
	X-09789	3	2.788	0.248	0.283	0.001	0.785	
	X-11440	6	4.616	0.465	0	0	0.414	0.723
	X-11441	6	2.991	0.701	0	0	0.791	0.073
	X-11442	7	3.551	0.737	0	0	0.81	0.056
	X-11444	5	1.059	0.901	0	0	0.97	0.947
	X-11445	3	0.063	0.969	0	0	0.983	
	X-11469	5	1.126	0.89	0	0	0.839	0.847
	X-11491	4	1.089	0.78	0	0.001	0.747	0.952
	X-11529	11	10.827	0.371	0.076	0	0.608	0.988
	X-11530	8	4.92	0.67	0	0	0.533	0.328
	X-11538	8	5.603	0.587	0	0	0.904	0.812

	X-11593--O-methylascorbate	13	4.517	0.972	0	0	0.663	0.607
	X-11787	8	10.782	0.148	0.351	0.001	0.293	0.498
	X-11792	3	2.838	0.242	0.295	0.002	0.373	
	X-11793--oxidized bilirubin	10	4.479	0.877	0	0	0.602	0.701
	X-11905	3	1.12	0.571	0	0.001	0.483	
	X-12063	15	9.225	0.816	0	0	0.934	0.289
	X-12092	19	13.967	0.731	0	0	0.983	0.468
	X-12093	5	3.081	0.544	0	0	0.858	0.834
	X-12244--N-acetylcarnosine	6	2.829	0.726	0	-0.001	0.428	0.517
	X-12456	3	0.131	0.937	0	0	0.948	
	X-12510--2-aminooctanoic acid	7	6.806	0.339	0.118	0	0.46	0.797
	X-12556	4	2.613	0.455	0	0	0.887	0.775
	X-12644	3	0.317	0.854	0	0	0.852	
	X-12696	5	1.678	0.795	0	0.001	0.51	0.354
	X-12728	8	12.652	0.081	0.447	-0.005	0.068	0.475
	X-12798	13	7.46	0.826	0	0	0.472	0.519
	X-12844	4	1.351	0.717	0	-0.001	0.518	0.53
	X-12850	3	0.123	0.94	0	0	0.939	
	X-13429	4	0.885	0.829	0	0	0.914	0.186
	X-13431--nonanoylcarnitine	7	8.631	0.195	0.305	0	0.667	0.971
	X-13435	3	5.993	0.05	0.666	0	0.935	
	X-14205--alpha-glutamyltyrosine	3	1.267	0.531	0	0	0.892	
	X-14626	3	0.53	0.767	0	0	0.981	

Supplementary Table 4C. Sensitivity analyses in esophageal cancer

Category	Exposures	No.of SNPs	Inverse variance weighting			MR-Egger		MR-PRESSO
			Q	Q *P*-val	I^2	intercept	intercept *P*-val	Global Test *P*-val
**Amino acid**								
	Tryptophan	18	25.691	0.08	0.338	-0.001	0.141	0.963
	4-acetamidobutanoate	6	1.712	0.887	0	0	0.451	0.586
	Proline	4	0.621	0.892	0	0	0.647	0.761
	Citrulline	4	1.147	0.766	0	0.001	0.515	0.062
	Betaine	5	0.214	0.995	0	0	0.759	0.405
	Kynurenine	6	5.828	0.323	0.142	0	0.794	0.166
	3-methyl-2-oxovalerate	3	3.671	0.16	0.455	-0.001	0.336	
	N-acetylglycine	7	8.665	0.193	0.308	0	0.248	0.81
	Serine	3	0.583	0.747	0	0	0.992	
	Pyroglutamine	5	0.691	0.952	0	0	0.805	0.834
	Isobutyrylcarnitine	8	2.404	0.934	0	0	0.366	0.946
	Alpha-hydroxyisovalerate	3	1.584	0.453	0	0	0.556	
	Asparagine	3	0.196	0.907	0	0	0.737	
	Isovalerylcarnitine	7	3.736	0.712	0	0	0.62	0.546
	Glutaroyl carnitine	11	3.753	0.958	0	0	0.816	0.791
	Tryptophan betaine	4	1.091	0.779	0	0	0.508	0.597
	Alanine	52	48.731	0.564	0	-0.002	0.97	0.306
	Creatinine	91	78.532	0.801	0	0.011	0.748	0.648
	Glutamine	98	92.371	0.614	0	0.006	0.769	0.455
	Glycine	221	238.521	0.186	0.078	-0.01	0.465	0.344
	Histidine	45	45.862	0.395	0.041	0.019	0.648	0.079

	Isoleucine	18	18.844	0.338	0.098	-0.047	0.592	0.794
	Leucine	34	35.865	0.336	0.08	-0.064	0.151	0.194
	Phenylalanine	33	30.091	0.563	0	0.07	0.202	0.283
	Tyrosine	76	62.307	0.852	0	-0.048	0.079	0.445
	Valine	49	43.188	0.67	0	-0.055	0.158	0.491
**Carbohydrate**								
	Mannose	6	2.114	0.833	0	0	0.929	0.959
	1,5-anhydroglucitol (1,5-AG)	6	1.341	0.931	0	0	0.48	0.735
	Erythronate	3	2.634	0.268	0.241	-0.001	0.456	
	Glucose	38	41.512	0.28	0.109	-0.023	0.652	0.189
	Lactate	16	24.391	0.059	0.385	-0.017	0.895	0.66
	Pyruvate	60	63.579	0.318	0.072	-0.027	0.453	0.949
**Cofactors and vitamins**								
	Biliverdin	9	2.331	0.969	0	0	0.756	0.941
	Bilirubin (Z,Z)	8	9.412	0.224	0.256	0	0.251	0.514
	Bilirubin (E,E)	7	4.709	0.582	0	0	0.242	0.861
	Bilirubin (E,Z or Z,E)	4	0.368	0.947	0	0	0.976	0.925
	Acetate	20	18.887	0.464	0	0.071	0.368	0.802
**Energy**								
	Succinylcarnitine	10	4.933	0.84	0	0	0.827	0.331
	Acetone	19	19.26	0.376	0.065	0.024	0.743	0.377
	Citrate	80	97.043	0.082	0.186	-0.064	0.03	0.71
**Lipid**								
	Arachidonate (20:4n6)	5	2.17	0.705	0	0	0.737	0.396
	Carnitine	21	9.239	0.98	0	0	0.608	0.426

	2-hydroxyisobutyrate	4	2.725	0.436	0	0	0.934	0.549
	Androsterone sulfate	8	6.062	0.533	0	0	0.662	0.472
	Hexanoylcarnitine	9	6.711	0.568	0	0	0.479	0.398
	Butyrylcarnitine	25	14.263	0.94	0	0	0.789	0.463
	Propionylcarnitine	5	3.431	0.489	0	0	0.322	0.809
	10-undecenoate (11:1n1)	4	1.565	0.667	0	0	0.911	0.966
	3-dehydrocarnitine	6	1.104	0.954	0	0	0.592	0.556
	1-arachidonoylglycerophosphocholine	5	1.559	0.816	0	0	0.607	0.699
	Octanoylcarnitine	7	2.541	0.864	0	0	0.905	0.923
	Decanoylcarnitine	5	0.676	0.954	0	0	0.831	0.482
	Epiandrosterone sulfate	7	12.201	0.058	0.508	0	0.812	0.307
	1-arachidonoylglycerophosphoinositol	5	3.164	0.531	0	0	0.604	0.868
	1-arachidonoylglycerophosphoethanolamine	4	1.902	0.593	0	0	0.408	0.86
	Tetradecanedioate	4	0.533	0.912	0	0	0.59	0.547
	Hexadecanedioate	5	3.785	0.436	0	0	0.746	0.556
	Dihomo-linolenate (20:3n3 or n6)	3	6.266	0.044	0.681	0.001	0.297	
	Octadecanedioate	4	2.754	0.431	0	0	0.506	0.727
	5alpha-androstan-3beta,17beta-diol disulfate	6	5.436	0.365	0.08	0	0.95	0.779
	4-androsten-3beta,17beta-diol disulfate 1	6	3.831	0.574	0	0	0.697	0.97
	Cis-4-decenoyl carnitine	5	1.85	0.763	0	0	0.689	0.512
	22:6, docosahexaenoic acid	6	1.83	0.872	0	0.004	0.988	<0.001
	Acetoacetate	9	10.458	0.234	0.235	-0.027	0.876	0.664
	Apolipoprotein A1	239	246.641	0.337	0.035	-0.007	0.669	0.358
	Apolipoprotein B	167	144.199	0.888	0	-0.03	0.075	0.516
	3-Hydroxybutyrate	25	29.139	0.215	0.176	0.01	0.901	0.691

	Total cholines	181	178.295	0.522	0	0.018	0.362	0.605
	Docosahexaenoic acid	161	197.931	0.022	0.192	0.026	0.192	0.327
	Glycoprotein acetyls	171	139.158	0.96	0	0.02	0.313	0.541
	HDL cholesterol	280	314.336	0.071	0.112	0.007	0.634	0.076
	Linoleic acid	179	180.194	0.44	0.012	0.004	0.86	0.729
	LDL cholesterol	153	130.997	0.89	0	-0.012	0.497	0.954
	Monounsaturated fatty acids	212	217.244	0.369	0.029	0.006	0.738	0.847
	Phosphatidylcholines	191	183.486	0.619	0	0.014	0.437	0.9
	Phosphoglycerides	176	167.982	0.635	0	0.021	0.279	0.232
	Polyunsaturated fatty acids	222	236.06	0.232	0.064	0.025	0.17	0.896
	Saturated fatty acids	158	164.414	0.327	0.045	-0.006	0.765	0.56
	Sphingomyelins	189	201.589	0.236	0.067	-0.026	0.135	0.381
	Total cholesterol	165	154.262	0.696	0	-0.017	0.336	0.201
	Total esterified cholesterol	169	164.713	0.557	0	-0.018	0.312	0.608
	Total fatty acids	195	212.652	0.171	0.088	-0.005	0.799	0.338
	Total free cholesterol	170	157.613	0.725	0	-0.017	0.321	0.054
	Total triglycerides	240	225.02	0.733	0	-0.001	0.945	0.025
	VLDL cholesterol	188	171.14	0.791	0	0.011	0.566	0.34
**Nucleotide**								
	Uridine	3	3.352	0.187	0.403	0.001	0.319	
	Urate	5	5.967	0.202	0.33	0	0.202	0.24
**Peptide**								
	Gamma-glutamyltyrosine	5	5.586	0.232	0.284	-0.001	0.177	0.507
	N-acetylornithine	10	6.438	0.695	0	0	0.203	0.549
	HWESASXX	3	0.97	0.616	0	0	0.988	

	Bradykinin, des-arg(9)	5	1.665	0.797	0	0	0.922	0.513
	Glycoproteins	82	124.398	0.001	0.349	-0.042	0.229	0.878
	Albumin	48	32.699	0.944	0	-0.007	0.835	0.2
**Unknown**								
	X-03094	5	3.108	0.54	0	0	0.275	0.113
	X-18601	3	1.939	0.379	0	0	0.926	
	X-08402	6	10.077	0.073	0.504	0	0.339	0.606
	X-08988	3	2.266	0.322	0.117	0	0.609	
	X-10510	3	0.106	0.948	0	0	0.802	
	X-11204	3	1.072	0.585	0	0.001	0.773	
	X-02269	4	1.845	0.605	0	0	0.825	0.286
	X-11261	6	1.678	0.892	0	0	0.6	0.252
	X-11315	3	0.798	0.671	0	0	0.557	
	X-03056	8	2.15	0.951	0	0	0.668	0.434
	X-09789	3	0.254	0.881	0	0	0.75	
	X-11440	6	4.813	0.439	0	0	0.878	0.822
	X-11441	6	3.631	0.604	0	0	0.479	0.53
	X-11442	7	3.598	0.731	0	0	0.79	0.953
	X-11444	5	3.883	0.422	0	0.001	0.146	0.97
	X-11445	3	2.692	0.26	0.257	0.001	0.667	
	X-11469	5	1.564	0.815	0	0	0.735	0.578
	X-11491	4	1.325	0.723	0	0	0.967	0.778
	X-11529	11	11.221	0.341	0.109	0	0.112	0.618
	X-11530	8	5.589	0.589	0	0	0.394	0.975
	X-11538	8	7.26	0.402	0.036	0	0.305	0.617

	X-11593--O-methylascorbate	13	11.182	0.513	0	0	0.591	0.974
	X-11787	8	3.688	0.815	0	0	0.826	0.469
	X-11792	3	0.965	0.617	0	0	0.551	
	X-11793--oxidized bilirubin	10	9.076	0.43	0.008	0	0.454	0.568
	X-11905	3	1.835	0.4	0	0	0.43	
	X-12063	15	21.289	0.094	0.342	0	0.294	0.873
	X-12092	19	8.462	0.971	0	0	0.245	0.913
	X-12093	5	3.906	0.419	0	0	0.424	0.688
	X-12244--N-acetylcarnosine	6	3.627	0.604	0	0.001	0.216	0.316
	X-12456	3	3.644	0.162	0.451	-0.001	0.364	
	X-12510--2-aminooctanoic acid	7	7.372	0.288	0.186	0	0.774	0.933
	X-12556	4	4.333	0.228	0.308	0	0.923	0.323
	X-12644	3	1.062	0.588	0	0	0.788	
	X-12696	5	0.875	0.928	0	0	0.767	0.958
	X-12728	8	7.315	0.397	0.043	0	0.648	0.893
	X-12798	13	16.665	0.163	0.28	0	0.952	0.797
	X-12844	4	2.572	0.462	0	0	0.682	0.992
	X-12850	3	4.128	0.127	0.515	0	0.592	
	X-13429	4	3.358	0.34	0.107	0	0.216	0.83
	X-13431--nonanoylcarnitine	7	1.636	0.95	0	0	0.674	0.846
	X-13435	3	2.134	0.344	0.063	0	0.47	
	X-14205--alpha-glutamyltyrosine	3	2.333	0.311	0.143	0	0.607	
	X-14626	3	0.878	0.645	0	0	0.719	

Supplementary Table 4D. Sensitivity analyses in gastric cancer

Category	Exposures	No.of SNPs	Inverse variance weighting			MR-Egger		MR-PRESSO
			Q	Q *P*-val	I^2	intercept	intercept *P*-val	Global Test *P*-val
**Amino acid**								
	Tryptophan	18	17.879	0.396	0.049	-0.001	0.249	0.905
	4-acetamidobutanoate	6	2.312	0.804	0	0	0.905	0.984
	Proline	4	2.107	0.551	0	0	0.633	0.852
	Citrulline	4	0.826	0.843	0	0	0.901	0.383
	Betaine	5	4.061	0.398	0.015	-0.001	0.205	0.835
	Kynurenine	6	2.439	0.786	0	0	0.698	0.657
	3-methyl-2-oxovalerate	3	0.748	0.688	0	0	0.737	
	N-acetylglycine	7	5.171	0.522	0	0	0.339	0.856
	Serine	3	3.916	0.141	0.489	-0.001	0.764	
	Pyroglutamine	5	2.627	0.622	0	0	0.627	0.454
	Isobutyrylcarnitine	8	6.202	0.516	0	0	0.194	0.856
	Alpha-hydroxyisovalerate	3	2.756	0.252	0.274	0	0.755	
	Asparagine	3	4.821	0.09	0.585	0	0.624	
	Isovalerylcarnitine	7	3.436	0.752	0	0	0.744	0.875
	Glutaroyl carnitine	11	10.444	0.402	0.043	0	0.658	0.459
	Tryptophan betaine	4	2.355	0.502	0	0.001	0.278	0.088
	Alanine	52	41.763	0.818	0	0.004	0.884	0.632
	Creatinine	91	92.889	0.396	0.031	0.004	0.862	0.182
	Glutamine	98	100.377	0.387	0.034	0.019	0.16	0.127
	Glycine	221	201.026	0.816	0	-0.002	0.833	0.269
	Histidine	45	32.89	0.89	0	-0.001	0.965	0.016

	Isoleucine	18	14.877	0.604	0	0.002	0.972	0.023
	Leucine	34	29.695	0.632	0	0.013	0.611	0.818
	Phenylalanine	33	29.337	0.602	0	0.019	0.563	0.646
	Tyrosine	76	62.207	0.854	0	0.022	0.188	0.565
	Valine	49	43.592	0.654	0	-0.004	0.854	0.922
**Carbohydrate**								
	Mannose	6	1.289	0.936	0	0	0.477	0.687
	1,5-anhydroglucitol (1,5-AG)	6	3.524	0.62	0	0	0.573	0.767
	Erythronate	3	0.184	0.912	0	0	0.782	
	Glucose	38	33.619	0.628	0	0.052	0.08	0.326
	Lactate	16	14.955	0.455	0	-0.011	0.856	0.422
	Pyruvate	60	65.179	0.271	0.095	-0.006	0.803	0.504
**Cofactors and vitamins**								
	Biliverdin	9	4.716	0.787	0	0	0.788	0.81
	Bilirubin (Z,Z)	8	2.322	0.94	0	0	0.836	0.32
	Bilirubin (E,E)	7	1.568	0.955	0	0	0.887	0.763
	Bilirubin (E,Z or Z,E)	4	0.787	0.852	0	0	0.922	0.691
	Acetate	20	10.801	0.93	0	-0.019	0.689	0.408
**Energy**								
	Succinylcarnitine	10	5.246	0.812	0	0	0.347	0.502
	Acetone	19	16.917	0.529	0	-0.045	0.293	0.607
	Citrate	80	80.332	0.437	0.017	0.037	0.027	0.703
**Lipid**								
	Arachidonate (20:4n6)	5	4.424	0.352	0.096	0	0.951	0.86
	Carnitine	21	11.74	0.925	0	0	0.261	0.668

	2-hydroxyisobutyrate	4	2.897	0.408	0	0	0.311	0.805
	Androsterone sulfate	8	8.981	0.254	0.221	0	0.048	0.48
	Hexanoylcarnitine	9	4.874	0.771	0	0	0.808	0.588
	Butyrylcarnitine	25	11.401	0.986	0	0	0.604	0.696
	Propionylcarnitine	5	0.352	0.986	0	0	0.959	0.816
	10-undecenoate (11:1n1)	4	0.99	0.804	0	0	0.868	0.761
	3-dehydrocarnitine	6	3.248	0.662	0	0	0.893	0.543
	1-arachidonoylglycerophosphocholine	5	5.506	0.239	0.274	0	0.657	0.866
	Octanoylcarnitine	7	3.482	0.746	0	0	0.317	0.618
	Decanoylcarnitine	5	1.561	0.816	0	0	0.646	0.333
	Epiandrosterone sulfate	7	7.621	0.267	0.213	0	0.076	0.606
	1-arachidonoylglycerophosphoinositol	5	3.252	0.517	0	0	0.313	0.827
	1-arachidonoylglycerophosphoethanolamine	4	5.534	0.137	0.458	0	0.467	0.414
	Tetradecanedioate	4	2.325	0.508	0	0	0.464	0.966
	Hexadecanedioate	5	3.031	0.553	0	0	0.521	0.818
	Dihomo-linolenate (20:3n3 or n6)	3	0.008	0.996	0	0	0.961	
	Octadecanedioate	4	3.441	0.329	0.128	0	0.752	0.942
	5alpha-androstan-3beta,17beta-diol disulfate	6	3.365	0.644	0	0	0.596	0.866
	4-androsten-3beta,17beta-diol disulfate 1	6	2.45	0.784	0	0	0.791	0.935
	Cis-4-decenoyl carnitine	5	0.543	0.969	0	0	0.72	0.973
	22:6, docosahexaenoic acid	6	6.087	0.298	0.179	0.237	0.147	0.727
	Acetoacetate	9	7.13	0.523	0	-0.099	0.275	0.675
	Apolipoprotein A1	239	261.695	0.14	0.091	-0.001	0.914	0.453
	Apolipoprotein B	167	159.343	0.631	0	-0.011	0.296	0.681
	3-Hydroxybutyrate	25	20.008	0.696	0	-0.014	0.73	0.08

	Total cholines	181	190.503	0.282	0.055	0.011	0.382	0.873
	Docosahexaenoic acid	161	143.127	0.827	0	0.013	0.231	0.292
	Glycoprotein acetyls	171	154.065	0.804	0	-0.023	0.059	0.048
	HDL cholesterol	280	332.891	0.015	0.162	-0.001	0.895	0.433
	Linoleic acid	179	205.514	0.077	0.134	0.024	0.066	0.556
	LDL cholesterol	153	142.612	0.695	0	-0.005	0.633	0.78
	Monounsaturated fatty acids	212	188.045	0.87	0	-0.013	0.212	0.864
	Phosphatidylcholines	191	202.503	0.254	0.062	0.015	0.193	0.619
	Phosphoglycerides	176	176.264	0.459	0.007	0.017	0.139	0.01
	Polyunsaturated fatty acids	222	229.735	0.329	0.038	0.014	0.209	0.705
	Saturated fatty acids	158	149.407	0.655	0	0.004	0.745	0.81
	Sphingomyelins	189	241.689	0.005	0.222	-0.006	0.603	0.885
	Total cholesterol	165	181.628	0.164	0.097	0.004	0.704	0.71
	Total esterified cholesterol	169	198.566	0.054	0.154	-0.004	0.756	0.635
	Total fatty acids	195	182.727	0.709	0	-0.004	0.746	0.304
	Total free cholesterol	170	179.113	0.282	0.056	-0.001	0.902	0.479
	Total triglycerides	239	234.202	0.557	0	-0.002	0.824	0.805
	VLDL cholesterol	188	231.447	0.015	0.192	0.006	0.655	0.517
**Nucleotide**								
	Uridine	3	0.597	0.742	0	0	0.605	
	Urate	5	3.021	0.554	0	0	0.727	0.292
**Peptide**								
	Gamma-glutamyltyrosine	5	0.407	0.982	0	0	0.802	0.921
	N-acetylornithine	10	6.151	0.725	0	0	0.691	0.318
	HWESASXX	3	0.982	0.612	0	0	0.537	

	Bradykinin, des-arg(9)	5	1.973	0.741	0	0	0.978	0.402
	Glycoproteins	82	72.809	0.73	0	0.003	0.851	0.347
	Albumin	48	47.141	0.467	0.003	-0.006	0.763	0.253
**Unknown**								
	X-03094	5	4.361	0.359	0.083	0	0.445	0.305
	X-18601	3	0.265	0.876	0	0	0.724	
	X-08402	6	8.182	0.146	0.389	0	0.476	0.785
	X-08988	3	0.72	0.698	0	0	0.915	
	X-10510	3	1.805	0.406	0	0	0.493	
	X-11204	3	1.46	0.482	0	-0.002	0.822	
	X-02269	4	1.935	0.586	0	0.001	0.524	0.62
	X-11261	6	0.79	0.978	0	0	0.598	0.908
	X-11315	3	4.372	0.112	0.542	0.001	0.556	
	X-03056	8	2.871	0.897	0	0	0.881	0.48
	X-09789	3	0.439	0.803	0	0	0.927	
	X-11440	6	3.017	0.697	0	0	0.689	0.515
	X-11441	6	2.738	0.74	0	0	0.862	0.541
	X-11442	7	1.952	0.924	0	0	0.814	0.993
	X-11444	5	1.503	0.826	0	-0.001	0.395	0.45
	X-11445	3	0.094	0.954	0	0	0.864	
	X-11469	5	1.673	0.796	0	0	0.726	0.463
	X-11491	4	2.42	0.49	0	0	0.564	0.826
	X-11529	11	9.346	0.5	0	0	0.448	0.924
	X-11530	8	1.53	0.981	0	0	0.786	0.942
	X-11538	8	4.006	0.779	0	0	0.369	0.611

	X-11593--O-methylascorbate	13	8.783	0.721	0	0	0.813	0.612
	X-11787	8	2.78	0.905	0	0	0.761	0.298
	X-11792	3	0.052	0.974	0	0	0.944	
	X-11793--oxidized bilirubin	10	3.6	0.936	0	0	0.686	0.147
	X-11905	3	3.615	0.164	0.447	0	0.611	
	X-12063	15	12.034	0.604	0	0	0.076	0.976
	X-12092	19	14.553	0.692	0	0	0.687	0.605
	X-12093	5	3.005	0.557	0	0	0.572	0.723
	X-12244--N-acetylcarnosine	6	11.194	0.048	0.553	-0.001	0.213	0.986
	X-12456	3	3.334	0.189	0.4	-0.001	0.623	
	X-12510--2-aminooctanoic acid	7	8.366	0.213	0.283	0	0.244	0.57
	X-12556	4	2.907	0.406	0	0	0.774	0.976
	X-12644	3	0.572	0.751	0	-0.001	0.588	
	X-12696	5	3.91	0.418	0	0	0.302	0.807
	X-12728	8	8.494	0.291	0.176	0	0.942	0.328
	X-12798	13	6.932	0.862	0	0	0.995	0.526
	X-12844	4	7.556	0.056	0.603	0	0.876	0.435
	X-12850	3	0.583	0.747	0	0	0.711	
	X-13429	4	2.791	0.425	0	0	0.796	0.912
	X-13431--nonanoylcarnitine	7	3.663	0.722	0	0	0.751	0.767
	X-13435	3	3.293	0.193	0.393	0	0.806	
	X-14205--alpha-glutamyltyrosine	3	3.034	0.219	0.341	0	0.4	
	X-14626	3	4.533	0.104	0.559	-0.001	0.281	

Supplementary Table 4E. Sensitivity analyses in pancreatic cancer

Category	Exposures	No.of SNPs	Inverse variance weighting			MR-Egger		MR-PRESSO
			Q	Q *P*-val	I^2	intercept	intercept *P*-val	Global Test *P*-val
**Amino acid**								
	Tryptophan	18	9.912	0.907	0	0	0.682	0.564
	4-acetamidobutanoate	6	5.86	0.32	0.147	0	0.57	0.448
	Proline	4	1.167	0.761	0	0	0.523	0.743
	Citrulline	4	1.316	0.725	0	0.001	0.802	0.912
	Betaine	5	1.502	0.826	0	0	0.755	0.354
	Kynurenine	6	2.333	0.801	0	0	0.874	0.905
	3-methyl-2-oxovalerate	3	0.465	0.792	0	0	0.906	
	N-acetylglycine	7	4.492	0.61	0	0	0.171	0.432
	Serine	3	0.685	0.71	0	-0.001	0.723	
	Pyroglutamine	5	5.812	0.214	0.312	0	0.209	0.738
	Isobutyrylcarnitine	8	3.809	0.801	0	0	0.785	0.517
	Alpha-hydroxyisovalerate	3	1.858	0.395	0	0	0.514	
	Asparagine	3	3.498	0.174	0.428	0.001	0.323	
	Isovalerylcarnitine	7	8.544	0.201	0.298	0.001	0.44	0.923
	Glutaroyl carnitine	11	7.07	0.719	0	0	0.724	0.483
	Tryptophan betaine	4	3.054	0.383	0.018	0.001	0.352	0.028
	Alanine	52	45.199	0.702	0	-0.045	0.128	0.745
	Creatinine	91	87.569	0.553	0	-0.012	0.572	0.992
	Glutamine	98	98.096	0.45	0.011	-0.016	0.241	0.994
	Glycine	221	194.017	0.896	0	-0.007	0.427	0.844
	Histidine	45	38.413	0.709	0	0.002	0.922	0.998

	Isoleucine	18	13.511	0.701	0	-0.008	0.869	0.362
	Leucine	34	28.073	0.711	0	-0.03	0.265	0.893
	Phenylalanine	33	46.46	0.047	0.311	-0.022	0.594	0.283
	Tyrosine	76	67.056	0.732	0	-0.003	0.836	0.917
	Valine	49	36.973	0.876	0	-0.01	0.671	0.271
**Carbohydrate**								
	Mannose	6	1.585	0.903	0	0	0.714	0.445
	1,5-anhydroglucitol (1,5-AG)	6	7.574	0.181	0.34	0.001	0.129	0.66
	Erythronate	3	6.745	0.034	0.703	0	0.997	
	Glucose	38	42.606	0.243	0.132	0.042	0.187	0.983
	Lactate	16	10.574	0.782	0	-0.075	0.239	0.494
	Pyruvate	60	78.415	0.046	0.248	0.053	0.032	0.984
**Cofactors and vitamins**								
	Biliverdin	9	2.853	0.943	0	0	0.963	0.952
	Bilirubin (Z,Z)	8	3.804	0.802	0	0	0.662	0.893
	Bilirubin (E,E)	7	2.282	0.892	0	0	0.835	0.719
	Bilirubin (E,Z or Z,E)	4	0.485	0.922	0	0	0.66	0.25
	Acetate	20	21.602	0.305	0.12	0.008	0.873	0.552
**Energy**								
	Succinylcarnitine	10	7.156	0.621	0	0	0.787	0.289
	Acetone	19	10.92	0.898	0	-0.005	0.913	0.72
	Citrate	80	62.125	0.919	0	-0.008	0.637	0.555
**Lipid**								
	Arachidonate (20:4n6)	5	4.737	0.315	0.156	0	0.973	0.752
	Carnitine	21	16.63	0.677	0	0	0.164	0.637

	2-hydroxyisobutyrate	4	2.507	0.474	0	0	0.27	0.905
	Androsterone sulfate	8	13.65	0.058	0.487	0	0.185	0.677
	Hexanoylcarnitine	9	5.661	0.685	0	0	0.475	0.839
	Butyrylcarnitine	25	15.534	0.904	0	0	0.803	0.218
	Propionylcarnitine	5	3.813	0.432	0	0	0.868	0.983
	10-undecenoate (11:1n1)	4	3.489	0.322	0.14	0	0.368	0.19
	3-dehydrocarnitine	6	4.998	0.416	0	0.001	0.207	0.9
	1-arachidonoylglycerophosphocholine	5	3.301	0.509	0	0	0.505	0.929
	Octanoylcarnitine	7	3.334	0.766	0	0	0.301	0.739
	Decanoylcarnitine	5	3.328	0.504	0	0	0.357	0.406
	Epiandrosterone sulfate	7	13.538	0.035	0.557	0	0.234	0.498
	1-arachidonoylglycerophosphoinositol	5	0.167	0.997	0	0	0.754	1
	1-arachidonoylglycerophosphoethanolamine	4	0.675	0.879	0	0	0.517	0.479
	Tetradecanedioate	4	0.759	0.859	0	0	0.791	0.959
	Hexadecanedioate	5	0.958	0.916	0	0	0.793	0.904
	Dihomo-linolenate (20:3n3 or n6)	3	2.077	0.354	0.037	-0.001	0.432	
	Octadecanedioate	4	1.432	0.698	0	0	0.825	0.866
	5alpha-androstan-3beta,17beta-diol disulfate	6	3.247	0.662	0	0	0.552	0.415
	4-androsten-3beta,17beta-diol disulfate 1	6	1.435	0.92	0	0	0.819	0.913
	Cis-4-decenoyl carnitine	5	2.869	0.58	0	-0.001	0.207	0.925
	22:6, docosahexaenoic acid	6	4.02	0.547	0	-0.09	0.54	0.48
	Acetoacetate	9	3.293	0.915	0	0.019	0.832	0.89
	Apolipoprotein A1	239	194.066	0.983	0	0.005	0.645	0.851
	Apolipoprotein B	167	175.83	0.286	0.056	-0.014	0.205	0.905
	3-Hydroxybutyrate	25	18.556	0.775	0	0.006	0.882	0.734

	Total cholines	181	160.56	0.848	0	0.01	0.418	0.979
	Docosahexaenoic acid	161	141.814	0.846	0	-0.001	0.924	0.928
	Glycoprotein acetyls	171	180.259	0.28	0.057	0.04	0.002	0.617
	HDL cholesterol	280	213.009	0.999	0	0.002	0.817	0.922
	Linoleic acid	179	154.404	0.899	0	0.007	0.552	0.96
	LDL cholesterol	153	165.775	0.21	0.083	-0.005	0.629	0.281
	Monounsaturated fatty acids	212	171.947	0.977	0	0.004	0.742	0.74
	Phosphatidylcholines	191	169.886	0.85	0	0.014	0.225	0.219
	Phosphoglycerides	176	156.538	0.838	0	0.009	0.446	0.562
	Polyunsaturated fatty acids	222	192.94	0.914	0	0.012	0.285	0.288
	Saturated fatty acids	158	119.221	0.989	0	0.002	0.888	0.693
	Sphingomyelins	189	183.42	0.581	0	-0.011	0.287	0.72
	Total cholesterol	165	152.357	0.733	0	-0.015	0.16	0.803
	Total esterified cholesterol	169	160.895	0.639	0	-0.014	0.187	0.065
	Total fatty acids	195	152.711	0.987	0	0.008	0.492	0.049
	Total free cholesterol	170	165.281	0.567	0	-0.014	0.188	0.811
	Total triglycerides	240	202.632	0.958	0	0	0.966	0.847
	VLDL cholesterol	188	193.103	0.364	0.032	0.008	0.531	0.921
**Nucleotide**								
	Uridine	3	6.43	0.04	0.689	-0.001	0.634	
	Urate	5	1.369	0.849	0	0	0.608	0.752
**Peptide**								
	Gamma-glutamyltyrosine	5	6.429	0.169	0.378	-0.001	0.697	0.762
	N-acetylornithine	10	6.133	0.727	0	0	0.879	0.224
	HWESASXX	3	1.985	0.371	0	0	0.522	

	Bradykinin, des-arg(9)	5	0.174	0.996	0	0	0.955	0.716
	Glycoproteins	82	81.614	0.46	0.008	-0.019	0.285	0.616
	Albumin	48	28.14	0.987	0	-0.026	0.218	0.817
**Unknown**								
	X-03094	5	1.801	0.772	0	0	0.807	0.126
	X-18601	3	1.937	0.38	0	-0.001	0.61	
	X-08402	6	2.959	0.706	0	0	0.467	0.73
	X-08988	3	0.048	0.976	0	0	0.894	
	X-10510	3	2.017	0.365	0.009	0	0.391	
	X-11204	3	0.944	0.624	0	-0.003	0.717	
	X-02269	4	0.178	0.981	0	0	0.861	0.811
	X-11261	6	2.866	0.721	0	0	0.545	0.536
	X-11315	3	0.218	0.897	0	0	0.78	
	X-03056	8	5.645	0.582	0	0	0.765	0.565
	X-09789	3	2.069	0.355	0.033	0	0.858	
	X-11440	6	1.51	0.912	0	0	0.467	0.339
	X-11441	6	1.975	0.853	0	0	0.956	0.995
	X-11442	7	2.386	0.881	0	0	0.975	0.883
	X-11444	5	4.863	0.302	0.178	0	0.47	0.721
	X-11445	3	0.875	0.646	0	0	0.842	
	X-11469	5	0.361	0.986	0	0	0.951	0.462
	X-11491	4	4.878	0.181	0.385	0	0.656	0.678
	X-11529	11	15.942	0.101	0.373	0	0.69	0.754
	X-11530	8	2.104	0.954	0	0	0.87	0.679
	X-11538	8	3.556	0.829	0	0	0.384	0.981

	X-11593--O-methylascorbate	13	9.485	0.661	0	0	0.276	0.283
	X-11787	8	6.782	0.452	0	0	0.454	0.39
	X-11792	3	0.018	0.991	0	0	0.997	
	X-11793--oxidized bilirubin	10	4.876	0.845	0	0	0.564	0.719
	X-11905	3	2.565	0.277	0.22	0.001	0.356	
	X-12063	15	7.53	0.912	0	0	0.102	0.656
	X-12092	19	24.195	0.149	0.256	0	0.745	0.767
	X-12093	5	2.339	0.674	0	0	0.355	0.296
	X-12244--N-acetylcarnosine	6	14.202	0.014	0.648	0	0.689	0.219
	X-12456	3	0.891	0.641	0	0.001	0.53	
	X-12510--2-aminooctanoic acid	7	7.083	0.313	0.153	0	0.22	0.49
	X-12556	4	4.862	0.182	0.383	-0.002	0.192	0.805
	X-12644	3	3.193	0.203	0.374	0.002	0.325	
	X-12696	5	3.934	0.415	0	0	0.404	0.65
	X-12728	8	7.614	0.368	0.081	0	0.885	0.784
	X-12798	13	12.225	0.428	0.018	0	0.59	0.429
	X-12844	4	1.556	0.669	0	0	0.596	0.829
	X-12850	3	0.099	0.952	0	0	0.819	
	X-13429	4	1.675	0.643	0	0	0.354	0.544
	X-13431--nonanoylcarnitine	7	5.214	0.517	0	0	0.615	0.717
	X-13435	3	4.873	0.087	0.59	-0.001	0.628	
	X-14205--alpha-glutamyltyrosine	3	0.121	0.941	0	0	0.908	
	X-14626	3	0.086	0.958	0	0	0.83	
