# Supplementary material for: Association between human blood metabolome and the risk of gastrointestinal tumors
Source: PLoS One. 2024 May 30;19(5):e0304574. doi: 10.1371/journal.pone.0304574 (PMC11139295; doi:10.1371/journal.pone.0304574)
Supplement: S5 Table — (PDF) [file pone.0304574.s005.pdf]

Supplementary Table 5. Results of Phe-MR (Isovalerylcarnitine and 118 diseases)

Outcomes	No.of SNPs	Inverse variance weighting						MR-Egger					Weighted Median			MR-PRESSO
		OR/Beta	95% CI	*P*-val	Q	Q *P*-val	I^2	OR/Beta	95% CI	*P*-val	intercept	intercept *P*-val	OR/Beta	95% CI	*P*-val	Global Test *P*-val
G43.9 Migraine, unspecified	3	1.002	(0.999,1.006)	0.218	1.011	0.603	0	1.002	(0.985,1.02)	0.859	0	0.982	1.002	0.998,1.006)	0.442	
G45.9 Transient cerebral ischaemic attack, unspecified	3	0.997	(0.993,1.002)	0.261	2.311	0.315	0.135	1	(0.97,1.031)	0.998	0	0.891	0.995	(0.99,1)	0.061	
G47.3 Sleep apnoea	3	1.003	(0.997,1.01)	0.353	2.966	0.227	0.326	0.99	(0.954,1.028)	0.703	0	0.619	1.003	(0.997,1.01)	0.352	
G56.0 Carpal tunnel syndrome	6	1.002	(0.991,1.012)	0.753	7.557	0.182	0.338	1.033	(0.973,1.097)	0.344	-0.001	0.36	1.002	(0.991,1.014)	0.667	0.255
H00.1 Chalazion	3	0.994	(0.984,1.004)	0.232	10.145	0.006	0.803	1.012	(0.954,1.073)	0.765	-0.001	0.655	0.994	(0.988,1)	0.055	
H02.8 Other specified disorders of eyelid	3	1	(0.995,1.004)	0.904	0.771	0.68	0	1.009	(0.987,1.033)	0.567	0	0.55	1	(0.995,1.005)	0.925	
H25.0 Senile incipient cataract	3	0.997	(0.993,1.002)	0.276	3.233	0.199	0.381	1.006	(0.979,1.034)	0.742	0	0.642	0.997	(0.993,1.002)	0.234	
H25.1 Senile nuclear cataract	3	0.999	(0.994,1.004)	0.592	2.063	0.356	0.031	1.006	(0.974,1.038)	0.786	0	0.734	0.998	(0.992,1.004)	0.572	
H26.9 Cataract, unspecified	6	0.996	(0.988,1.005)	0.439	3.706	0.592	0	0.996	(0.947,1.048)	0.894	0	0.995	0.993	(0.982,1.004)	0.214	0.653
I20.0 Unstable angina	6	1.003	(0.997,1.009)	0.286	2.628	0.757	0	0.985	(0.953,1.018)	0.422	0.001	0.334	1.001	(0.994,1.009)	0.686	0.774
I20.9 Angina pectoris, unspecified	6	1	(0.993,1.006)	0.879	2.416	0.789	0	0.991	(0.957,1.027)	0.648	0	0.661	1.001	(0.994,1.009)	0.759	0.707
I21.0 Acute transmural myocardial infarction of anterior wall	3	0.998	(0.994,1.002)	0.317	0.584	0.747	0	0.999	(0.979,1.019)	0.928	0	0.94	0.998	(0.994,1.003)	0.435	
I21.1 Acute transmural myocardial infarction of inferior wall	3	1.004	(0.999,1.009)	0.098	0.566	0.754	0	1.001	(0.979,1.024)	0.943	0	0.841	1.004	(0.999,1.009)	0.11	
I21.9 Acute myocardial infarction, unspecified	3	1.002	(0.997,1.008)	0.456	0.403	0.817	0	1.009	(0.983,1.036)	0.617	0	0.682	1.002	(0.996,1.008)	0.486	
I25.1 Atherosclerotic heart disease	7	1.007	(0.996,1.019)	0.184	7.237	0.299	0.171	0.989	(0.931,1.052)	0.743	0.001	0.578	1.01	(0.996,1.023)	0.167	0.299
I25.9 Chronic ischaemic heart disease, unspecified	3	0.998	(0.994,1.002)	0.25	1.275	0.529	0	1.005	(0.986,1.024)	0.703	0	0.588	0.998	(0.994,1.002)	0.337	
I26.9 Pulmonary embolism without mention of acute cor pulmonale	3	0.999	(0.994,1.003)	0.55	0.315	0.854	0	1.003	(0.979,1.026)	0.868	0	0.791	0.999	(0.993,1.004)	0.597	
I47.1 Supraventricular tachycardia	3	0.999	(0.995,1.004)	0.807	0.075	0.963	0	1.002	(0.982,1.022)	0.877	0	0.843	1	(0.995,1.004)	0.878	
I48 Atrial fibrillation and flutter	6	1.001	(0.993,1.01)	0.778	7.221	0.205	0.308	0.976	(0.93,1.025)	0.383	0.001	0.355	0.998	(0.99,1.007)	0.703	0.281
I63.9 Cerebral infarction, unspecified	3	0.997	(0.99,1.005)	0.476	6.194	0.045	0.677	1.013	(0.973,1.055)	0.635	-0.001	0.572	0.997	(0.991,1.002)	0.233	
I80.2 Phlebitis and thrombophlebitis of other deep vessels of lower extremities	3	1	(0.995,1.005)	0.986	0.111	0.946	0	0.997	(0.974,1.021)	0.84	0	0.839	1	(0.995,1.005)	0.948	
I83.9 Varicose veins of lower extremities without ulcer or inflammation	7	1.003	(0.986,1.021)	0.733	21.723	0.001	0.724	0.976	(0.885,1.075)	0.64	0.001	0.595	1.008	(0.992,1.023)	0.347	0.014
I84.1 Internal haemorrhoids with other complications	3	1	(0.99,1.011)	0.957	10.597	0.005	0.811	1.019	(0.96,1.081)	0.652	-0.001	0.65	1	(0.995,1.006)	0.974	
I84.2 Internal haemorrhoids without complication	3	1.001	(0.994,1.009)	0.705	6.487	0.039	0.692	1.008	(0.958,1.062)	0.803	0	0.833	1	(0.995,1.006)	0.916	
I84.6 Residual haemorrhoidal skin tags	3	0.995	(0.991,0.999)	0.021	0.643	0.725	0	0.997	(0.977,1.017)	0.794	0	0.909	0.995	(0.991,1)	0.04	
I84.8 Unspecified haemorrhoids with other complications	3	1	(0.993,1.006)	0.943	2.743	0.254	0.271	0.993	(0.953,1.035)	0.801	0	0.804	1.001	(0.994,1.007)	0.866	
I84.9 Unspecified haemorrhoids without complication	6	0.99	(0.983,0.997)	0.009	2.639	0.755	0	1.005	(0.963,1.049)	0.819	0	0.513	0.99	(0.981,0.999)	0.031	0.81
J18.1 Lobar pneumonia, unspecified	3	1.001	(0.996,1.007)	0.68	1.511	0.47	0	0.993	(0.965,1.02)	0.689	0	0.644	1.002	(0.995,1.008)	0.58	

J18.9 Pneumonia, unspecified	3	0.996	(0.991,1)	0.058	1.004	0.605	0	0.996	(0.975,1.018)	0.789	0	0.97	0.996	(0.991,1.001)	0.152	
J22 Unspecified acute lower respiratory infection	5	0.998	(0.992,1.003)	0.395	1.087	0.896	0	0.989	(0.959,1.02)	0.527	0	0.609	0.997	(0.99,1.003)	0.327	0.922
J33.9 Nasal polyp, unspecified	3	1.008	(1,1.016)	0.057	7.728	0.021	0.741	0.998	(0.947,1.051)	0.94	0	0.757	1.008	(1.003,1.014)	0.003	
J34.2 Deviated nasal septum	6	1.003	(0.998,1.009)	0.232	2.135	0.83	0	0.999	(0.969,1.03)	0.952	0	0.796	1.004	(0.997,1.011)	0.228	0.793
J34.8 Other specified disorders of nose and nasal sinuses	3	1.006	(0.998,1.013)	0.154	5.936	0.051	0.663	0.991	(0.948,1.036)	0.759	0	0.628	1.005	(1,1.011)	0.054	
J45.9 Asthma, unspecified	3	1.002	(0.995,1.01)	0.531	4.783	0.091	0.582	0.985	(0.948,1.023)	0.574	0.001	0.521	1.002	(0.996,1.008)	0.553	
K01.1 Impacted teeth	3	0.997	(0.993,1.002)	0.248	0.281	0.869	0	0.994	(0.973,1.016)	0.689	0	0.814	0.998	(0.993,1.002)	0.318	
K02.9 Dental caries, unspecified	3	0.996	(0.991,1.001)	0.119	0.011	0.995	0	0.996	(0.972,1.021)	0.81	0	0.992	0.996	(0.99,1.002)	0.169	
K08.3 Retained dental root	3	0.999	(0.995,1.003)	0.578	0.976	0.614	0	1.002	(0.985,1.02)	0.83	0	0.76	0.998	(0.994,1.002)	0.405	
K13.7 Other and unspecified lesions of oral mucosa	3	1.002	(0.998,1.006)	0.264	0.627	0.731	0	0.999	(0.981,1.018)	0.933	0	0.79	1.002	(0.998,1.007)	0.293	
K20 Oesophagitis	6	0.997	(0.991,1.004)	0.438	0.832	0.975	0	0.993	(0.956,1.032)	0.744	0	0.839	0.997	(0.989,1.005)	0.422	0.976
K21.9 Gastro-oesophageal reflux disease without oesophagitis	6	1.002	(0.995,1.009)	0.512	5.859	0.32	0.147	1.013	(0.97,1.058)	0.594	0	0.656	1.002	(0.994,1.011)	0.605	0.395
K22.1 Ulcer of oesophagus	6	1	(0.995,1.006)	0.877	1.623	0.898	0	1.001	(0.971,1.033)	0.939	0	0.959	0.999	(0.993,1.006)	0.86	0.92
K25.9 Unspecified as acute or chronic, without haemorrhage or perforation	3	1.002	(0.997,1.006)	0.416	0.988	0.61	0	1.005	(0.984,1.026)	0.723	0	0.812	1.001	(0.996,1.006)	0.598	
K29.5 Chronic gastritis, unspecified	3	1.004	(1,1.008)	0.078	1.046	0.593	0	1	(0.979,1.021)	0.978	0	0.754	1.005	(1,1.01)	0.064	
K29.6 Other gastritis	3	0.998	(0.994,1.003)	0.518	1.392	0.499	0	1.008	(0.984,1.031)	0.638	0	0.575	0.998	(0.993,1.003)	0.495	
K29.7 Gastritis, unspecified	6	1	(0.993,1.008)	0.976	2.451	0.784	0	0.997	(0.956,1.041)	0.914	0	0.908	0.998	(0.989,1.008)	0.721	0.832
K29.8 Duodenitis	3	0.994	(0.989,0.999)	0.017	2.375	0.305	0.158	1.003	(0.972,1.034)	0.898	0	0.668	0.994	(0.988,0.999)	0.028	
K30 Dyspepsia	6	0.997	(0.989,1.006)	0.515	3.31	0.652	0	1.021	(0.974,1.071)	0.431	-0.001	0.372	0.994	(0.983,1.004)	0.254	0.602
K31.7 Polyp of stomach and duodenum	3	1.004	(1,1.007)	0.049	0.407	0.816	0	1.003	(0.985,1.021)	0.804	0	0.943	1.004	(1.001,1.009)	0.027	
K35.9 Acute appendicitis, unspecified	3	1.001	(0.996,1.005)	0.737	0.723	0.697	0	1.004	(0.982,1.026)	0.795	0	0.832	1	(0.995,1.005)	0.907	
K40.2 Bilateral inguinal hernia, without obstruction or gangrene	3	1.001	(0.997,1.005)	0.518	0.349	0.84	0	1.005	(0.987,1.023)	0.698	0	0.764	1.001	(0.997,1.005)	0.518	
K40.9 Unilateral or unspecified inguinal hernia, without obstruction or gangrene	7	0.996	(0.984,1.007)	0.457	8.38	0.212	0.284	0.979	(0.917,1.044)	0.541	0.001	0.617	0.997	(0.985,1.009)	0.6	0.305
K42.9 Umbilical hernia without obstruction or gangrene	3	1.002	(0.997,1.008)	0.447	0.723	0.697	0	0.996	(0.97,1.022)	0.801	0	0.71	1.002	(0.996,1.008)	0.422	
K43.9 Ventral hernia without obstruction or gangrene	3	0.996	(0.991,1.001)	0.121	0.404	0.817	0	0.997	(0.972,1.023)	0.87	0	0.927	0.996	(0.99,1.002)	0.169	
K44.9 Diaphragmatic hernia without obstruction or gangrene	6	0.991	(0.982,0.999)	0.036	0.477	0.993	0	0.995	(0.948,1.045)	0.852	0	0.872	0.992	(0.982,1.001)	0.09	0.994
K51.9 Ulcerative colitis, unspecified	3	0.991	(0.977,1.005)	0.218	15.646	0	0.872	1.021	(0.946,1.102)	0.69	-0.001	0.582	0.992	(0.986,0.999)	0.022	
K52.9 Non-infective gastro-enteritis and colitis, unspecified	6	0.999	(0.988,1.011)	0.887	7.47	0.188	0.331	1.008	(0.939,1.082)	0.84	0	0.821	0.996	(0.983,1.009)	0.557	0.253
K56.6 Other and unspecified intestinal obstruction	3	0.999	(0.996,1.003)	0.612	1.842	0.398	0	0.995	(0.973,1.018)	0.751	0	0.792	1	(0.996,1.004)	0.877	
K57.3 Diverticular disease of large intestine without perforation or abscess	6	0.99	(0.98,1.001)	0.062	7.104	0.213	0.296	0.969	(0.91,1.031)	0.373	0.001	0.521	0.993	(0.981,1.004)	0.219	0.24
K57.9 Diverticular disease of intestine, part unspecified, without perforation or abscess	3	0.992	(0.984,1.001)	0.066	4.732	0.094	0.577	1.008	(0.961,1.058)	0.802	-0.001	0.63	0.992	(0.986,0.999)	0.029	
K58.9 Irritable bowel syndrome without diarrhoea	3	0.998	(0.993,1.003)	0.512	3.719	0.156	0.462	0.999	(0.965,1.033)	0.948	0	0.992	0.997	(0.992,1.001)	0.173	
K59.0 Constipation	6	0.997	(0.991,1.002)	0.263	3.849	0.571	0	0.989	(0.958,1.02)	0.519	0	0.632	0.995	(0.988,1.001)	0.121	0.638

K60.2 Anal fissure, unspecified	3	0.998	(0.994,1.001)	0.24	1.043	0.594	0	1.005	(0.987,1.023)	0.694	0	0.578	0.998	(0.994,1.002)	0.348	
K60.3 Anal fistula	3	0.996	(0.989,1.003)	0.23	7.009	0.03	0.715	1.017	(1,1.035)	0.301	-0.001	0.245	0.998	(0.993,1.002)	0.247	
K61.0 Anal abscess	3	0.996	(0.989,1.003)	0.265	7.732	0.021	0.741	1.015	(0.986,1.046)	0.497	-0.001	0.416	0.997	(0.993,1.001)	0.165	
K62.1 Rectal polyp	5	1	(0.995,1.006)	0.892	3.247	0.517	0	0.995	(0.966,1.025)	0.755	0	0.734	0.999	(0.992,1.005)	0.681	0.581
K62.5 Haemorrhage of anus and rectum	6	0.992	(0.982,1.002)	0.097	5.765	0.33	0.133	1.02	(0.964,1.08)	0.522	-0.001	0.371	0.992	(0.979,1.005)	0.22	0.307
K62.8 Other specified diseases of anus and rectum	3	0.994	(0.985,1.003)	0.199	8.828	0.012	0.773	1.022	(0.997,1.048)	0.339	-0.001	0.272	0.996	(0.992,1.001)	0.123	
K63.5 Polyp of colon	6	1.002	(0.995,1.008)	0.654	3.166	0.674	0	0.981	(0.945,1.019)	0.381	0.001	0.341	1	(0.992,1.008)	0.996	0.74
K80.0 Calculus of gallbladder with acute cholecystitis	3	1	(0.997,1.004)	0.83	1.624	0.444	0	1.009	(0.991,1.028)	0.495	0	0.502	1	(0.996,1.005)	0.847	
K80.1 Calculus of gallbladder with other cholecystitis	6	0.997	(0.99,1.003)	0.328	6.248	0.283	0.2	1.026	(0.992,1.062)	0.21	-0.001	0.162	0.999	(0.992,1.007)	0.822	0.362
K80.2 Calculus of gallbladder without cholecystitis	6	0.992	(0.985,0.999)	0.034	2.021	0.846	0	1.017	(0.976,1.06)	0.457	-0.001	0.292	0.993	(0.984,1.002)	0.14	0.846
K80.5 Calculus of bile duct without cholangitis or cholecystitis	3	0.997	(0.992,1.002)	0.211	1.897	0.387	0	1.006	(0.981,1.032)	0.716	0	0.601	0.997	(0.991,1.002)	0.197	
K85 Acute pancreatitis	3	1.002	(0.998,1.006)	0.418	0.643	0.725	0	1.003	(0.984,1.022)	0.817	0	0.916	1.002	(0.997,1.006)	0.428	
K92.0 Haematemesis	3	0.999	(0.995,1.003)	0.608	0.903	0.637	0	0.998	(0.98,1.016)	0.872	0	0.936	1	(0.996,1.004)	0.945	
K92.1 Melaena	3	1.001	(0.996,1.006)	0.733	3.137	0.208	0.363	0.987	(0.966,1.009)	0.45	0	0.423	1	(0.996,1.005)	0.925	
K92.2 Gastro-intestinal haemorrhage, unspecified	3	0.993	(0.987,0.999)	0.015	3.666	0.16	0.454	1.006	(0.978,1.036)	0.74	0	0.521	0.993	(0.988,0.998)	0.011	
L03.1 Cellulitis of other parts of limb	6	1.001	(0.995,1.006)	0.847	1.152	0.949	0	0.986	(0.956,1.018)	0.443	0	0.42	1	(0.994,1.007)	0.956	0.959
L72.0 Epidermal cyst	3	1.001	(0.994,1.008)	0.757	3.751	0.153	0.467	1.01	(0.968,1.053)	0.731	0	0.753	1	(0.994,1.006)	0.889	
L72.9 Follicular cyst of skin and subcutaneous tissue, unspecified	3	1.002	(0.998,1.005)	0.416	2.099	0.35	0.047	1.006	(0.983,1.03)	0.705	0	0.769	1.001	(0.997,1.005)	0.592	
L82 Seborrhoeic keratosis	3	1.003	(0.998,1.008)	0.174	0.232	0.89	0	1.006	(0.982,1.031)	0.703	0	0.857	1.003	(0.998,1.009)	0.214	
L90.5 Scar conditions and fibrosis of skin	3	1.001	(0.997,1.005)	0.62	0.319	0.853	0	0.998	(0.977,1.019)	0.865	0	0.8	1.001	(0.997,1.006)	0.581	
L98.9 Disorder of skin and subcutaneous tissue, unspecified	5	0.998	(0.993,1.004)	0.569	1.826	0.768	0	1.015	(0.984,1.047)	0.424	-0.001	0.373	1	(0.993,1.006)	0.955	0.834
M16.1 Other primary coxarthrosis	5	1.002	(0.996,1.008)	0.468	5.294	0.258	0.244	1.019	(0.985,1.054)	0.35	-0.001	0.396	1.004	(0.998,1.011)	0.223	0.35
M16.9 Coxarthrosis, unspecified	6	1.009	(1.003,1.015)	0.005	1.659	0.894	0	1.006	(0.971,1.042)	0.756	0	0.877	1.009	(1.002,1.017)	0.018	0.887
M17.1 Other primary gonarthrosis	6	1.007	(0.999,1.015)	0.092	9.764	0.082	0.488	0.992	(0.945,1.041)	0.756	0	0.57	1.012	(1.004,1.02)	0.003	0.122
M17.9 Gonarthrosis, unspecified	6	1.006	(0.992,1.021)	0.393	17.22	0.004	0.71	0.988	(0.904,1.079)	0.795	0.001	0.695	1.003	(0.993,1.014)	0.529	0.017
M20.1 Hallux valgus (acquired)	6	1.004	(0.997,1.012)	0.222	5.265	0.384	0.05	1.008	(0.964,1.055)	0.743	0	0.879	1.001	(0.992,1.011)	0.755	0.386
M20.2 Hallux rigidus	3	0.996	(0.993,1)	0.053	1.384	0.501	0	1.003	(0.985,1.022)	0.778	0	0.579	0.997	(0.992,1.001)	0.105	
M23.2 Derangement of meniscus due to old tear or injury	3	0.996	(0.992,1.001)	0.136	2.361	0.307	0.153	0.983	(0.963,1.004)	0.356	0	0.419	0.996	(0.991,1.001)	0.082	
M23.22 Derangement of meniscus due to old tear or injury	3	1.003	(0.998,1.008)	0.188	0.6	0.741	0	0.998	(0.975,1.023)	0.916	0	0.751	1.004	(0.998,1.009)	0.206	
M23.23 Derangement of meniscus due to old tear or injury	4	0.996	(0.991,1.001)	0.151	1.066	0.785	0	0.99	(0.962,1.018)	0.538	0	0.684	0.998	(0.991,1.004)	0.484	0.747
M25.5 Pain in joint	3	1	(0.996,1.005)	0.828	0.935	0.627	0	0.991	(0.971,1.012)	0.554	0	0.531	1	(0.995,1.005)	0.977	
M25.56 Pain in joint (Lower leg)	3	0.999	(0.995,1.004)	0.792	1.355	0.508	0	1.007	(0.985,1.029)	0.644	0	0.614	0.999	(0.994,1.005)	0.841	
M51.1 Lumbar and other intervertebral disk disorders with radiculopathy	3	1.003	(0.998,1.008)	0.26	0.817	0.665	0	0.998	(0.972,1.024)	0.888	0	0.748	1.003	(0.997,1.009)	0.278	

M51.2 Other specified intervertebral disk displacement	3	1	(0.995,1.004)	0.852	0.485	0.785	0	1.003	(0.982,1.024)	0.824	0	0.797	0.999	(0.995,1.004)	0.838	
M51.3 Other specified intervertebral disk degeneration	3	1.004	(1,1.008)	0.027	0.811	0.667	0	0.998	(0.98,1.016)	0.854	0	0.609	1.004	(1,1.008)	0.067	
M54.5 Low back pain	4	1	(0.995,1.005)	0.965	2.952	0.399	0	1.006	(0.975,1.039)	0.729	0	0.719	0.999	(0.993,1.005)	0.752	0.53
M54.56 Low back pain (Lumbar region)	3	1.004	(1,1.008)	0.074	0.064	0.968	0	1.006	(0.985,1.027)	0.682	0	0.886	1.004	(0.999,1.008)	0.102	
M54.59 Low back pain (Site unspecified)	3	0.996	(0.991,1.001)	0.15	2.754	0.252	0.274	0.996	(0.962,1.031)	0.855	0	0.985	0.998	(0.993,1.003)	0.462	
M65.3 Trigger finger	3	1.001	(0.997,1.005)	0.556	2.502	0.286	0.201	1.009	(0.985,1.033)	0.605	0	0.644	1.001	(0.996,1.005)	0.765	
M67.4 Ganglion	3	0.997	(0.992,1.002)	0.269	0.045	0.978	0	0.999	(0.974,1.025)	0.974	0	0.88	0.997	(0.991,1.003)	0.344	
M72.0 Palmar fascial fibromatosis [Dupuytren]	3	1.001	(0.995,1.007)	0.68	3.889	0.143	0.486	1.002	(0.963,1.043)	0.935	0	0.972	1.003	(0.997,1.008)	0.324	
M72.04 Palmar fascial fibromatosis [Dupuytren]-Hand	3	1.007	(1,1.014)	0.047	4.711	0.095	0.575	0.998	(0.956,1.041)	0.934	0	0.743	1.007	(1.002,1.013)	0.011	
M75.0 Adhesive capsulitis of shoulder	3	1	(0.993,1.007)	0.945	7.304	0.026	0.726	0.995	(0.949,1.044)	0.872	0	0.863	1.001	(0.996,1.006)	0.577	
M75.1 Rotator cuff syndrome	3	0.997	(0.993,1.002)	0.255	0.137	0.934	0	0.999	(0.976,1.022)	0.92	0	0.93	0.997	(0.992,1.002)	0.261	
M75.4 Impingement syndrome of shoulder	5	1.001	(0.995,1.006)	0.813	1.064	0.9	0	0.999	(0.97,1.029)	0.963	0	0.93	1.001	(0.995,1.007)	0.827	0.893
M79.66 Pain in limb (Lower leg)	3	1	(0.996,1.005)	0.953	0.832	0.66	0	0.994	(0.972,1.017)	0.69	0	0.678	1	(0.995,1.006)	0.87	
M79.86 Other specified soft tissue disorders (Lower leg)	3	1.005	(0.995,1.016)	0.307	10.264	0.006	0.805	0.983	(0.932,1.037)	0.646	0.001	0.557	1.005	(0.999,1.01)	0.105	
N20.0 Calculus of kidney	3	1.004	(0.999,1.01)	0.116	2.174	0.337	0.08	0.989	(0.965,1.015)	0.566	0	0.449	1.004	(0.998,1.01)	0.168	
N20.1 Calculus of ureter	3	1	(0.995,1.005)	0.98	1.795	0.408	0	1.007	(0.979,1.037)	0.708	0	0.7	1	(0.994,1.006)	0.915	
N23 Unspecified renal colic	3	0.998	(0.993,1.004)	0.529	2.821	0.244	0.291	1	(0.963,1.038)	0.989	0	0.951	0.996	(0.99,1.002)	0.175	
N32.0 Bladder-neck obstruction	3	1.003	(0.999,1.007)	0.164	1.117	0.572	0	0.996	(0.976,1.016)	0.748	0	0.602	1.003	(0.998,1.008)	0.255	
N32.8 Other specified disorders of bladder	3	0.997	(0.992,1.003)	0.329	0.143	0.931	0	0.994	(0.968,1.02)	0.71	0	0.817	0.997	(0.992,1.003)	0.327	
N35.9 Urethral stricture, unspecified	3	1	(0.995,1.005)	0.897	0.193	0.908	0	0.996	(0.972,1.021)	0.801	0	0.813	1	(0.994,1.005)	0.878	
N39.0 Urinary tract infection, site not specified	6	0.991	(0.981,1.001)	0.078	10.788	0.056	0.537	1.003	(0.941,1.07)	0.922	0	0.715	0.994	(0.983,1.005)	0.283	0.099
N39.3 Stress incontinence	6	0.997	(0.987,1.007)	0.541	13.358	0.02	0.626	0.983	(0.922,1.047)	0.618	0	0.677	1	(0.991,1.01)	0.968	0.066
N81.1 Cystocele	4	0.999	(0.994,1.004)	0.753	0.222	0.974	0	1.003	(0.975,1.031)	0.874	0	0.83	0.998	(0.992,1.004)	0.581	0.961
N81.6 Rectocele	3	1	(0.996,1.005)	0.895	0.305	0.859	0	0.999	(0.977,1.021)	0.916	0	0.897	1.001	(0.996,1.006)	0.839	

G: Diseases of the nervous system; H: Diseases of the eye and adnexa; I: Diseases of the circulatory system; J: Diseases of the respiratory system; K: Diseases of the digestive system; L: Diseases of the skin and subcutaneous tissue; M: Diseases of the musculoskeletal system and connective tissue; N: Diseases of the genitourinary system.
