# Supplementary material for: Association between human blood metabolome and the risk of gastrointestinal tumors
Source: PLoS One. 2024 May 30;19(5):e0304574. doi: 10.1371/journal.pone.0304574 (PMC11139295; doi:10.1371/journal.pone.0304574)
Supplement: S6 Table — (PDF) [file pone.0304574.s006.pdf]

Supplementary Table 6. Online Sourced Raw Data Catalogue

Stage 1 raw data: identifying the significant blood metabolites as potential causal mediators of gastrointestinal tumors

phenocode	exposure name	path_https
met-d-VLDL_C	VLDL cholesterol	https://gwas.mrcieu.ac.uk/datasets/met-d-VLDL_C/
met-d-Val	Valine	https://gwas.mrcieu.ac.uk/datasets/met-d-Val/
met-d-Tyr	Tyrosine	https://gwas.mrcieu.ac.uk/datasets/met-d-Tyr/
met-d-Total_TG	Total triglycerides	https://gwas.mrcieu.ac.uk/datasets/met-d-Total_TG/
met-d-Total_FC	Total free cholesterol	https://gwas.mrcieu.ac.uk/datasets/met-d-Total_FC/
met-d-Total_FA	Total fatty acids	https://gwas.mrcieu.ac.uk/datasets/met-d-Total_FA/
met-d-Total_CE	Total esterified cholesterol	https://gwas.mrcieu.ac.uk/datasets/met-d-Total_CE/
met-d-Total_C	Total cholesterol	https://gwas.mrcieu.ac.uk/datasets/met-d-Total_C/
met-d-Sphingomyelins	Sphingomyelins	https://gwas.mrcieu.ac.uk/datasets/met-d-Sphingomyelins/
met-d-SFA	Saturated fatty acids	https://gwas.mrcieu.ac.uk/datasets/met-d-SFA/
met-d-Pyruvate	Pyruvate	https://gwas.mrcieu.ac.uk/datasets/met-d-Pyruvate/
met-d-PUFA	Polyunsaturated fatty acids	https://gwas.mrcieu.ac.uk/datasets/met-d-PUFA/
met-d-Phosphoglyc	Phosphoglycerides	https://gwas.mrcieu.ac.uk/datasets/met-d-Phosphoglyc/
met-d-Phosphatidylc	Phosphatidylcholines	https://gwas.mrcieu.ac.uk/datasets/met-d-Phosphatidylc/
met-d-Phe	Phenylalanine	https://gwas.mrcieu.ac.uk/datasets/met-d-Phe/
met-d-MUFA	Monounsaturated fatty acids	https://gwas.mrcieu.ac.uk/datasets/met-d-MUFA/
met-d-Leu	Leucine	https://gwas.mrcieu.ac.uk/datasets/met-d-Leu/
met-d-LDL_C	LDL cholesterol	https://gwas.mrcieu.ac.uk/datasets/met-d-LDL_C/
met-d-Lactate	Lactate	https://gwas.mrcieu.ac.uk/datasets/met-d-Lactate/
met-d-LA	Linoleic acid	https://gwas.mrcieu.ac.uk/datasets/met-d-LA/
met-d-Ile	Isoleucine	https://gwas.mrcieu.ac.uk/datasets/met-d-Ile/
met-d-His	Histidine	https://gwas.mrcieu.ac.uk/datasets/met-d-His/
met-d-HDL_C	HDL cholesterol	https://gwas.mrcieu.ac.uk/datasets/met-d-HDL_C/
met-d-GlycA	Glycoprotein acetyls	https://gwas.mrcieu.ac.uk/datasets/met-d-GlycA/
met-d-Gly	Glycine	https://gwas.mrcieu.ac.uk/datasets/met-d-Gly/
met-d-Glucose	Glucose	https://gwas.mrcieu.ac.uk/datasets/met-d-Glucose/
met-d-Gln	Glutamine	https://gwas.mrcieu.ac.uk/datasets/met-d-Gln/
met-d-DHA	Docosahexaenoic acid	https://gwas.mrcieu.ac.uk/datasets/met-d-DHA/
met-d-Creatinine	Creatinine	https://gwas.mrcieu.ac.uk/datasets/met-d-Creatinine/
met-d-Citrate	Citrate	https://gwas.mrcieu.ac.uk/datasets/met-d-Citrate/
met-d-Cholines	Total cholines	https://gwas.mrcieu.ac.uk/datasets/met-d-Cholines/
met-d-bOHbutyrate	3-Hydroxybutyrate	https://gwas.mrcieu.ac.uk/datasets/met-d-bOHbutyrate/
met-d-ApoB	Apolipoprotein B	https://gwas.mrcieu.ac.uk/datasets/met-d-ApoB/
met-d-ApoA1	Apolipoprotein A1	https://gwas.mrcieu.ac.uk/datasets/met-d-ApoA1/
met-d-Albumin	Albumin	https://gwas.mrcieu.ac.uk/datasets/met-d-Albumin/
met-d-Ala	Alanine	https://gwas.mrcieu.ac.uk/datasets/met-d-Ala/
met-d-Acetone	Acetone	https://gwas.mrcieu.ac.uk/datasets/met-d-Acetone/
met-d-Acetoacetate	Acetoacetate	https://gwas.mrcieu.ac.uk/datasets/met-d-Acetoacetate/
met-d-Acetate	Acetate	https://gwas.mrcieu.ac.uk/datasets/met-d-Acetate/
met-c-862	Glycoproteins	https://gwas.mrcieu.ac.uk/datasets/met-c-862/
met-c-852	22:6, docosahexaenoic acid	https://gwas.mrcieu.ac.uk/datasets/met-c-852/
met-a-753	Cis-4-decenoyl carnitine	https://gwas.mrcieu.ac.uk/datasets/met-a-753/
met-a-747	4-androsten-3beta,17beta-diol disulfate 1	https://gwas.mrcieu.ac.uk/datasets/met-a-747/

met-a-746	5alpha-androstan-3beta,17beta-diol disulfate	https://gwas.mrcieu.ac.uk/datasets/met-a-746/
met-a-743	Tryptophan betaine	https://gwas.mrcieu.ac.uk/datasets/met-a-743/
met-a-742	Succinylcarnitine	https://gwas.mrcieu.ac.uk/datasets/met-a-742/
met-a-735	Octadecanedioate	https://gwas.mrcieu.ac.uk/datasets/met-a-735/
met-a-729	X-14626	https://gwas.mrcieu.ac.uk/datasets/met-a-729/
met-a-720	X-14205--alpha-glutamyltyrosine	https://gwas.mrcieu.ac.uk/datasets/met-a-720/
met-a-712	Dihomo-linolenate (20:3n3 or n6)	https://gwas.mrcieu.ac.uk/datasets/met-a-712/
met-a-711	Hexadecanedioate	https://gwas.mrcieu.ac.uk/datasets/met-a-711/
met-a-709	Tetradecanedioate	https://gwas.mrcieu.ac.uk/datasets/met-a-709/
met-a-699	Glutaroyl carnitine	https://gwas.mrcieu.ac.uk/datasets/met-a-699/
met-a-685	X-13435	https://gwas.mrcieu.ac.uk/datasets/met-a-685/
met-a-684	X-13431--nonanoylcarnitine	https://gwas.mrcieu.ac.uk/datasets/met-a-684/
met-a-683	X-13429	https://gwas.mrcieu.ac.uk/datasets/met-a-683/
met-a-682	1-arachidonoylglycerophosphoethanolamine	https://gwas.mrcieu.ac.uk/datasets/met-a-682/
met-a-667	X-12850	https://gwas.mrcieu.ac.uk/datasets/met-a-667/
met-a-665	X-12844	https://gwas.mrcieu.ac.uk/datasets/met-a-665/
met-a-661	X-12798	https://gwas.mrcieu.ac.uk/datasets/met-a-661/
met-a-656	Bradykinin, des-arg(9)	https://gwas.mrcieu.ac.uk/datasets/met-a-656/
met-a-652	Isovalerylcarnitine	https://gwas.mrcieu.ac.uk/datasets/met-a-652/
met-a-646	X-12728	https://gwas.mrcieu.ac.uk/datasets/met-a-646/
met-a-640	X-12696	https://gwas.mrcieu.ac.uk/datasets/met-a-640/
met-a-638	Asparagine	https://gwas.mrcieu.ac.uk/datasets/met-a-638/
met-a-636	X-12644	https://gwas.mrcieu.ac.uk/datasets/met-a-636/
met-a-634	1-arachidonoylglycerophosphoinositol	https://gwas.mrcieu.ac.uk/datasets/met-a-634/
met-a-633	X-12556	https://gwas.mrcieu.ac.uk/datasets/met-a-633/
met-a-631	Bilirubin (E,Z or Z,E)	https://gwas.mrcieu.ac.uk/datasets/met-a-631/
met-a-629	X-12510--2-aminooctanoic acid	https://gwas.mrcieu.ac.uk/datasets/met-a-629/
met-a-627	Epiandrosterone sulfate	https://gwas.mrcieu.ac.uk/datasets/met-a-627/
met-a-618	Decanoylcarnitine	https://gwas.mrcieu.ac.uk/datasets/met-a-618/
met-a-616	Alpha-hydroxyisovalerate	https://gwas.mrcieu.ac.uk/datasets/met-a-616/
met-a-615	Octanoylcarnitine	https://gwas.mrcieu.ac.uk/datasets/met-a-615/
met-a-613	X-12456	https://gwas.mrcieu.ac.uk/datasets/met-a-613/
met-a-596	X-12244--N-acetylcarnosine	https://gwas.mrcieu.ac.uk/datasets/met-a-596/
met-a-581	X-12093	https://gwas.mrcieu.ac.uk/datasets/met-a-581/
met-a-580	X-12092	https://gwas.mrcieu.ac.uk/datasets/met-a-580/
met-a-578	Erythronate	https://gwas.mrcieu.ac.uk/datasets/met-a-578/
met-a-573	Isobutyrylcarnitine	https://gwas.mrcieu.ac.uk/datasets/met-a-573/
met-a-570	X-12063	https://gwas.mrcieu.ac.uk/datasets/met-a-570/
met-a-560	X-11905	https://gwas.mrcieu.ac.uk/datasets/met-a-560/
met-a-558	1-arachidonoylglycerophosphocholine	https://gwas.mrcieu.ac.uk/datasets/met-a-558/
met-a-543	X-11793--oxidized bilirubin	https://gwas.mrcieu.ac.uk/datasets/met-a-543/
met-a-542	X-11792	https://gwas.mrcieu.ac.uk/datasets/met-a-542/
met-a-541	X-11787	https://gwas.mrcieu.ac.uk/datasets/met-a-541/
met-a-536	X-11593--O-methylascorbate	https://gwas.mrcieu.ac.uk/datasets/met-a-536/
met-a-531	X-11538	https://gwas.mrcieu.ac.uk/datasets/met-a-531/
met-a-529	X-11530	https://gwas.mrcieu.ac.uk/datasets/met-a-529/
met-a-528	X-11529	https://gwas.mrcieu.ac.uk/datasets/met-a-528/

met-a-526	HWESASXX	https://gwas.mrcieu.ac.uk/datasets/met-a-526/
met-a-524	X-11491	https://gwas.mrcieu.ac.uk/datasets/met-a-524/
met-a-519	X-11469	https://gwas.mrcieu.ac.uk/datasets/met-a-519/
met-a-517	X-11445--5-alpha-pregnan-3beta,20alpha-disulfate	https://gwas.mrcieu.ac.uk/datasets/met-a-517/
met-a-516	X-11444	https://gwas.mrcieu.ac.uk/datasets/met-a-516/
met-a-515	X-11442	https://gwas.mrcieu.ac.uk/datasets/met-a-515/
met-a-514	X-11441	https://gwas.mrcieu.ac.uk/datasets/met-a-514/
met-a-513	X-11440	https://gwas.mrcieu.ac.uk/datasets/met-a-513/
met-a-510	X-09789	https://gwas.mrcieu.ac.uk/datasets/met-a-510/
met-a-505	X-03056--N-[3-(2-Oxopyrrolidin-1-yl)propyl]acetamide	https://gwas.mrcieu.ac.uk/datasets/met-a-505/
met-a-501	Pyroglutamine	https://gwas.mrcieu.ac.uk/datasets/met-a-501/
met-a-500	3-dehydrocarnitine	https://gwas.mrcieu.ac.uk/datasets/met-a-500/
met-a-495	X-11315	https://gwas.mrcieu.ac.uk/datasets/met-a-495/
met-a-491	Bilirubin (E,E)	https://gwas.mrcieu.ac.uk/datasets/met-a-491/
met-a-490	X-11261	https://gwas.mrcieu.ac.uk/datasets/met-a-490/
met-a-485	X-02269	https://gwas.mrcieu.ac.uk/datasets/met-a-485/
met-a-484	X-11204	https://gwas.mrcieu.ac.uk/datasets/met-a-484/
met-a-482	10-undecenoate (11:1n1)	https://gwas.mrcieu.ac.uk/datasets/met-a-482/
met-a-479	Propionylcarnitine	https://gwas.mrcieu.ac.uk/datasets/met-a-479/
met-a-476	Butyrylcarnitine	https://gwas.mrcieu.ac.uk/datasets/met-a-476/
met-a-467	Hexanoylcarnitine	https://gwas.mrcieu.ac.uk/datasets/met-a-467/
met-a-464	Serine	https://gwas.mrcieu.ac.uk/datasets/met-a-464/
met-a-460	Androsterone sulfate	https://gwas.mrcieu.ac.uk/datasets/met-a-460/
met-a-450	Bilirubin (Z,Z)	https://gwas.mrcieu.ac.uk/datasets/met-a-450/
met-a-449	N-acetylglycine	https://gwas.mrcieu.ac.uk/datasets/met-a-449/
met-a-444	X-10510	https://gwas.mrcieu.ac.uk/datasets/met-a-444/
met-a-435	X-08988	https://gwas.mrcieu.ac.uk/datasets/met-a-435/
met-a-427	2-hydroxyisobutyrate	https://gwas.mrcieu.ac.uk/datasets/met-a-427/
met-a-426	X-08402	https://gwas.mrcieu.ac.uk/datasets/met-a-426/
met-a-419	1,5-anhydroglucitol (1,5-AG)	https://gwas.mrcieu.ac.uk/datasets/met-a-419/
met-a-396	X-18601	https://gwas.mrcieu.ac.uk/datasets/met-a-396/
met-a-383	3-methyl-2-oxovalerate	https://gwas.mrcieu.ac.uk/datasets/met-a-383/
met-a-381	N-acetylornithine	https://gwas.mrcieu.ac.uk/datasets/met-a-381/
met-a-379	Carnitine	https://gwas.mrcieu.ac.uk/datasets/met-a-379/
met-a-375	Kynurenine	https://gwas.mrcieu.ac.uk/datasets/met-a-375/
met-a-373	X-03094	https://gwas.mrcieu.ac.uk/datasets/met-a-373/
met-a-362	Betaine	https://gwas.mrcieu.ac.uk/datasets/met-a-362/
met-a-360	Gamma-glutamyltyrosine	https://gwas.mrcieu.ac.uk/datasets/met-a-360/
met-a-357	Biliverdin	https://gwas.mrcieu.ac.uk/datasets/met-a-357/
met-a-356	Citrulline	https://gwas.mrcieu.ac.uk/datasets/met-a-356/
met-a-355	Proline	https://gwas.mrcieu.ac.uk/datasets/met-a-355/
met-a-345	Urate	https://gwas.mrcieu.ac.uk/datasets/met-a-345/
met-a-339	4-acetamidobutanoate	https://gwas.mrcieu.ac.uk/datasets/met-a-339/
met-a-319	Arachidonate (20:4n6)	https://gwas.mrcieu.ac.uk/datasets/met-a-319/
met-a-316	Uridine	https://gwas.mrcieu.ac.uk/datasets/met-a-316/
met-a-314	Mannose	https://gwas.mrcieu.ac.uk/datasets/met-a-314/
met-a-304	Tryptophan	https://gwas.mrcieu.ac.uk/datasets/met-a-304/

phenocode	outcome name	path_https
finn-b-C3_COLORECTAL_EXALLC	Colorectal cancer	https://gwas.mrcieu.ac.uk/datasets/finn-b-C3_COLORECTAL_EXALLC
finn-b-C3_LIVER_INTRAHEPATIC_BILE_DUCTS_EXALLC	Liver cancer	https://gwas.mrcieu.ac.uk/datasets/finn-b-C3_LIVER_INTRAHEPATIC_
finn-b-C3_OESOPHAGUS_EXALLC	Esophageal cancer	https://gwas.mrcieu.ac.uk/datasets/finn-b-C3_OESOPHAGUS_EXALLC
finn-b-C3_STOMACH_EXALLC	Stomach cancer	https://gwas.mrcieu.ac.uk/datasets/finn-b-C3_STOMACH_EXALLC/
finn-b-C3_PANCREAS_EXALLC	Pancreatic cancer	https://gwas.mrcieu.ac.uk/datasets/finn-b-C3_PANCREAS_EXALLC/

Stage 2 raw data: Phe-MR analysis associations of identified blood metabolite on the risk of 118 diseases

phenocode	exposure name	path_https
met-a-652	Isovalerylcarnitine	https://gwas.mrcieu.ac.uk/datasets/met-a-652/
**phenocode**	**outcome name**	**path_https**
ukb-b-10362	K80.0 Calculus of gallbladder with acute cholecystitis	https://gwas.mrcieu.ac.uk/datasets/ukb-b-10362/
ukb-b-10859	K57.3 Diverticular disease of large intestine without perforation or abscess	https://gwas.mrcieu.ac.uk/datasets/ukb-b-10859/
ukb-b-10873	M54.59 Low back pain	https://gwas.mrcieu.ac.uk/datasets/ukb-b-10873/
ukb-b-11020	K80.2 Calculus of gallbladder without cholecystitis	https://gwas.mrcieu.ac.uk/datasets/ukb-b-11020/
ukb-b-11261	I84.9 Unspecified haemorrhoids without complication	https://gwas.mrcieu.ac.uk/datasets/ukb-b-11261/
ukb-b-11274	K60.2 Anal fissure, unspecified	https://gwas.mrcieu.ac.uk/datasets/ukb-b-11274/
ukb-b-11298	N81.1 Cystocele	https://gwas.mrcieu.ac.uk/datasets/ukb-b-11298/
ukb-b-11369	K40.2 Bilateral inguinal hernia, without obstruction or gangrene	https://gwas.mrcieu.ac.uk/datasets/ukb-b-11369/
ukb-b-11403	L90.5 Scar conditions and fibrosis of skin	https://gwas.mrcieu.ac.uk/datasets/ukb-b-11403/
ukb-b-11582	K62.5 Haemorrhage of anus and rectum	https://gwas.mrcieu.ac.uk/datasets/ukb-b-11582/
ukb-b-11748	I47.1 Supraventricular tachycardia	https://gwas.mrcieu.ac.uk/datasets/ukb-b-11748/
ukb-b-11771	K08.3 Retained dental root	https://gwas.mrcieu.ac.uk/datasets/ukb-b-11771/
ukb-b-11908	L03.1 Cellulitis of other parts of limb	https://gwas.mrcieu.ac.uk/datasets/ukb-b-11908/
ukb-b-12047	I20.0 Unstable angina	https://gwas.mrcieu.ac.uk/datasets/ukb-b-12047/
ukb-b-12267	I80.2 Phlebitis and thrombophlebitis of other deep vessels of lower extremities	https://gwas.mrcieu.ac.uk/datasets/ukb-b-12267/
ukb-b-123	N32.0 Bladder-neck obstruction	https://gwas.mrcieu.ac.uk/datasets/ukb-b-123/
ukb-b-12316	K29.8 Duodenitis	https://gwas.mrcieu.ac.uk/datasets/ukb-b-12316/
ukb-b-12364	M67.4 Ganglion	https://gwas.mrcieu.ac.uk/datasets/ukb-b-12364/
ukb-b-12397	H26.9 Cataract, unspecified	https://gwas.mrcieu.ac.uk/datasets/ukb-b-12397/
ukb-b-12620	M79.86 Other specified soft tissue disorders	https://gwas.mrcieu.ac.uk/datasets/ukb-b-12620/
ukb-b-12872	M51.2 Other specified intervertebral disk displacement	https://gwas.mrcieu.ac.uk/datasets/ukb-b-12872/
ukb-b-13019	M25.5 Pain in joint	https://gwas.mrcieu.ac.uk/datasets/ukb-b-13019/
ukb-b-1306	K29.6 Other gastritis	https://gwas.mrcieu.ac.uk/datasets/ukb-b-1306/
ukb-b-13190	G43.9 Migraine, unspecified	https://gwas.mrcieu.ac.uk/datasets/ukb-b-13190/
ukb-b-13391	G45.9 Transient cerebral ischaemic attack, unspecified	https://gwas.mrcieu.ac.uk/datasets/ukb-b-13391/
ukb-b-13464	K62.8 Other specified diseases of anus and rectum	https://gwas.mrcieu.ac.uk/datasets/ukb-b-13464/
ukb-b-13517	M72.0 Palmar fascial fibromatosis	https://gwas.mrcieu.ac.uk/datasets/ukb-b-13517/
ukb-b-13731	K22.1 Ulcer of oesophagus	https://gwas.mrcieu.ac.uk/datasets/ukb-b-13731/
ukb-b-1386	K21.9 Gastro-oesophageal reflux disease without oesophagitis	https://gwas.mrcieu.ac.uk/datasets/ukb-b-1386/
ukb-b-1418	M75.4 Impingement syndrome of shoulder	https://gwas.mrcieu.ac.uk/datasets/ukb-b-1418/
ukb-b-14206	I20.9 Angina pectoris, unspecified	https://gwas.mrcieu.ac.uk/datasets/ukb-b-14206/
ukb-b-14416	I84.1 Internal haemorrhoids with other complications	https://gwas.mrcieu.ac.uk/datasets/ukb-b-14416/
ukb-b-14436	L72.9 Follicular cyst of skin and subcutaneous tissue, unspecified	https://gwas.mrcieu.ac.uk/datasets/ukb-b-14436/
ukb-b-14541	K42.9 Umbilical hernia without obstruction or gangrene	https://gwas.mrcieu.ac.uk/datasets/ukb-b-14541/

ukb-b-14814	K30 Dyspepsia	https://gwas.mrcieu.ac.uk/datasets/ukb-b-14814/
ukb-b-14999	K92.2 Gastro-intestinal haemorrhage, unspecified	https://gwas.mrcieu.ac.uk/datasets/ukb-b-14999/
ukb-b-15003	L72.0 Epidermal cyst	https://gwas.mrcieu.ac.uk/datasets/ukb-b-15003/
ukb-b-1557	M54.5 Low back pain	https://gwas.mrcieu.ac.uk/datasets/ukb-b-1557/
ukb-b-15606	J18.9 Pneumonia, unspecified	https://gwas.mrcieu.ac.uk/datasets/ukb-b-15606/
ukb-b-16313	N23 Unspecified renal colic	https://gwas.mrcieu.ac.uk/datasets/ukb-b-16313/
ukb-b-1668	I25.1 Atherosclerotic heart disease	https://gwas.mrcieu.ac.uk/datasets/ukb-b-1668/
ukb-b-16781	G47.3 Sleep apnoea	https://gwas.mrcieu.ac.uk/datasets/ukb-b-16781/
ukb-b-16882	I84.6 Residual haemorrhoidal skin tags	https://gwas.mrcieu.ac.uk/datasets/ukb-b-16882/
ukb-b-17961	K58.9 Irritable bowel syndrome without diarrhoea	https://gwas.mrcieu.ac.uk/datasets/ukb-b-17961/
ukb-b-17992	I84.2 Internal haemorrhoids without complication	https://gwas.mrcieu.ac.uk/datasets/ukb-b-17992/
ukb-b-18279	M51.1 Lumbar and other intervertebral disk disorders with radiculopathy	https://gwas.mrcieu.ac.uk/datasets/ukb-b-18279/
ukb-b-18366	I26.9 Pulmonary embolism without mention of acute cor pulmonale	https://gwas.mrcieu.ac.uk/datasets/ukb-b-18366/
ukb-b-18372	N20.0 Calculus of kidney	https://gwas.mrcieu.ac.uk/datasets/ukb-b-18372/
ukb-b-18379	M17.9 Gonarthrosis, unspecified	https://gwas.mrcieu.ac.uk/datasets/ukb-b-18379/
ukb-b-1853	K92.0 Haematemesis	https://gwas.mrcieu.ac.uk/datasets/ukb-b-1853/
ukb-b-18629	N20.1 Calculus of ureter	https://gwas.mrcieu.ac.uk/datasets/ukb-b-18629/
ukb-b-18785	K13.7 Other and unspecified lesions of oral mucosa	https://gwas.mrcieu.ac.uk/datasets/ukb-b-18785/
ukb-b-18822	K29.7 Gastritis, unspecified	https://gwas.mrcieu.ac.uk/datasets/ukb-b-18822/
ukb-b-19045	J34.8 Other specified disorders of nose and nasal sinuses	https://gwas.mrcieu.ac.uk/datasets/ukb-b-19045/
ukb-b-19350	I63.9 Cerebral infarction, unspecified	https://gwas.mrcieu.ac.uk/datasets/ukb-b-19350/
ukb-b-19354	K20 Oesophagitis	https://gwas.mrcieu.ac.uk/datasets/ukb-b-19354/
ukb-b-19386	K51.9 Ulcerative colitis, unspecified	https://gwas.mrcieu.ac.uk/datasets/ukb-b-19386/
ukb-b-19388	K85 Acute pancreatitis	https://gwas.mrcieu.ac.uk/datasets/ukb-b-19388/
ukb-b-1968	K63.5 Polyp of colon	https://gwas.mrcieu.ac.uk/datasets/ukb-b-1968/
ukb-b-19805	K62.1 Rectal polyp	https://gwas.mrcieu.ac.uk/datasets/ukb-b-19805/
ukb-b-19807	M51.3 Other specified intervertebral disk degeneration	https://gwas.mrcieu.ac.uk/datasets/ukb-b-19807/
ukb-b-19813	M54.56 Low back pain	https://gwas.mrcieu.ac.uk/datasets/ukb-b-19813/
ukb-b-19829	M16.9 Coxarthrosis, unspecified	https://gwas.mrcieu.ac.uk/datasets/ukb-b-19829/
ukb-b-20189	K52.9 Non-infective gastro-enteritis and colitis, unspecified	https://gwas.mrcieu.ac.uk/datasets/ukb-b-20189/
ukb-b-20190	L98.9 Disorder of skin and subcutaneous tissue, unspecified	https://gwas.mrcieu.ac.uk/datasets/ukb-b-20190/
ukb-b-20208	J45.9 Asthma, unspecified	https://gwas.mrcieu.ac.uk/datasets/ukb-b-20208/
ukb-b-2083	M25.56 Pain in joint	https://gwas.mrcieu.ac.uk/datasets/ukb-b-2083/
ukb-b-2285	H25.0 Senile incipient cataract	https://gwas.mrcieu.ac.uk/datasets/ukb-b-2285/
ukb-b-2875	M16.1 Other primary coxarthrosis	https://gwas.mrcieu.ac.uk/datasets/ukb-b-2875/
ukb-b-3095	H25.1 Senile nuclear cataract	https://gwas.mrcieu.ac.uk/datasets/ukb-b-3095/
ukb-b-3469	I21.9 Acute myocardial infarction, unspecified	https://gwas.mrcieu.ac.uk/datasets/ukb-b-3469/
ukb-b-373	N32.8 Other specified disorders of bladder	https://gwas.mrcieu.ac.uk/datasets/ukb-b-373/
ukb-b-3770	J34.2 Deviated nasal septum	https://gwas.mrcieu.ac.uk/datasets/ukb-b-3770/
ukb-b-3965	G56.0 Carpal tunnel syndrome	https://gwas.mrcieu.ac.uk/datasets/ukb-b-3965/
ukb-b-3983	I25.9 Chronic ischaemic heart disease, unspecified	https://gwas.mrcieu.ac.uk/datasets/ukb-b-3983/
ukb-b-4280	M20.2 Hallux rigidus	https://gwas.mrcieu.ac.uk/datasets/ukb-b-4280/
ukb-b-453	I21.0 Acute transmural myocardial infarction of anterior wall	https://gwas.mrcieu.ac.uk/datasets/ukb-b-453/
ukb-b-4637	H00.1 Chalazion	https://gwas.mrcieu.ac.uk/datasets/ukb-b-4637/
ukb-b-4738	K25.9 Unspecified as acute or chronic, without haemorrhage or perforation	https://gwas.mrcieu.ac.uk/datasets/ukb-b-4738/
ukb-b-4770	K02.9 Dental caries, unspecified	https://gwas.mrcieu.ac.uk/datasets/ukb-b-4770/
ukb-b-4772	M72.04 Palmar fascial fibromatosis	https://gwas.mrcieu.ac.uk/datasets/ukb-b-4772/

ukb-b-4921	M75.0 Adhesive capsulitis of shoulder	https://gwas.mrcieu.ac.uk/datasets/ukb-b-4921/
ukb-b-50	M75.1 Rotator cuff syndrome	https://gwas.mrcieu.ac.uk/datasets/ukb-b-50/
ukb-b-501	L82 Seborrhoeic keratosis	https://gwas.mrcieu.ac.uk/datasets/ukb-b-501/
ukb-b-5018	K61.0 Anal abscess	https://gwas.mrcieu.ac.uk/datasets/ukb-b-5018/
ukb-b-5126	I21.1 Acute transmural myocardial infarction of inferior wall	https://gwas.mrcieu.ac.uk/datasets/ukb-b-5126/
ukb-b-5369	K40.9 Unilateral or unspecified inguinal hernia, without obstruction or gangrene	https://gwas.mrcieu.ac.uk/datasets/ukb-b-5369/
ukb-b-5590	K92.1 Melaena	https://gwas.mrcieu.ac.uk/datasets/ukb-b-5590/
ukb-b-6026	M20.1 Hallux valgus	https://gwas.mrcieu.ac.uk/datasets/ukb-b-6026/
ukb-b-6082	N81.6 Rectocele	https://gwas.mrcieu.ac.uk/datasets/ukb-b-6082/
ukb-b-6576	J18.1 Lobar pneumonia, unspecified	https://gwas.mrcieu.ac.uk/datasets/ukb-b-6576/
ukb-b-6716	K29.5 Chronic gastritis, unspecified	https://gwas.mrcieu.ac.uk/datasets/ukb-b-6716/
ukb-b-6720	I83.9 Varicose veins of lower extremities without ulcer or inflammation	https://gwas.mrcieu.ac.uk/datasets/ukb-b-6720/
ukb-b-6721	K60.3 Anal fistula	https://gwas.mrcieu.ac.uk/datasets/ukb-b-6721/
ukb-b-677	K43.9 Ventral hernia without obstruction or gangrene	https://gwas.mrcieu.ac.uk/datasets/ukb-b-677/
ukb-b-6779	K59.0 Constipation	https://gwas.mrcieu.ac.uk/datasets/ukb-b-6779/
ukb-b-686	K35.9 Acute appendicitis, unspecified	https://gwas.mrcieu.ac.uk/datasets/ukb-b-686/
ukb-b-7118	M23.23 Derangement of meniscus due to old tear or injury	https://gwas.mrcieu.ac.uk/datasets/ukb-b-7118/
ukb-b-7166	K57.9 Diverticular disease of intestine, part unspecified, without perforation or a	https://gwas.mrcieu.ac.uk/datasets/ukb-b-7166/
ukb-b-7211	J33.9 Nasal polyp, unspecified	https://gwas.mrcieu.ac.uk/datasets/ukb-b-7211/
ukb-b-7330	K31.7 Polyp of stomach and duodenum	https://gwas.mrcieu.ac.uk/datasets/ukb-b-7330/
ukb-b-7444	I84.8 Unspecified haemorrhoids with other complications	https://gwas.mrcieu.ac.uk/datasets/ukb-b-7444/
ukb-b-7534	K01.1 Impacted teeth	https://gwas.mrcieu.ac.uk/datasets/ukb-b-7534/
ukb-b-7700	M17.1 Other primary gonarthrosis	https://gwas.mrcieu.ac.uk/datasets/ukb-b-7700/
ukb-b-8085	M65.3 Trigger finger	https://gwas.mrcieu.ac.uk/datasets/ukb-b-8085/
ukb-b-8213	M23.2 Derangement of meniscus due to old tear or injury	https://gwas.mrcieu.ac.uk/datasets/ukb-b-8213/
ukb-b-8268	K80.5 Calculus of bile duct without cholangitis or cholecystitis	https://gwas.mrcieu.ac.uk/datasets/ukb-b-8268/
ukb-b-8707	J22 Unspecified acute lower respiratory infection	https://gwas.mrcieu.ac.uk/datasets/ukb-b-8707/
ukb-b-8814	N39.0 Urinary tract infection, site not specified	https://gwas.mrcieu.ac.uk/datasets/ukb-b-8814/
ukb-b-8848	K44.9 Diaphragmatic hernia without obstruction or gangrene	https://gwas.mrcieu.ac.uk/datasets/ukb-b-8848/
ukb-b-8988	K80.1 Calculus of gallbladder with other cholecystitis	https://gwas.mrcieu.ac.uk/datasets/ukb-b-8988/
ukb-b-9074	N35.9 Urethral stricture, unspecified	https://gwas.mrcieu.ac.uk/datasets/ukb-b-9074/
ukb-b-9565	H02.8 Other specified disorders of eyelid	https://gwas.mrcieu.ac.uk/datasets/ukb-b-9565/
ukb-b-964	I48 Atrial fibrillation and flutter	https://gwas.mrcieu.ac.uk/datasets/ukb-b-964/
ukb-b-9694	M23.22 Derangement of meniscus due to old tear or injury	https://gwas.mrcieu.ac.uk/datasets/ukb-b-9694/
ukb-b-9708	M79.66 Pain in limb	https://gwas.mrcieu.ac.uk/datasets/ukb-b-9708/
ukb-b-9797	K56.6 Other and unspecified intestinal obstruction	https://gwas.mrcieu.ac.uk/datasets/ukb-b-9797/
ukb-b-9873	N39.3 Stress incontinence	https://gwas.mrcieu.ac.uk/datasets/ukb-b-9873/
