# Supplementary material for: Association between human blood metabolome and the risk of gastrointestinal tumors
Source: PLoS One. 2024 May 30;19(5):e0304574. doi: 10.1371/journal.pone.0304574 (PMC11139295; doi:10.1371/journal.pone.0304574)
Supplement: S7 Table — (PDF) [file pone.0304574.s007.pdf]

Supplementary Table 7. Potentially relevant plasma metabolites.

**Liver Cancer**	
**Exposure**	***P-value***
Sphingomyelins	8.89E-05
Total free cholesterol	0.000400268
Docosahexaenoic acid	0.001811286
Albumin	0.002319058
X-11538	0.004936789
Polyunsaturated fatty acids	0.005370436
Octadecanedioate	0.005683321
Creatinine	0.009473667
Hexadecanedioate	0.014772756
Glycoproteins	0.024452914
X-11593--O-methylascorbate	0.027455936
Propionylcarnitine	0.028717972
X-12844	0.036987877
Pyruvate	0.044751821
**Colorectal Cancer**	
**Exposure**	***P-value***
Arachidonate (20:4n6)	0.002209543
X-08402	0.000589278
Bilirubin (Z,Z)	0.034656614
X-11792	0.040956521
X-12556	0.021893742
X-12696	0.005898757
Bradykinin, des-arg(9)	0.039353048
Acetate	0.002564406
Citrate	0.005560711
Docosahexaenoic acid	0.028861975
Glutamine	0.003797563
Arachidonate (20:4n6)	0.026158701
**Esophageal Cancer**	
**Exposure**	***P-value***
X-03056--N-[3-(2-Oxopyrrolidin-1-yl)propyl]acetamide	0.018350697
X-09789	0.040966858
X-12844	0.014040831
5alpha-androstan-3beta,17beta-diol disulfate	0.029460923
Acetoacetate	0.031957143
Acetone	0.011930866
Total cholines	0.023691613
Glycoprotein acetyls	0.047155029
Histidine	0.005026028

Phosphatidylcholines	0.010659466
Phosphoglycerides	0.019716384
Total free cholesterol	0.047393401
**Gastric Cancer**	
**Exposure**	***P-value***
Citrulline	0.03830828
Biliverdin	0.008601059
Bilirubin (Z,Z)	0.002633253
Bilirubin (E,E)	0.004592473
X-11441	0.005392541
X-11442	0.006509524
X-11530	0.010152495
X-11593--O-methylascorbate	0.023335691
X-11793--oxidized bilirubin	0.009846255
Erythronate	0.021057905
Bilirubin (E,Z or Z,E)	0.004783061
X-12556	0.038863305
Acetone	0.009303623
Apolipoprotein B	0.001663642
Docosahexaenoic acid	0.008936826
Histidine	0.005249933
LDL cholesterol	0.000281781
Total cholesterol	0.006307196
Total esterified cholesterol	0.006373139
Total free cholesterol	0.026576067
**Pancreatic Cancer**	
**Exposure**	***P-value***
Urate	0.005951156
X-08402	0.025969453
X-02269	0.0137362
X-11469	0.00431628
X-11593--O-methylascorbate	0.010994793
X-13431--nonanoylcarnitine	0.041065759
Acetate	0.020611482
Albumin	0.029347672
Isoleucine	0.020157163
Lactate	0.03556879
Polyunsaturated fatty acids	0.024439669
Pyruvate	0.032481363
Saturated fatty acids	0.019931648
Total cholesterol	0.009816065
Total esterified cholesterol	0.01647866
Total fatty acids	0.026469673

Total free cholesterol	0.027121672
